# Group sequential methods for the Mann-Whitney parameter

**DOI:** 10.1177/09622802221107103

**Published:** 2022-06-13

**Authors:** Claus P Nowak, Tobias Mütze, Frank Konietschke

**Affiliations:** 1 Charité – Universitätsmedizin Berlin, Freie Universität Berlin and Humboldt-Universität zu Berlin, Institute of Biometry and Clinical Epidemiology, Berlin, Germany; 2 TU Dortmund University, Faculty of Statistics, Dortmund, Germany; 3 Statistical Methodology, 1528 Novartis Pharma AG, Basel, Switzerland

**Keywords:** Brunner-Munzel test, error spending, group sequential methods, nonparametric relative effect, Wilcoxon-Mann-Whitney test, win odds

## Abstract

Late phase clinical trials are occasionally planned with one or more interim analyses to allow for early termination or adaptation of the study. While extensive theory has been developed for the analysis of ordered categorical data in terms of the Wilcoxon-Mann-Whitney test, there has been comparatively little discussion in the group sequential literature on how to provide repeated confidence intervals and simple power formulas to ease sample size determination. Dealing more broadly with the nonparametric Behrens-Fisher problem, we focus on the comparison of two parallel treatment arms and show that the Wilcoxon-Mann-Whitney test, the Brunner-Munzel test, as well as a test procedure based on the log win odds, a modification of the win ratio, asymptotically follow the canonical joint distribution. In addition to developing power formulas based on these results, simulations confirm the adequacy of the proposed methods for a range of scenarios. Lastly, we apply our methodology to the FREEDOMS clinical trial (ClinicalTrials.gov Identifier: NCT00289978) in patients with relapse-remitting multiple sclerosis.

## 1 Introduction

Since it is not uncommon for phase III clinical trials to run for a number of years, there is much interest in being able to assess safety and efficacy while the trial is still ongoing. Unsurprisingly, regulatory authorities (EMA,^1^ FDA^2^) point out the need to adequately address multiplicity issues and give practical guidance on group sequential methods, which allow for repeated significance testing on accumulating data without inflating the nominal overall type I error rate.

While standard textbooks such as Jennison and Turnbull^3^, Proschan,^4^ or Wassmer and Brannath^5^ primarily discuss continuous, binary and survival endpoints, the Wilcoxon-Mann-Whitney test^6–8^ has also been extended to group sequential settings.^9–11^ In our view, the estimand most naturally associated with the Wilcoxon-Mann-Whitney test is the probability

$$p=P(X_{1}<X_{2})+1/2\cdot P(X_{1}=X_{2})$$

where $X_{1}∼F_{1}$ and $X_{2}∼F_{2}$ denote two independent random variables. The quantity p is called nonparametric relative effect of $X_{2}$ with respect to $X_{1}$ , probabilistic index or Mann-Whitney parameter.^12–15^ Dividing p by its complement produces

p/(1−p)

the so-called win odds.^16^ Adding half of the probability of equal outcomes to $P(X_{1}<X_{2})$ neatly aligns with Putter’s generalisation^17^ of the Wilcoxon-Mann-Whitney test to the case of ties. By the same token, Brunner et al.^16^ regard the win odds to be a tie corrected version of the win ratio $P(X_{1}<X_{2})/P(X_{1}>X_{2})$ , which has recently attracted attention in the context of time-to-event data,^18^ continuous endpoints,^19^ and stratification.^20^ Of course, if tied values cannot occur almost surely, that is, if $P(X_{1}=X_{2})=0$ , then p equals $P(X_{1}<X_{2})$ and the win odds coincide with the win ratio.

To illustrate the interpretation of the nonparametric relative effect p , let us assume that $X_{1}$ and $X_{2}$ refer to outcomes from treatment arms 1 and 2, respectively, and that lower values point to a more favourable outcome. Then p is nothing but the probability that patients on arm 1 will fare better than those on arm 2, including 1/2 times the probability of equal outcomes. Perhaps a little easier to interpret are the win odds. For instance, if p=0.75 , then the odds that a patient on arm 1 will fare better than one on arm 2 are 3:1 , with the possibility of equal outcomes equally allocated to the ‘fare better’ and ‘fare worse’ scenarios.

However, asymptotic results of the Wilcoxon-Mann-Whitney test as commonly employed are only valid if both distributions coincide, that is, if $F_{1}=F_{2}$ . Hence the null hypothesis is usually formulated in terms of the distribution functions as well, that is, $H_{0}\;:\;F_{1}=F_{2}$ and not the Mann-Whitney parameter p as such. While $F_{1}=F_{2}$ implies p=1/2 , the reverse does not hold. For instance, any two symmetric distributions with the same centre of symmetry, such as two normal distributions N(0,1) and N(0,4) , would imply p=1/2 . In essence, the nonparametric Behrens-Fisher problem addresses the testing problem $H_{0}\;:\;p=1/2$ , while making no further assumptions on $F_{1}$ and $F_{2}$ , which is precisely the scenario that the Brunner-Munzel test^12^ was developed to deal with. In that regard, unlike the Wilcoxon-Mann-Whitney test, the limiting distribution of the Brunner-Munzel test is normal with unit variance under both the null and the alternative hypotheses, thus allowing for test inversion and computation of confidence intervals for p , which in turn facilitates the derivation of simple power approximations in the group sequential setting.

A key tool in group sequential theory which we will also rely on here is the so-called *canonical joint distribution.*^3–5,21^ More precisely, a sequence of K test statistics $\left\{Z_{1},\ldots ,Z_{K}\right\}$ with information levels $\left\{I_{1},\ldots ,I_{K}\right\}$ for a single parameter θ are said to follow the *canonical joint distribution* if

-
$Z=(Z_{1},\ldots ,Z_{K})$ follows a multivariate normal distribution,
-
$E(Z_{k})=\theta \sqrt{I_{k}},\;k=1,\ldots ,K$ ,
-
$C\mathrm{ov}(Z_{k_{1}},Z_{k_{2}})=\sqrt{I_{k_{1}}/I_{k_{2}}},\;1\leq k_{1}\leq k_{2}\leq K.$

As might be expected, group sequential versions of the nonparametric tests just discussed follow the canonical joint distribution only asymptotically, which is why we will check its applicability for finite sample sizes by way of extensive simulations.

This paper is organised as follows. Section 2 introduces notation and group sequential methods for hypothesis tests based on the nonparametric relative effect p , with derivations concerning the covariance structure of the corresponding group sequential statistics Z referred to the appendix. Following a discussion on error spending in Section 3, we set out results from simulation studies in Section 4 to assess type I error rates for finite sample sizes. Section 5 deals with the retrospective application of our proposed methodology to a completed clinical trial, whereas Section 6 outlines how to plan a group sequential trial with the aid of simple approximate power formulas. More detailed results and technical considerations regarding the simulations are provided in the Supplemental Material.

## 2 Nonparametric group sequential models

We start with notation from nonparametric theory necessary to develop group sequential models for the Wilcoxon-Mann-Whitney test, the Brunner-Munzel test and a *logit* transformed version of the latter, which we refer to as the log win odds test. With the asymptotic normality of the test statistics at issue already established for the fixed sample size scenario, a vector Z of such statistics based on accumulating groups of data is asymptotically multivariate normal by the Crámer-Wold theorem.^22^ Thus, in order to obtain the asymptotic joint distribution, it remains to properly define the information levels and derive the expectation and covariance matrix of Z .

### 2.1 Notation

Let X be a univariate random variable representing real-valued or ordered categorical data, defined on the probability space (Ω,A,P) . Adopting common notation, we denote by

$$\begin{array}{rl} F^{-}(x) & =P(X<x)\;\text{ the \textbackslash{};left-continuous}\\ F^{+}(x) & =P(X\leq x)\;\text{ the \textbackslash{};right-continuous}\\ F(x) & =P(X<x)+1/2\cdot P(X=x)\;\text{ the \textbackslash{},\textbackslash{},normalised} \end{array}$$

version of the cumulative distribution function of X .^23,24,12^

Now suppose we have a sample of observations $X_{1},\ldots ,X_{n}\overset{iid}{∼}F$ . Then we call

$$\begin{array}{rl} \hat{F}(x) & =\frac{1}{n}\sum_{\;j=1}^{n}c(x,X_{\;j}),\;c(x,X_{\;j})=\left\{ \begin{array}{cc} 0 & \text{ if }x<X_{\;j}\\ 1/2 & \text{ if }x=X_{\;j}\\ 1 & \text{ if }x>X_{\;j} \end{array}\right. \end{array}$$

the normalised version of the empirical cumulative distribution function. Moreover,

$$R_{i}=1/2+\sum_{\;j=1}^{n}c(X_{i},X_{\;j}),\;i=1,\ldots ,n$$

denotes the mid-rank of $X_{i}$ among the observations $X_{1},\ldots ,X_{n}$ .

For two independent random variables $X_{1}∼F_{1}$ and $X_{2}∼F_{2}$ , the probability

$$p=P(X_{1}<X_{2})+1/2\cdot P(X_{1}=X_{2})=\int F_{1}dF_{2}$$

is called nonparametric relative effect of $X_{2}$ with respect to $X_{1}$ (or of $F_{2}$ with respect to $F_{1}$ ). We say that

-
$X_{1}$ tends to smaller values than $X_{2}$ if p>1/2 ,
-
$X_{1}$ tends to larger values than $X_{2}$ if p<1/2 ,
-
$X_{1}$ and $X_{2}$ are stochastically comparable if p=1/2 .

For a more comprehensive discussion on nonparametric theory we refer to Brunner et al.^13^

Throughout the remainder of this paper we will focus on a parallel two-arm clinical trial and consider accumulating responses

$$\begin{array}{rl} X_{1i} & \overset{iid}{∼}F_{1},\;i=1,2,\ldots \\ X_{2j} & \overset{iid}{∼}F_{2},\;j=1,2,\ldots \end{array}$$

from treatment arms 1 and 2, respectively. Apart from assuming that 0<p<1 and that there exists no x such that $P(X_{1i}=x)=1$ or $P(X_{2j}=x)=1$ , which excludes the degenerate cases of completely separated samples and one-point distributions, $F_{1}$ and $F_{2}$ are otherwise arbitrary.

With $n_{1k}$ and $n_{2k}$ denoting the cumulative number of observations available at analysis k=1,…,K for the respective treatments, $N_{k}=n_{1k}+n_{2k}$ , we can estimate the nonparametric relative effect p by

$${\hat{\;p}}^{\;(k)}=\int {\hat{F}}_{1}^{\left(k\right)}d{\hat{F}}_{2}^{\left(k\right)}=\frac{1}{n_{1k}}\frac{1}{n_{2k}}\sum_{\;j=1}^{n_{2k}}\sum_{i=1}^{n_{1k}}c(X_{2j},X_{1i})=\frac{1}{N_{k}}\left({\bar{R}}_{2∙}^{\left(k\right)}-{\bar{R}}_{1∙}^{\left(k\right)}\right)+1/2$$

with ${\bar{R}}_{g∙}^{\left(k\right)}=\frac{1}{n_{gk}}\sum_{i=1}^{n_{gk}}R_{gi}^{\left(k\right)}$ , where $R_{gi}^{\left(k\right)}$ is the mid-rank of $X_{gi}$ among all observations

$$X_{11},\ldots ,X_{1n_{1k}},X_{21},\ldots ,X_{2n_{2k}}$$

available at analysis k ; g=1,2 ; $i=1,\ldots ,n_{gk}$ .

For asymptotic results, we let both sample sizes tend to infinity such that neither vanishes, that is, $n_{gk}/N_{k}\to \gamma_{g}>0$ for both $n_{1k}\to \infty$ and $n_{2k}\to \infty$ , g=1,2 .

### 2.2 Wilcoxon-Mann-Whitney test allowing for ties

To test the hypothesis $H_{0}\;:\;F_{1}=F_{2}$ against $H_{1}\;:\;F_{1}\neq F_{2}$ , we employ at each interim analysis k the same test statistic as in the fixed design, namely

(1) $${\hat{Z}}_{k}=\left({\hat{\;p}}^{\;(k)}-1/2\right)\sqrt{{\hat{I}}_{k}},\;k=1,\ldots ,K$$

with estimated information ${\hat{I}}_{k}=(N_{k}n_{1k}n_{2k})/{\hat{\sigma }}_{Rk}^{2}$ , where

$${\hat{\sigma }}_{Rk}^{2}=\frac{1}{N_{k}-1}\sum_{g=1}^{2}\sum_{i=1}^{n_{gk}}{\left(R_{gi}^{\left(k\right)}-\frac{N_{k}+1}{2}\right)}^{2},\;k=1,\ldots ,K$$

It is well known that each ${\hat{Z}}_{k}$ converges in distribution to a standard normal random variate, provided the null hypothesis is true.^13^

To derive the asymptotic joint distribution of $\hat{Z}=({\hat{Z}}_{1},\ldots ,{\hat{Z}}_{k})$ we need to compute its covariance matrix. Proceeding in accord with Jennison and Turnbull,^3^ we first replace the estimated information with its population version, resulting in

(2) $$Z_{k}=\left({\hat{\;p}}^{\;(k)}-1/2\right)\sqrt{I_{k}}\;\overset{D}{\underset{H_{0}}{\to }}\;\;\;N(0,1),\;k=1,\ldots ,K$$

(3) $$I_{k}=\left(N_{k}n_{1k}n_{2k}\right)/\sigma_{Rk}^{2}$$

where we assume the variance

(4) $$\sigma_{Rk}^{2}=N_{k}\left\{\left(N_{k}-2\right)\int F^{2}dF-\frac{N_{k}-3}{4}\right\}-\frac{N_{k}}{4}\int \left(F^{+}-F^{-}\right)dF$$

and therefore the true distribution $F=F_{1}=F_{2}$ to be known.^13^ If F is continuous, the information simplifies to $I_{k}={\hat{I}}_{k}=(12n_{1k}n_{2k})/(N_{k}+1)$ .

Since ${\hat{\sigma }}_{Rk}^{2}$ are consistent estimators of $\sigma_{Rk}^{2}$ , k=1,…,K , the vector of Wilcoxon-Mann-Whitney test statistics $\hat{Z}$ has the same limiting distribution as its counterpart $Z=(Z_{1},\ldots ,Z_{K})$ with the true population information. The limiting distribution being multivariate normal, it remains to establish the covariances of the components of Z .

Proposition 1. Let $Z_{k}$ and $I_{k}$ be defined as in (2) and (3). Then, for $1\leq k_{1}\leq k_{2}\leq K$ ,

$$C\mathrm{ov}\left(Z_{k_{1}},Z_{k_{2}}\right)=\sqrt{I_{k_{1}}/I_{k_{2}}}$$

### 2.3 Brunner-Munzel test

To test the null hypothesis $H_{0}\;:\;p=1/2$ against $H_{1}\;:\;p\neq 1/2$ , we now compute, analogous to before, for each interim analysis k the Brunner-Munzel test statistic

(5) $${\hat{Z}}_{k}=\left({\hat{\;p}}^{\;(k)}-1/2\right)\sqrt{{\hat{I}}_{k}},\;k=1,\ldots ,K$$

with estimated information ${\hat{I}}_{k}=({\hat{\sigma }}_{1k}^{2}/n_{1k}+{\hat{\sigma }}_{2k}^{2}/n_{2k}{)}^{-1}$ , where

$$\begin{array}{rl} {\hat{\sigma }}_{1k}^{2} & =\frac{1}{n_{2k}^{2}(n_{1k}-1)}\sum_{i=1}^{n_{1k}}{\left(R_{1i}^{\left(k\right)}-R_{1i}^{\left(1k\right)}-{\bar{R}}_{1∙}^{\left(k\right)}+\frac{n_{1}+1}{2}\right)}^{2}\\ {\hat{\sigma }}_{2k}^{2} & =\frac{1}{n_{1k}^{2}(n_{2k}-1)}\sum_{\;j=1}^{n_{2k}}{\left(R_{2j}^{\left(k\right)}-R_{2j}^{\left(2k\right)}-{\bar{R}}_{2∙}^{\left(k\right)}+\frac{n_{2}+1}{2}\right)}^{2} \end{array}$$

and $R_{gi}^{\left(gk\right)}$ denotes the mid-rank of $X_{gi}$ among the observations of the g th treatment group $X_{g1},\ldots ,X_{gn_{gk}}$ available at analysis k ; g=1,2 ; $i=1,\ldots ,n_{gk}$ .

For the derivation of the asymptotic covariance, we take an approach similar to before. Once again, we substitute the estimated information with the true one

(6) $$\begin{array}{rl} Z_{k} & =\left({\hat{\;p}}^{\;(k)}-1/2\right)\sqrt{I_{k}}\;\overset{D}{\to }\;\;\;N\left(\theta \sqrt{I_{k}},1\right),\;k=1,\ldots ,K\\ \theta & =p-1/2\\ I_{k} & ={\left(\sigma_{1}^{2}/n_{1k}+\sigma_{2}^{2}/n_{2k}\right)}^{-1} \end{array}$$

where $\sigma_{1}^{2}=V\{F_{2}(X_{1i})\}$ and $\sigma_{2}^{2}=V\{(F_{1}(X_{2j})\}$ . However, since the definition of the variance components $\sigma_{1}^{2}$ and $\sigma_{2}^{2}$ is actually based on an asymptotically equivalent version of the $Z_{k}$ s, that is to say,

(7) $$Z_{k}^{U}=\left\{\frac{1}{n_{2k}}\sum_{\;j=1}^{n_{2k}}F_{1}\left(X_{2j}\right)-\frac{1}{n_{1k}}\sum_{i=1}^{n_{1k}}F_{2}\left(X_{1i}\right)\right\}\sqrt{I_{k}}\;\overset{D}{\to }\;N\left(\theta \sqrt{I_{k}},1\right)$$

we compute the covariance accordingly. This result is given in the following proposition.

Proposition 2. Let $Z_{k}^{U}$ and $I_{k}$ be defined as in (7) and (6). Then, for $1\leq k_{1}\leq k_{2}\leq K$ ,

$$C\mathrm{ov}\left(Z_{k_{1}}^{U},Z_{k_{2}}^{U}\right)=\sqrt{I_{k_{1}}/I_{k_{2}}}$$

Thus, ${\hat{I}}_{k}$ consistently estimating $I_{k}$ , k=1,…,K , the sequence of Brunner-Munzel test statistics $\left\{{\hat{Z}}_{1},\ldots ,{\hat{Z}}_{K}\right\}$ asymptotically follow the canonical joint distribution. In the nonsequential scenario, the test has been shown to be too liberal for small sample sizes when using standard normal quantiles.^12^ Analogous to the parametric Behrens-Fisher problem, they propose a Satterthwaite-Smith-Welch t -approximation^25–27^ with degrees of freedom estimated by

(8) $${\hat{\nu }}_{k}=\frac{\{{\hat{\sigma }}_{1k}^{2}/n_{1k}+{\hat{\sigma }}_{2k}^{2}/n_{2k}{\}}^{2}}{{\hat{\sigma }}_{1k}^{4}/\left\{n_{1k}^{2}\left(n_{1k}-1\right)\right\}+{\hat{\sigma }}_{2k}^{4}/\left\{n_{2k}^{2}\left(n_{2k}-1\right)\right\}}$$

Another way is to employ a variance stabilising transformation, such as the *logit* function, producing the logarithmised win odds, which we will explore in the next subsection.

### 2.4 Log win odds test

To address the liberal behaviour of the Brunner-Munzel test, we now consider

$$\begin{array}{rl} \psi & =\ln\;\left\{\;p/\left(1-p\right)\right\}\\ {\hat{\psi }}^{\;(k)} & =\ln\;\left\{{\hat{\;p}}^{\;(k)}/\left(1-{\hat{\;p}}^{\;(k)}\right)\right\} \end{array}$$

at stage k=1,…,K . Consequently, straightforward application of the delta method yields

(9) $${\hat{Z}}_{k}=\left({\hat{\psi }}^{\;(k)}-0\right)\sqrt{{\hat{I}}_{k}}\;\overset{D}{\to }\;N\left(\theta \sqrt{I_{k}},1\right),\;k=1,\ldots ,K$$

(10) $$Z_{k}=\left({\hat{\psi }}^{\;(k)}-0\right)\sqrt{I_{k}}\;\overset{D}{\to }\;\;\;N\left(\theta \sqrt{I_{k}},1\right),\;k=1,\ldots ,K$$

with effect θ=ψ−0 and information levels

$$\begin{array}{rl} I_{k} & =\frac{{\left\{\;p\left(1-p\right)\right\}}^{2}}{\sigma_{1}^{2}/n_{1k}+\sigma_{2}^{2}/n_{2k}}\\ {\hat{I}}_{k} & =\frac{{\left\{{\hat{\;p}}^{\;(k)}\left(1-{\hat{\;p}}^{\;(k)}\right)\right\}}^{2}}{{\hat{\sigma }}_{1k}^{2}/n_{1k}+{\hat{\sigma }}_{2k}^{2}/n_{2k}} \end{array}$$

which is nothing but $\{p(1-p){\}}^{2}$ times, or $\{{\hat{\;p}}^{\;(k)}(1-{\hat{\;p}}^{\;(k)}){\}}^{2}$ times, the information for the corresponding effect p−1/2 from the Brunner-Munzel test as in Section 2.3. Moreover, Proposition 2 together with the information obtained by the delta method directly imply that the log win odds test statistics asymptotically follow the canonical joint distribution.

To recapitulate, in all three cases under the respective assumptions, the standardised test statistics $\left\{Z_{1},\ldots ,Z_{K}\right\}$ with information $\left\{I_{1},\ldots ,I_{K}\right\}$ for the parameter θ asymptotically follow the *canonical joint distribution*. The difference between the Wilcoxon-Mann-Whitney and Brunner-Munzel tests arises solely from the way in which we define the information, both distributions $F_{1}$ and $F_{2}$ needing to coincide for the former but not the latter. The log win odds test is nothing but a Brunner-Munzel test based on the *logit* transformed nonparametric relative effect p .

Before we investigate the adequacy of the proposed methods by means of simulations, we turn our discussion to error spending to explain in more detail the manner in which we wish to reject the null hypothesis.

## 3 Error spending

Initially, group sequential methods required the number of interim looks to be specified in advance and equally spaced: Pocock^28^ considered standard normal test statistics and derived local significance levels (‘stage levels’) which are identical across all stages, while O’Brien and Fleming^29^ stage levels are extremely low at the first interim and increase with each stage in such a way that the final stage level is quite close to the nominal overall significance level α . To avoid having to specify the time or number of interim looks in advance, Lan and DeMets^30^ suggested the use of error spending functions, which we will employ in the simulations.

With statistics and information levels $Z_{k}$ , ${\hat{Z}}_{k}$ , $I_{k}$ , ${\hat{I}}_{k}$ , k=1,…,K , given as in the previous section, a right-sided group sequential test for efficacy maintains the nominal significance level α if the stage levels $\alpha_{1},\ldots ,\alpha_{K}$ are chosen such that

(11) $$\alpha =P_{H_{0}}\left(\;p_{k}\leq \alpha_{k}\;\text{ for \textbackslash{} some \textbackslash{} }k=1,\ldots ,K\right)$$

where we regard the repeated p -values $p_{k}=1-\Phi ({\hat{Z}}_{k})$ , k=1,…,K , to be random variables, Φ denoting the cumulative distribution function of the standard normal distribution. The null hypothesis is rejected at stage k if $p_{k}\leq \alpha_{k}$ and the trial is consequently stopped. We do not, however, set up futility bounds.

To obtain specific stage levels, we split the global α into K positive parts $\pi_{k}$ (‘ α spent at stage k ’), k=1,…,K , such that $\sum_{k=1}^{K}\pi_{k}=\alpha$ and

$$P_{H_{0}}\left(\;p_{1}>\alpha_{1},\ldots ,p_{k-1}>\alpha_{k-1},p_{k}\leq \alpha_{k}\right)=\pi_{k}$$

To compute the stage levels $\alpha_{1},\ldots ,\alpha_{k}$ , we make use of the underlying limiting *canonical joint distribution* of the statistics $\left\{{\hat{Z}}_{1},\ldots ,{\hat{Z}}_{k}\right\}$ and estimate the covariance of ${\hat{Z}}_{k}$ and ${\hat{Z}}_{K}$ by $\sqrt{{\hat{I}}_{k}/I_{max}}$ , k=1,…,K−1 , where $I_{max}$ is the prespecified information that we believe would be available if the total maximum sample size $N_{K}$ of the trial were observed under the respective treatment allocation scheme.

The error spending function prescribes precisely how the global α is to be spent across the stages. More formally, an error spending function is defined as a nondecreasing function f:[0,∞[→[0,α] such that f(0)=0 and f(t)=α for all t≥1 . Then the amount of α allocated to stages k=1,…,K is given by

$$\begin{array}{rl} \pi_{1} & =f(I_{1}/I_{K})\\ \pi_{2} & =f(I_{k}/I_{K})-f(I_{k-1}/I_{K}),\;k=2,\ldots ,K \end{array}$$

However, the true information levels are not known in advance. Therefore, we use $I_{max}$ instead of $I_{K}$ and replace the other information levels by their estimates,

$$\begin{array}{rl} \pi_{1} & =f\left({\hat{I}}_{1}/I_{max}\right)\\ \pi_{2} & =f\left({\hat{I}}_{k}/I_{max}\right)-f\left({\hat{I}}_{k-1}/I_{max}\right),\;k=2,\ldots ,K-1\\ \pi_{K} & =\alpha -f\left({\hat{I}}_{K-1}/I_{max}\right) \end{array}$$

As ${\hat{I}}_{K}$ might turn out to be lower than $I_{max}$ , the last equation ensures that the full amount of α still available is spent at the last stage. Moreover, it is important to bear in mind that the information levels ${\hat{I}}_{k}$ are estimated at stage k and remain unchanged thereafter.

## 4 Simulations

As the methods developed in Section 2 are of asymptotic nature, we explore their applicability for finite sample sizes in a range of scenarios. To this end, we simulate the group sequential Wilcoxon-Mann-Whitney, Brunner-Munzel, and log win odds tests given as in (1), (5), and (9), respectively. Assuming that lower values correspond to more favourable outcomes, we want to show that treatment 1 is superior to treatment 2, yielding a one-sided efficacy test with $H_{0}\;:\;p\leq 1/2$ against $H_{1}\;:\;p>1/2$ and a nominal overall significance level of α=0.025 . In that regard, it is perhaps more natural to view the Wilcoxon-Mann-Whitney test as a means to test the null hypothesis $H_{0}\;:\;p\leq 1/2$ as well, with $F_{1}=F_{2}$ constituting a model assumption under the null.

To gauge the type I error rate of our proposed methods, we perform 100,000 simulation runs for each scenario, giving rise to a Monte Carlo error of about 0.0003 based on a 95%-precision interval for a global α=0.025 . Altogether, we present the results of 120 scenarios for each data generating process, that is all combinations of

- total maximum sample sizes $N_{K}=\{144,288,576,864,1008\},$
- allocation ratios 1:1 or 2:1 (twice as many patients on treatment arm 1),
- two, three, or four stages, and
- two error spending functions.

More specifically, we consider O’Brien and Fleming^29^ as well as Pocock^28^ type error spending functions

$$\begin{array}{rl} \;f_{OF}(t) & =\min\left\{2-2\Phi \left(\frac{z_{1-\alpha /2}}{\sqrt{t}}\right),\alpha \right\}\\ f_{PO}(t) & =\min\left[\alpha \ln\;\left\{1+\left(e-1\right)t\right\},\alpha \right] \end{array}$$

using the information fractions ${\hat{I}}_{k}/I_{K}$ , k=1,…,K to determine the amount of α to be spent since we know the true maximum information $I_{K}$ . For the subsequent computation of the stage levels, we make use of the command getDesignGroupSequential() from the R package rpact .^31^ In addition to using standard normal quantiles for the Wilcoxon-Mann-Whitney, Brunner-Munzel, and log win odds tests, we compute rejection rates based on the Satterthwaite-Smith-Welch t -approximation for the Brunner-Munzel test. As is suggested by Jennison and Turnbull^3^ and Wassmer and Brannath^5^ to provide satisfactorily accurate results for the two sample t -test, we use the same stage levels for the t -approximation and change the computation of the repeated p -values only, namely $p_{k}=1-F_{{\hat{\nu }}_{k}}({\hat{Z}}_{k})$ , where $F_{{\hat{\nu }}_{k}}$ denotes the cumulative distribution function of the t -distribution with ${\hat{\nu }}_{k}$ degrees of freedom as in (8).

It might occur that our methods break down, for instance the variance estimate of the Brunner-Munzel test might be zero in finite samples or the estimated information could actually decrease in a subsequent stage. Since this happened very rarely and has virtually no influence on the results presented in the main paper, we relegate the discussion on exception handling to the supplementary material. Moreover, we only report the overall type I error rate here, that is, the relative frequency of simulation runs, where the null hypothesis could be rejected at some stage. Readers interested in a more detailed presentation of the results such as cumulative rejection rates for each stage are again referred to the supplementary material.

### 4.1 Normal distribution

**Figure 1.**
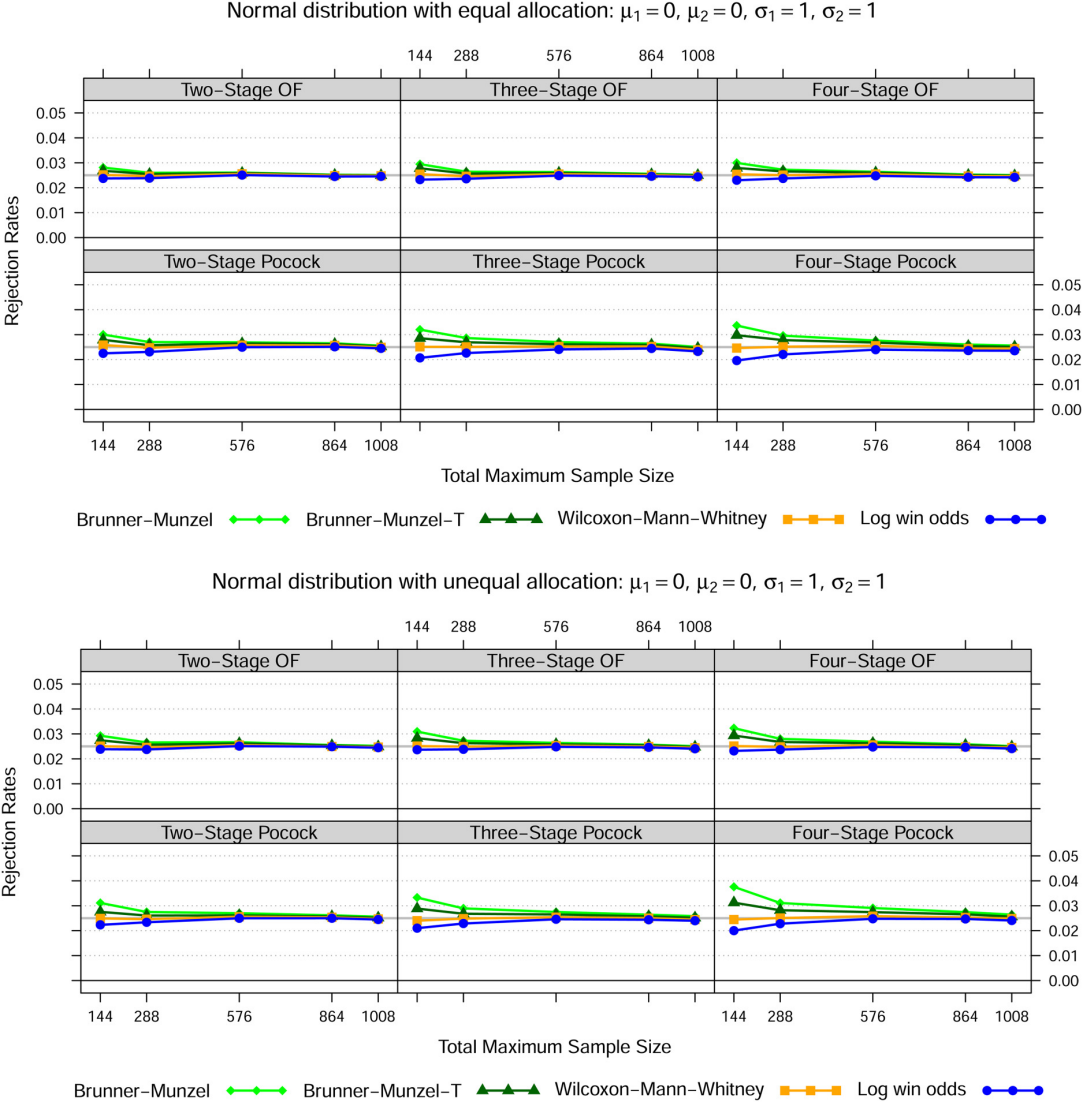
Normal distribution—Setting 1 Notes: The lines show the relative frequency of the 100000 simulation runs, where the null hypothesis could be rejected at some stage based on the Brunner-Munzel test (with t-approximation) as in (5), the Wilcoxon-Mann-Whitney test as in (1) and the log win odds test as in (9) for five different total maximum sample sizes, two error spending functions, up to four stages in total as well as two different allocation ratios.

**Figure 2.**
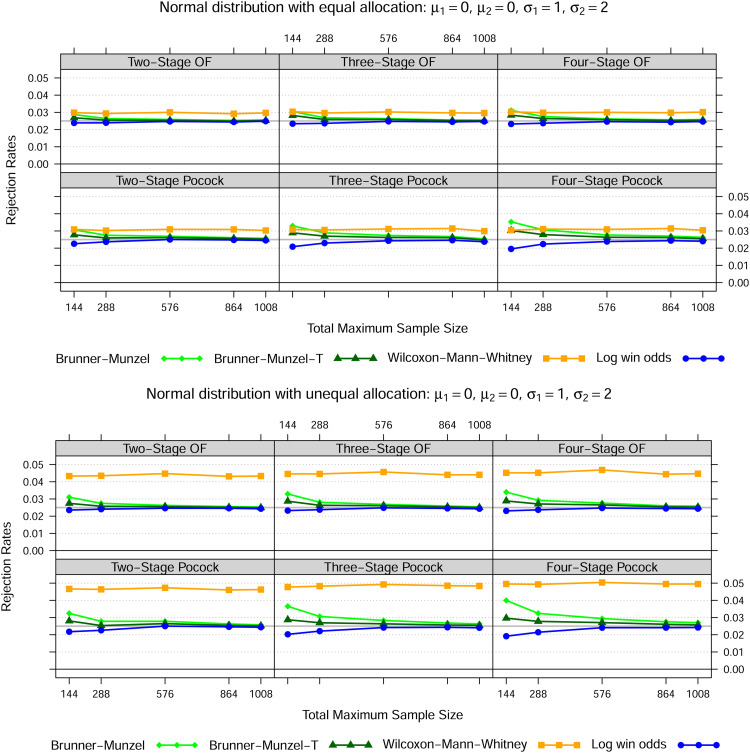
Normal distribution—Setting 2 Notes: The lines show the relative frequency of the 100000 simulation runs, where the null hypothesis could be rejected at some stage based on the Brunner-Munzel test (with t-approximation) as in (5), the Wilcoxon-Mann-Whitney test as in (1) and the log win odds test as in (9) for five different total maximum sample sizes, two error spending functions, up to four stages in total as well as two different allocation ratios.

**Figure 3.**
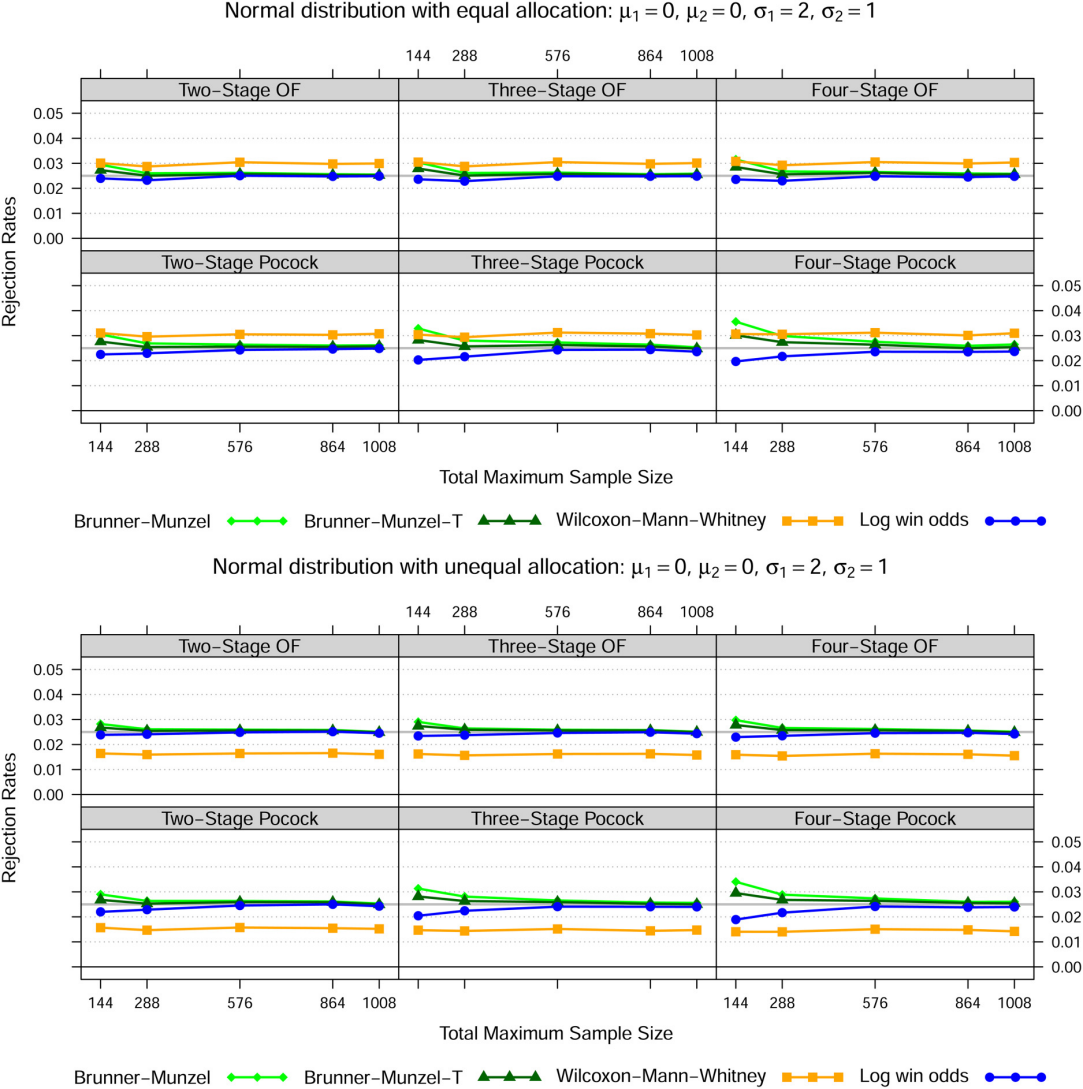
Normal distribution—Setting 3 Notes: The lines show the relative frequency of the 100000 simulation runs, where the null hypothesis could be rejected at some stage based on the Brunner-Munzel test (with t-approximation) as in (5), the Wilcoxon-Mann-Whitney test as in (1) and the log win odds test as in (9) for five different total maximum sample sizes, two error spending functions, up to four stages in total as well as two different allocation ratios.

First we generated data from normal distributions, namely $X_{gi}\overset{iid}{∼}N(\mu_{g},\sigma_{g}^{2})$ , g=1,2 , $i=1,\ldots ,n_{g}$ , for three different settings as set out in Figures 1 to 3. In case of equal variances, the Wilcoxon-Mann-Whitney test best maintains the nominal type I error rate for all total maximum sample sizes, whereas the Brunner-Munzel test with or without t -approximation tends to be too liberal and the log win odds test too conservative for smaller samples sizes. In both heteroskedastic settings, that is settings 2 and 3, the Wilcoxon-Mann-Whitney test exceeds the nominal significance level across all sample sizes if the allocation ratio is 1:1. However, if twice as many patients receive treatment 1, then the Wilcoxon-Mann-Whitney test is far too liberal if the data in treatment 1 is less dispersed than in treatment 2 and far too conservative conversely. Again, this behaviour is not affected by sample size.

In line with the simulation results of Brunner and Munzel^12^ for the fixed sample size scenario, the rejection rates pattern of the other tests are not affected by heteroskedasticity or different allocation schemes.

### 4.2 Ordinal data

Now we consider ordinal data divided into five categories $C_{1}<C_{2}<C_{3}<C_{4}<C_{5}$ , with a smaller index pointing to a more favourable outcome. As in Brunner et al.,^16^ the probabilities of each category occurring are derived through a latent Beta distribution: Let $Y_{gi}\overset{iid}{∼}\mathrm{Beta}(\alpha_{g},\beta_{g})$ , g=1,2 , $i=1,\ldots ,n_{g}$ , denote a Beta distributed random variable with shape parameters $\alpha_{g},\beta_{g}>0$ , such that the expectation and variance of $Y_{gi}$ are given by

$$E(Y_{gi})=\frac{\alpha_{g}}{\alpha_{g}+\beta_{g}},\;V(Y_{gi})=\frac{\alpha_{g}\beta_{g}}{\left(\alpha_{g}+\beta_{g}{)}^{2}(\alpha_{g}+\beta_{g}+1\right)}$$

Then, the random variable $X_{gi}$ , g=1,2 , $i=1,\ldots ,n_{g}$ , is defined by

$$X_{gi}=C_{k}\text{\textbackslash{}, if }\;Y_{gi}\in \left[0.2(k-1),0.2k\right]\text{\textbackslash{}, for }\;k=1,\ldots ,5$$

Consequently, the probability mass function of $X_{gi}$ is nothing but

$$P(X_{gi}=C_{k})=P\left\{0.2(k-1)\leq Y_{gi}<0.2k\right\}\text{ \textbackslash{},for }\;k=1,\ldots ,5$$

We specify three different parameter settings to mimic the homo-/heteroskedasticity pattern for the normal scenarios in Section 4.1. The results exhibit virtually the same behaviour as the normally distributed responses shown previously and are therefore included in the online supplementary material.

## 5 FREEDOMS clinical trial

The FREEDOMS clincial trial (ClinicalTrials.gov Identifier: NCT00289978) was a placebo-controlled phase III study running from January 2006 to July 2009 to analyse the efficacy and safety of fingolimod in patients with relapsing-remitting multiple sclerosis.^32^ The primary efficacy endpoint was the annualised relapse rate at 24 months after baseline evaluation. The definition of a relapse was based on the Expanded Disability Status Scale (EDSS),^33^ with values ranging from 0 (normal status) to 10 (death due to multiple sclerosis) and a step size of 0.5, although a value of 0.5 is not possible. Thus, a higher score on the EDSS indicates more severe disability.

In this paper, we focus on the EDSS score at 24 months, its change compared to the baseline (post minus prae), and its direction of change, that is, whether the EDSS score at 24 month decreased ( −1 ), stayed the same ( 0 ), or increased ( +1 ) with respect to the baseline value. To simplify the presentation of the results, we only considered the complete cases data set, that is, patients where the EDSS score was observed both at baseline and 24 months thereafter. Summary descriptive statistics depicted in Table 1 reveal in all three cases that, at the end of the trial, the mean EDSS outcome of patients on the placebo arm is higher and therefore less favourable than for those on the fingolimod 0.5 mg treatment.

While the original design of the FREEDOMS trial did not provide for interim looks, we now retrospectively analyse the data as though there were two equally spaced stages. More specifically, the first 353 patients on either arm who completed the 24 month evaluation form the basis of the first stage analysis, while all 706 patients are taken into account at the second and therefore last stage. As we did in the simulation section, we consider the Wilcoxon-Mann-Whitney test, the Brunner-Munzel test (with t -approximation) as well as the log win odds test and employ O’Brien and Fleming as well as Pocock type error spending functions. Since we do this analysis retrospectively, we can choose $I_{max}={\hat{I}}_{2}$ . In all scenarios the estimated information fractions ${\hat{I}}_{1}/{\hat{I}}_{2}$ are close to 0.5 , essentially coinciding with the sample size fraction 353/706 .

**Table 1.** Summary descriptive statistics for EDSS data at month 24, month 24 minus baseline (change), and direction of change from the FREEDOMS clinical trial.

EDSS	Treatment	n	Mean	SD	Min	Median	Max
Month 24	Fingolimod 0.5 mg	374	2.269	1.442	0	2	6.5
	Placebo	332	2.545	1.507	0	2	7.0
Change	Fingolimod 0.5 mg	374	0.004	0.878	−3	0	3.5
	Placebo	332	0.131	0.936	−3	0	3.5
Direction	Fingolimod 0.5 mg	374	−0.078	0.734	−1	0	1
	Placebo	332	0.099	0.769	−1	0	1

Analogous to the simulation section, we aim to reject $H_{0}\;:\;p\leq 1/2$ at a global significance level of 2.5% . As Tables 2 to 4 demonstrate, we can reject the null hypothesis at some stage in any scenario and conclude that fingolimod treatment is efficacious. Only the direction of change endpoint leads to early rejection, that is, when using Pocock type stage levels. Even if the trial could not have been stopped at the interim, second stage p -values in the region of 0.1% would have resulted in rejection eventually. Consistent with the results from the simulations, the p -values and confidence intervals from different tests are fairly close.

**Table 2.** Repeated effect estimates, p -values in % based on standard normal and t approximation (T), O’Brien and Fleming ( $\alpha_{OF}$ ) and Pocock type ( $\alpha_{P}$ ) error spending stage levels in %.

			Wilcoxon-Mann-Whitney			Brunner-Munzel			Log win odds		
EDSS	N	Estimate	p -value	$\alpha_{OF}$	$\alpha_{PO}$	p -value (T)	$\alpha_{OF}$	$\alpha_{PO}$	p -value	$\alpha_{OF}$	$\alpha_{PO}$
Month 24	353	0.545	7.20	0.16	1.56	7.19 (7.23)	0.15	1.54	7.29	0.16	1.56
	706	0.558	0.34^**^	2.45	1.38	0.33^**^ (0.33^**^)	2.45	1.39	0.35^**^	2.45	1.38
Change	353	0.564	1.60	0.14	1.53	1.60 (1.63)	0.14	1.53	1.69	0.14	1.52
	706	0.560	0.21^**^	2.45	1.39	0.20^**^ (0.21^**^)	2.45	1.40	0.22^**^	2.46	1.40
Direction	353	0.565	1.21^*^	0.15	1.54	1.20^*^ (1.23^*^)	0.14	1.53	1.28^*^	0.14	1.53
	706	0.563	0.09^**^	2.45	1.39	0.09^**^ (0.09^**^)	2.45	1.40	0.10^**^	2.45	1.40

^*^ Rejection with respect to Pocock type stage level only;

^**^ Rejection with respect to both Pocock and O’Brien and Fleming type stage levels.

**Table 3.** Repeated 95%-confidence intervals based on Pocock type alpha spending function.

EDSS	N	Estimate	Brunner-Munzel		Brunner-Munzel (T)		Log win odds	
Month 24	353	0.545	0.479	0.610	0.479	0.610	0.478	0.609
	706	0.558	0.511	0.606	0.511	0.606	0.511	0.605
Change	353	0.564	0.499	0.628	0.499	0.628	0.499	0.626
	706	0.560	0.514	0.605	0.514	0.605	0.514	0.605
Direction	353	0.565	0.503	0.628	0.503	0.628	0.502	0.626
	706	0.563	0.519	0.608	0.519	0.608	0.518	0.607

**Table 4.** Repeated 95%-confidence intervals based on O’Brien and Fleming type alpha spending function.

EDSS	N	Estimate	Brunner-Munzel		Brunner-Munzel (T)		Log win odds	
Month 24	353	0.545	0.454	0.635	0.453	0.636	0.454	0.633
	706	0.558	0.516	0.601	0.516	0.601	0.516	0.600
Change	353	0.564	0.475	0.652	0.474	0.653	0.474	0.649
	706	0.560	0.519	0.601	0.519	0.601	0.518	0.600
Direction	353	0.565	0.479	0.651	0.478	0.652	0.478	0.649
	706	0.563	0.524	0.603	0.523	0.603	0.523	0.603

## 6 Planning and sample size considerations

In planning a clinical trial, a careful examination of the power of different scenarios under the alternative appears to be advisable at any rate. With the nonparametric relative effect p chosen as the efficacy estimand of the primary endpoint, we now extend and slightly modify the approach to sample size planning for the fixed scenario proposed by Happ et al.^34^ to the group sequential setting.

As before, we consider the hypothesis pair $H_{0}\;:\;p\leq 1/2$ and $H_{1}\;:\;p>1/2$ with a nominal overall significance level of α=0.025 . To determine the power of a particular alternative, it is convenient to specify the distributions $F_{1}$ and $F_{2}$ as well as a constant sample size ratio $t=n_{1k}/N_{k}$ for all stages k=1,…,K such that $F=tF_{1}+(1-t)F_{2}$ is the distribution of the whole data ignoring the group structure, which appears in the variance formula (4) of the Wilcoxon-Mann-Whitney test. If we then choose the sample sizes for the particular stages k=1,…,K , we immediately get the true information $I_{k}^{WMW}$ , $I_{k}^{BM}$ , $I_{k}^{LWO}$ as given in (3), (6) and (10), respectively. Approximate power formulas for the group sequential Wilcoxon-Mann-Whitney, Brunner-Munzel and log win odds tests then take the form as provided in the following two propositions.

Proposition 3 Let $c_{1},\ldots ,c_{K}$ denote the critical values computed from a K -variate normal distribution with mean vector 0 , covariance matrix $R^{WMW}=(r_{ij}{)}_{i,j=1,\ldots ,K}$ , $r_{ij}=\sqrt{I_{\min(k_{i},k_{\;j})}^{WMW}/I_{\max(k_{i},k_{\;j})}^{WMW}}$ , and error spending function of choice. Then the approximate power of the group sequential Wilcoxon-Mann-Whitney test for $H_{1}\;:\;p>1/2$ is given by

PowerWMW≈1−ΦR{I1BM/I1WMW⋅c1−I1BM⋅(p−1/2),…,xIKBM/IKWMW⋅cK−IKBM⋅(p−1/2)}

where $\Phi_{R}$ denotes the cumulative distribution function of a K -variate normal distribution with mean vector 0 and covariance matrix $R=(r_{ij})$ , $r_{ij}=\sqrt{N_{\min(k_{i},k_{\;j})}/N_{\max(k_{i},k_{\;j})}}$ .

Proposition 4 Let $c_{1},\ldots ,c_{K}$ denote the critical values computed from a K -variate normal distribution with mean vector 0 , covariance matrix $R=(r_{ij})$ , $r_{ij}=\sqrt{N_{\min(k_{i},k_{\;j})}/N_{\max(k_{i},k_{\;j})}}$ , and error spending function of choice. Then the approximate power of the group sequential Brunner-Munzel and log win odds tests for $H_{1}\;:\;p>1/2$ is given by

$$\begin{array}{rl} \mathrm{Powe}r_{\mathrm{BM}} & \approx 1-\Phi_{R}\left\{c_{1}-\sqrt{I_{1}^{BM}}\cdot \left(\;p-1/2\right)\;,\ldots ,c_{K}-\sqrt{I_{K}^{BM}}\cdot \left(\;p-1/2\right)\right\}\\ \mathrm{Powe}r_{\mathrm{LWO}} & \approx 1-\Phi_{R}\left(c_{1}-\sqrt{I_{1}^{LWO}}\cdot \psi \;,\ldots ,c_{K}-\sqrt{I_{K}^{LWO}}\cdot \psi \right),\;\psi =\ln\;\{p/(1-p)\} \end{array}$$

respectively, where $\Phi_{R}$ denotes the cumulative distribution function of a K -variate normal distribution with mean vector 0 and covariance matrix R as given above.

The critical values $c_{1},\ldots ,c_{K}$ as well as $\Phi_{R}(\cdot )$ can be easily obtained from the commands getDesignGroupSequential and pmvnorm of the respective R packages rpact^31^ and mvtnorm.^35^ To demonstrate the adequacy of the formulas just presented, the results of a small simulation study with 100,000 replications based on the ordinal distribution defined as in Section 4.2 are depicted in Table 5.

**Table 5.** Power of the Wilcoxon-Mann-Whitney (WMW), Brunner-Munzel (BM), and log win odds (LWO) tests for an equally spaced two stage trial with ordinal data as in Section 4.2, p=0.6 , $\alpha_{1}=0.6974797$ , $\beta_{1}=1$ , $\alpha_{2}=3$ , $\beta_{2}=3$ .

t	Test	Error spending function	$N_{1}$	$N_{2}$	Power formula	Simulated power (stage one)
0.5	WMW	Pocock	142	284	0.80382	0.80352 (0.48612)
0.5	BM	Pocock	144	288	0.80231	0.79546 (0.47652)
0.5	LWO	Pocock	152	304	0.80213	0.80372 (0.47272)
0.5	WMW	O’Brien and Fleming	126	252	0.80008	0.79989 (0.16823)
0.5	BM	O’Brien and Fleming	130	260	0.80597	0.79743 (0.19909)
0.5	LWO	O’Brien and Fleming	136	272	0.80232	0.80717 (0.12543)
2/3	WMW	Pocock	153	306	0.80488	0.80571 (0.46197)
2/3	BM	Pocock	132	264	0.80784	0.80016 (0.47790)
2/3	LWO	Pocock	138	276	0.80379	0.80569 (0.47236)
2/3	WMW	O’Brien and Fleming	135	270	0.80472	0.80364 (0.13013)
2/3	BM	O’Brien and Fleming	117	234	0.80417	0.79515 (0.19662)
2/3	LWO	O’Brien and Fleming	123	246	0.80242	0.80582 (0.12398)

## 7 Discussion

In this paper, we derived group sequential methodology for the Wilcoxon-Mann-Whitney, the Brunner-Munzel, and the log win odds tests, establishing their convergence in distribution to the canonical joint distribution, with simulation studies lending further support to the validity of our approach.

If one is willing both to assume the distributions to be equal under the null and to dispense with confidence intervals, the group sequential Wilcoxon-Mann-Whitney test best maintains the nominal significance level, particularly if sample sizes are small.

In the presence of heteroskedasticity, the Wilcoxon-Mann-Whitney test is either too liberal or too conservative depending on the heteroskedasticity pattern and the sample size allocation ratio. On the other hand, the log win odds test never exceeds the nominal significance level but does have a somewhat conservative tendency in certain scenarios. Nonetheless, the log win odds test allows for test inversion to compute confidence limits for the log win odds, which can readily be converted to the win odds or nonparametric relative effect scales. While the Brunner-Munzel test, with or without t -approximation, can be inverted in the same manner, it tends to be too liberal, especially in case of small sample sizes. In light of the fact that the Brunner-Munzel test gives rise to liberal test decisions for nominal significance levels smaller than 0.05 in the nonsequential setting in small samples, this result is hardly surprising.

In the randomised clinical trial setting, there appears little reason to conclude that distributions under the null are not identical. Still, if the treatment arms produce heteroskedastic outcomes in the alternative, one may well be led to infer from the simulation results that the Wilcoxon-Mann-Whitney test might actually turn out to be less powerful than the log win odds test in certain cases. However, as our case study in Section 5 suggests, the different behaviours of the tests are presumably negligible when sample sizes are reasonably large.

Care should be taken when adopting our methods for multi-arm trials. While Dunnet-type^36^ many-to-one comparisons should not pose particular difficulties, Tukey-type^37^ all-pairwise comparisons might lead to Efron’s paradox,^38–40^ that is, the nonparametric relative effect as defined in this paper may point to nontransitive conclusions. If treatment 1 is more beneficial than treatment 2 and treatment 2 is more beneficial than treatment 3, then it does not necessarily follow that treatment 1 is more beneficial than treatment 3.

Since the variance estimators require the endpoint at issue to induce a rank representation and therefore all pairwise comparisons to be transitive, the methodology presented here does not cover hierarchical composite and possibly censored endpoints in general terms as discussed in Buyse,^41^ Cantagallo et al.,^42^ Péron et al.,^43^ or Buyse and Péron.^44^ However, the idea of linking group sequential theory with generalised U -statistics^45,46^ might prove fruitful in extending our approach in this direction.

## Supplemental Material

sj-pdf-1-smm-10.1177_09622802221107103 - Supplemental material for Group sequential methods for the Mann-Whitney parameter Click here for additional data file. Supplemental material, sj-pdf-1-smm-10.1177_09622802221107103 for Group sequential methods for the Mann-Whitney parameter by Claus P Nowak, Tobias Mütze and Frank Konietschke in Statistical Methods in Medical Research

sj-R-2-smm-10.1177_09622802221107103 - Supplemental material for Group sequential methods for the Mann-Whitney parameter Click here for additional data file. Supplemental material, sj-R-2-smm-10.1177_09622802221107103 for Group sequential methods for the Mann-Whitney parameter by Claus P Nowak, Tobias Mütze and Frank Konietschke in Statistical Methods in Medical Research

sj-R-3-smm-10.1177_09622802221107103 - Supplemental material for Group sequential methods for the Mann-Whitney parameter Click here for additional data file. Supplemental material, sj-R-3-smm-10.1177_09622802221107103 for Group sequential methods for the Mann-Whitney parameter by Claus P Nowak, Tobias Mütze and Frank Konietschke in Statistical Methods in Medical Research

## References

[1] European Medicines Agency. *Reflection Paper on Methodological Issues in Confirmatory Clinical Trials Planned with an Adaptive Design*, 2007. https://www.ema.europa.eu/en/documents/scientific-guideline/reflection-.

[2] US Food and Drug Administration. *Adaptive Designs for Clinical Trials of Drugs and Biologics: Guidance for Industry*, 2019. https://www.fda.gov/media/78495/download (Accessed November 9, 2020).

[3] Jennison C Turnbull BW . Group Sequential Methods with Applications to Clinicial Trials. Boca Raton: Chapman & Hall/CRC, 2000.

[4] Proschan MA Lan KKG Wittes J . Statistical Monitoring of Clinical Trials: A Unified Approach. MA, New York: Springer, 2006.

[5] Wassmer G Brannath W . Group Sequential and Confirmatory Adaptive Designs in Clinical Trials. Springer International Publishing, 2016.

[6] Mann HB Whitney DR . On a test of whether one of two random variables is stochastically larger than the other. Ann Math Stat 1947; 18: 50–60.

[7] Wilcoxon F . Individual comparisons by ranking methods. Biometric Bull 1945; 1: 80–83.

[8] Wilcoxon F . Probability tables for individual comparisons by ranking methods. Biometrics 1947; 3: 119–122. 18903631

[9] Alling DW . Early decision in the Wilcoxon two-sample test. J Am Stat Assoc 1963; 58: 713–720.

[10] Phatarfod RM Sudbury A . A simple sequential Wilcoxon test. Aust J Stat 1988; 30: 93–106.

[11] Shuster JJ Chang MN Tian L . Design of group sequential clinical trials with ordinal categorical data based on the Mann–Whitney–Wilcoxon test. Seq Anal 2004; 23: 413–426.

[12] Brunner E Munzel U . The nonparametric Behrens-Fisher problem: Asymptotic theory and a small-sample approximation. Biom J 2000; 42: 17–25.

[13] Brunner E Bathke AC Konietschke F . Rank and Pseudo-Rank Procedures for Independent Observations in Factorial Designs. Springer International Publishing, 2018.

[14] Thas O De Neve J Clement L et al. Probabilistic index models. J R Stat Soc B (Statistical Methodology) 2012; 74: 623–671.

[15] Fay MP Brittain EH Shih JH et al. Causal estimands and confidence intervals associated with Wilcoxon-Mann-Whitney tests in randomized experiments. Stat Med 2018; 37: 2923–2937. 29774591 10.1002/sim.7799 PMC6373726

[16] Brunner E Vandemeulebroecke M Mütze T . Win odds: An adaptation of the win ratio to include ties. Stat Med 2021; 40: 3367–3384. 33860957 10.1002/sim.8967

[17] Putter J . The treatment of ties in some nonparametric tests. Ann Math Stat 1955; 26: 368–386.

[18] Pocock SJ Ariti CA Collier TJ et al. The win ratio: A new approach to the analysis of composite endpoints in clinical trials based on clinical priorities. Eur Heart J 2011; 33: 176–182. 21900289 10.1093/eurheartj/ehr352

[19] Wang D Pocock S . A win ratio approach to comparing continuous non-normal outcomes in clinical trials. Pharm Stat 2016; 15: 238–245. 26970432 10.1002/pst.1743

[20] Gasparyan SB Folkvaljon F Bengtsson O et al. Adjusted win ratio with stratification: Calculation methods and interpretation. Stat Methods Med Res 2020; 0: 1–32. 10.1177/0962280220942558 32726191

[21] Scharfstein DO Tsiatis AA Robins JM . Semiparametric efficiency and its implication on the design and analysis of group-sequential studies. J Am Stat Assoc 1997; 92: 1342–1350.

[22] Cramér H Wold H . Some theorems on distribution functions. J Lond Math Soc 1936; s1-11: 290–294.

[23] Lévy P . Calcul des probabilités, volume 9. Paris: Gauthier-Villars Paris, 1925.

[24] Ruymgaart FH (1980) A unified approach to the asymptotic distribution theory of certain midrank statistics. In Raoult JP (eds.) Statistique non Paramétrique Asymptotique. Lecture Notes in Mathematics, Vol 821. Springer, Berlin: Heidelberg. 10.1007/BFb0097422

[25] Satterthwaite FE . An approximate distribution of estimates of variance components. Biometrics Bull 1946; 2: 110–114. 20287815

[26] Smith HF . The problem of comparing the results of two experiments with unequal errors. J Council Sci Ind Res 1936; 9: 211–212.

[27] Welch BL . The significance of the difference between two means when the population variances are unequal. Biometrika 1937; 29: 350–362.

[28] Pocock SJ . Group sequential methods in the design and analysis of clinical trials. Biometrika 1977; 64: 191–199.

[29] O’Brien PC Fleming TR . A multiple testing procedure for clinical trials. Biometrics 1979; 35: 549–556. 497341

[30] Lan KKG DeMets DL . Discrete sequential boundaries for clinical trials. Biometrika 1983; 70: 659–663.

[31] Wassmer G Pahlke F . *rpact: Confirmatory Adaptive Clinical Trial Design and Analysis*, 2020. https://CRAN.R-project.org/package=rpact. R package version 3.0.1.

[32] Kappos L Radue EW O’Connor P et al. A placebo-controlled trial of oral fingolimod in relapsing multiple sclerosis. N Engl J Med 2010; 362: 387–401. 20089952 10.1056/NEJMoa0909494

[33] Kurtzke JF . Rating neurologic impairment in mulitple sclerosis: An expanded disability status scale (EDSS). Neurology 1983; 33: 1444–1452. 6685237 10.1212/wnl.33.11.1444

[34] Happ M Bathke AC Brunner E . Optimal sample size planning for the Wilcoxon-Mann-Whitney test. Stat Med 2019; 38: 363–375. 30298671 10.1002/sim.7983 PMC6491996

[35] Genz A Bretz F Miwa T et al. *mvtnorm: Multivariate Normal and t Distributions*, 2020. https://CRAN.R-project.org/package=mvtnorm. R package version 1.1-1.

[36] Dunnett CW . A multiple comparison procedure for comparing several treatments with a control. J Am Stat Assoc 1955; 50: 1096–1121.

[37] Tukey J . Comparing individual means in the analysis of variance. Biometrics 1949; 5: 99–114. 18151955

[38] Gardner M . The paradox of the nontransitive dice and the elusive principle of indifference. Sci Am: Math Games Column 1970; 223: 110–114.

[39] Savage RP . The paradox of nontransitive dice. Am Math Mon 1994; 101: 429–436.

[40] Thangevelu K Brunner E . Wilcoxon-Mann-Whitney test for stratified samples and Efron’s paradox dice. J Stat Plan Inference 2007; 137: 720–737.

[41] Buyse M . Generalized pairwise comparisons of prioritized outcomes in the two-sample problem. Stat Med 2010; 29: 3245–3257. 21170918 10.1002/sim.3923

[42] Cantagallo E De Backer M Kicinski M et al. A new measure of treatment effect in clinical trials involving competing risks based on generalized pairwise comparisons. Biom J 2021; 63: 272–288. 32939818 10.1002/bimj.201900354

[43] Péron J Buyse M Ozenne B et al. An extension of generalized pairwise comparisons for prioritized outcomes in the presence of censoring. Stat Methods Med Res 2018; 27: 1230–1239. 27487842 10.1177/0962280216658320

[44] Buyse M Peron J . Generalized pairwise comparisons for prioritized outcomes. In Piantadosi S and Meinert CL (eds.) *Principles and Practice of Clinical Trials*. Cham: Springer, 2020. pp. 1–25.

[45] Hoeffding W . A class of statistics with asymptotically normal distributions. Ann Stat 1948; 19: 293–325.

[46] Lee AJ . U-Statistics: Theory and Practice. New York: Marcel Dekker, 1990.

