# Supplementary material for: Group sequential methods for the Mann-Whitney parameter
Source: Stat Methods Med Res. 2022 Jun 13;31(10):2004–20. doi: 10.1177/09622802221107103 (PMC9523827; doi:10.1177/09622802221107103)
Supplement: sj-pdf-1-smm-10.1177_09622802221107103 - Supplemental material for Group sequential methods for the Mann-Whitney parameter [file sj-pdf-1-smm-10.1177_09622802221107103.pdf]

Group sequential methods for the Mann-Whitney parameter

Supplementary material

Claus P. Nowak[1,2], Tobias Mütze[3], and Frank Konietschke[1,*]

[1]Charité – Universitätsmedizin Berlin,

corporate member of Freie Universität Berlin and Humboldt-Universität zu Berlin,

Institute of Biometry and Clinical Epidemiology, Charitéplatz 1, 10117 Berlin, Germany

[2]TU Dortmund University, Faculty of Statistics, Dortmund, Germany

[3]Statistical Methodology, Novartis Pharma AG, Basel, Switzerland

[*]*email:* frank.konietschke@charite.de

Contents

This supplementary material provides more detail on the methodology and the results of the simulations. First, we provide summary figures for rejection rates with ordinal data. Then we discuss exception handling as regards variance estimation in extreme cases together with the algorithm computing the stage levels. Afterwards, we present tables and graphs depicting cumulative rejection rates at each of the stages.

Ordinal data

We now give overall rejection rates for the data generated by a latent beta distribution as discussed in Section 4.2 of the main manuscript. We specify three different parameter settings as given in Figures 1 to 3. The nonparametric relative effects in the last two settings equal 0.5 as well since identical shape parameters give rise to a symmetric Beta distribution with expectation 0.5, the variance being larger with both shape parameters set to 1.

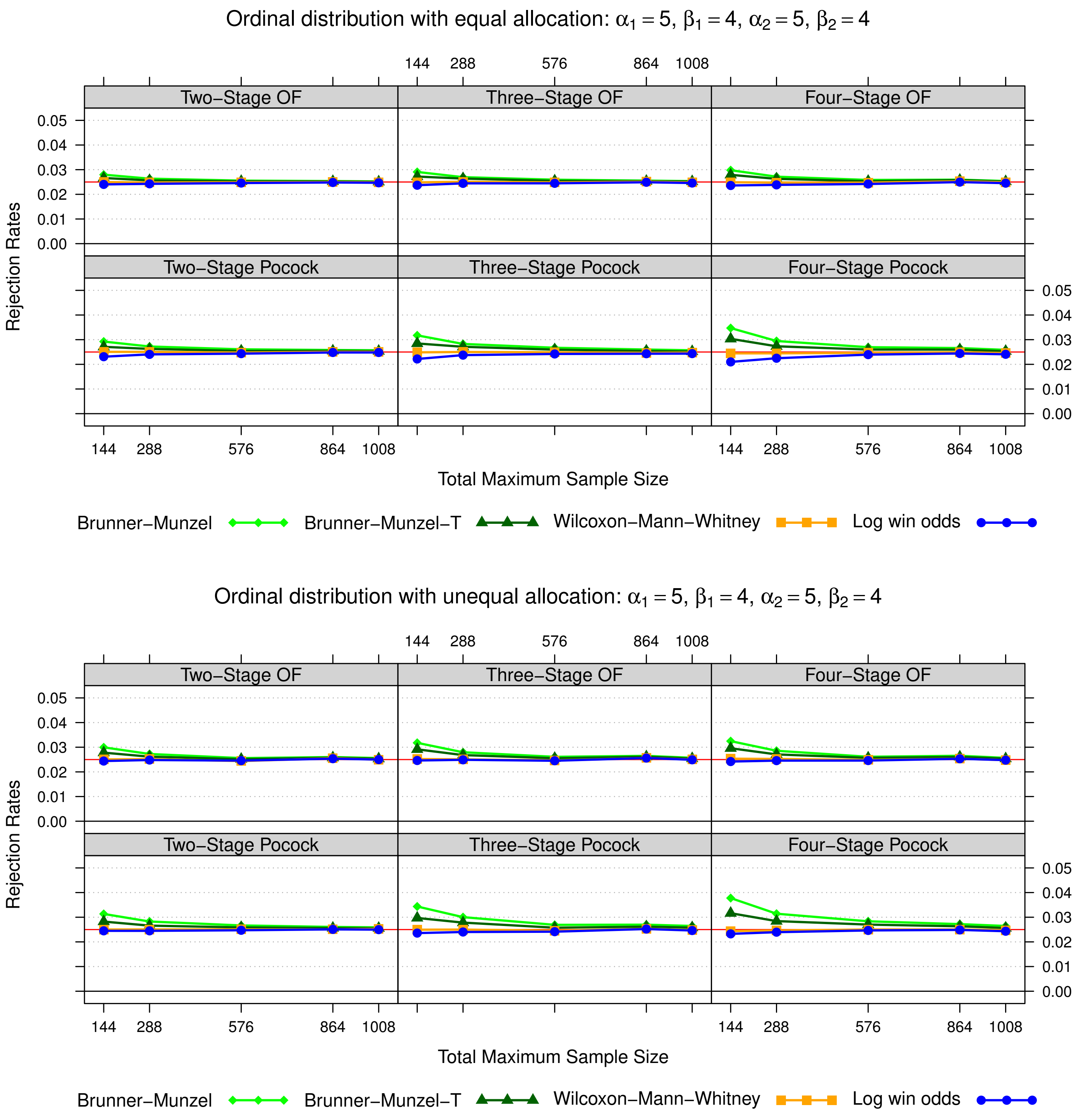

Figure 1: Ordinal distribution - Setting 1

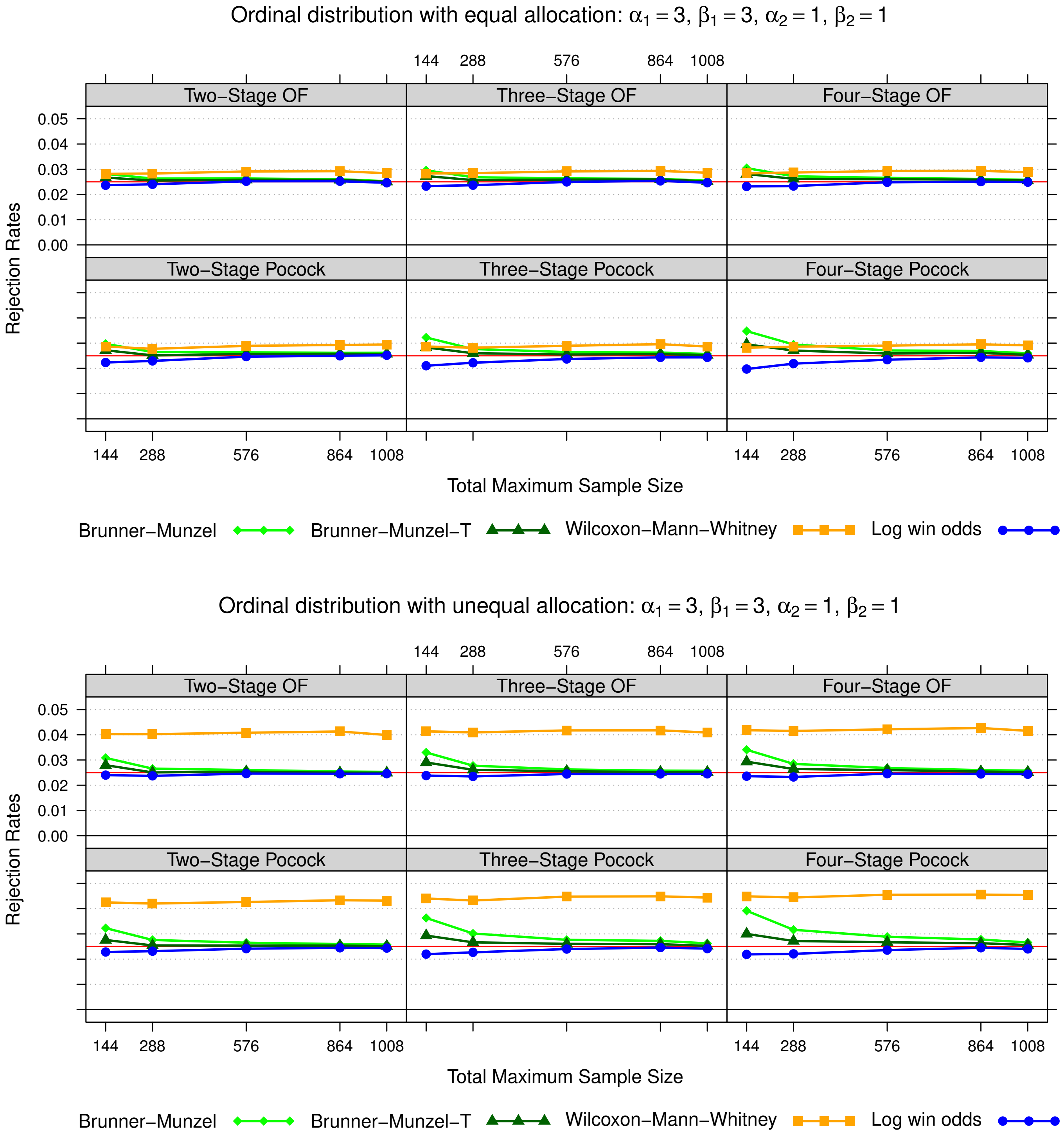

Figure 2: Ordinal distribution - Setting 2

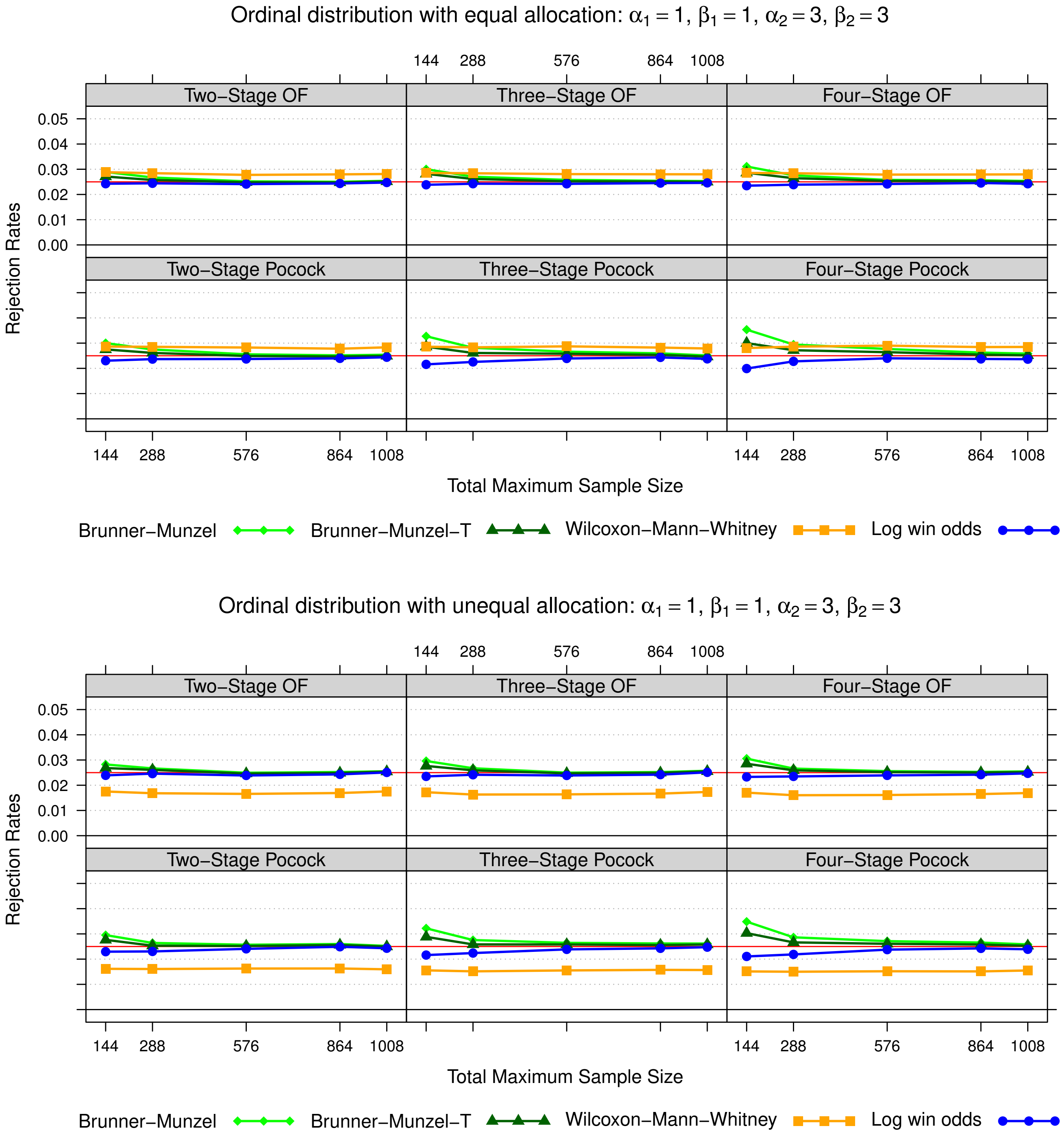

Figure 3: Ordinal distribution - Setting 3

Exception handling

A sample in which all responses for patients on both treatment arms are equal at stage $k = 1, \ldots, K$,

$$x_{11} = \cdots = x_{1n_{1k}} = x_{21} = \cdots = x_{2n_{2k}},$$

produces $\widehat{\sigma}^2_{Rk} = \widehat{\sigma}^2_{1k} = \widehat{\sigma}^2_{2k} = 0$. Thus, the information estimates for the Wilcoxon-Mann-Whitney test $\widehat{\mathcal{I}}^{WMW}_k$, the Brunner-Munzel test $\widehat{\mathcal{I}}^{BM}_k$, and the log win odds test $\widehat{\mathcal{I}}^{LWO}_k = \{\widehat{p}^{(k)}(1-\widehat{p}^{(k)})\}^2 \widehat{\mathcal{I}}^{BM}_k$, $k = 1, \ldots, K$, are not defined.

Without changing the effect estimate of p, we assumed without loss of generality that the last observation was different,

$$x_{11} = \cdots = x_{1n_{1k}} = x_{21} = \cdots \neq x_{2n_{2k}},$$

yielding $\widehat{\sigma}^2_{Rk} = N_k/4$. Thus we set $\widehat{\mathcal{I}}^{WMW}_k = \widehat{\mathcal{I}}^{BM}_k = 4n_{1k}n_{2k}$. As far as the t-approximation for the Brunner-Munzel test is concerned, setting $\widehat{\sigma}^2_{1k} = \widehat{\sigma}^2_{2k} > 0$ gives

$$\widehat{\nu}_k = \{N_k^2(n_{1k}-1)(n_{2k}-1)\}/\{n_{1k}^2(n_{1k}-1) + n_{2k}^2(n_{2k}-1)\}.$$

A sample which is completely separated at stage $k = 1, \ldots, K$, such as

$$x_{11} < \cdots < x_{1n_{1k}} < x_{21} < \cdots < x_{2n_{2k}},$$

or

$$x_{11} > \cdots > x_{1n_{1k}} > x_{21} > \cdots > x_{2n_{2k}},$$

results in $\widehat{\sigma}^2_{Rk} > 0$. However, we then have $\widehat{\sigma}^2_{1k} = \widehat{\sigma}^2_{2k} = 0$ and the estimates $\widehat{\mathcal{I}}^{BM}_k$ and $\widehat{\mathcal{I}}^{LWO}_k$ are undefined.

Proceeding similarly as before, we then assume our sample were not completely separated, with the largest value of one treatment arm coinciding with the lowest value of the other,

$$x_{11} < \cdots < x_{1n_{1k}} = x_{21} < \cdots < x_{2n_{2k}},$$

or

$$x_{11} > \cdots > x_{1n_{1k}} = x_{21} > \cdots > x_{2n_{2k}},$$

yielding a slight change in the effect estimate $\widehat{p}^{(k)} = 1 - 1/(2n_{1k}n_{2k})$ or $\widehat{p}^{(k)} = 1/(2n_{1k}n_{2k})$ respectively. In this changed scenario the information estimates equal $\widehat{\mathcal{I}}^{BM}_k = 2n_{1k}^2 n_{2k}^2$ and we consequently base $\widehat{\mathcal{I}}^{LWO}_k = \{\widehat{p}^{(k)}(1-\widehat{p}^{(k)})\}^2 \widehat{\mathcal{I}}^{BM}_k$ on the slightly altered effect estimates as well.

This approach is in line with suggestions from Brunner et al. (2018). Nevertheless, it remains questionable under such circumstances whether formal hypothesis tests are desirable at all. Moreover, it still happened from time to time that the algorithm we used to determine the stage levels, i.e. the command

```
getDesignGroupSequential()
```

from the R package `rpact` (Wassmer and Pahlke, 2020) broke down, producing one of the following two error messages,

- NA returned because root search via 'bisection' produced a function result (0.0827806785655401) that differs from target 0 (tolerance is 1e-08, last function argument was 7.99999999254942),
- Runtime exception: in .getGroupSequentialProbabilities(): variable 'dn' contains 91 NA's (100.0%).

These failures only occurred with respect to the Brunner-Munzel test and almost exclusively if the total maximum sample size was 144, particularly in case of three interim analyses. The sole exception was one of the normal heteroskedastic settings with three interim analyses, unequal allocation and total maximum sample size 288 (see Table 7). However, we felt that tinkering further with these issues, resolving them one way or another, would not provide any additional insights, not least because we think it highly unlikely that anyone would wish to perform three interim analyses in a real clinical trial with total maximum sample size 144. Thus, we simply went on to discard the cases in which the simulation runs failed and based the calculation of the rejection rates on the remaining ones.

More precisely, row 6 of Table 1 shows that the command `getDesignGroupSequential()` failed in 15 out of 100000 simulation runs, producing a rejection rate of 0.014882232 in the given setting. This means that out of 99985 successful simulation runs the null hypothesis could be rejected

$$0.014882232 \cdot (100000 - 15) = 0.014882232 \cdot 99985 = 1488$$

times.

Tables and Graphs

The following 35 tables all share the same structure, N_K denotes the total maximum sample size, AR the allocation ratio, K the total maximum number of stages, N_k the cumulative total sample size available at that particular stage, and f is the error spending function. Moreover, `NA` depicts the number of times when `getDesignGroupSequential()` failed to compute the stage levels for the respective tests, whereas WMW, BM, BM-T, and LWO list the cumulative rejection rates for the respective tests at the respective stages.

In addition to the data generating processes set out in the main manuscript, we also consider the case where responses from both treatment groups follow a Poisson distribution with rate parameters $\lambda_1 = \lambda_2 = 2$.

Moreover, Figures 4-38 provide a graphical representation of the respective tables.

List of Tables

1 Normal distribution with $\mu_1 = 0, \mu_2 = 0, \sigma_1 = 1, \sigma_2 = 1$ 9
2 Normal distribution with $\mu_1 = 0, \mu_2 = 0, \sigma_1 = 1, \sigma_2 = 1$ 10
3 Normal distribution with $\mu_1 = 0, \mu_2 = 0, \sigma_1 = 1, \sigma_2 = 1$ 11
4 Normal distribution with $\mu_1 = 0, \mu_2 = 0, \sigma_1 = 1, \sigma_2 = 1$ 12
5 Normal distribution with $\mu_1 = 0, \mu_2 = 0, \sigma_1 = 1, \sigma_2 = 1$ 13
6 Normal distribution with $\mu_1 = 0, \mu_2 = 0, \sigma_1 = 1, \sigma_2 = 2$ 14
7 Normal distribution with $\mu_1 = 0, \mu_2 = 0, \sigma_1 = 1, \sigma_2 = 2$ 15
8 Normal distribution with $\mu_1 = 0, \mu_2 = 0, \sigma_1 = 1, \sigma_2 = 2$ 16
9 Normal distribution with $\mu_1 = 0, \mu_2 = 0, \sigma_1 = 1, \sigma_2 = 2$ 17
10 Normal distribution with $\mu_1 = 0, \mu_2 = 0, \sigma_1 = 1, \sigma_2 = 2$ 18
11 Normal distribution with $\mu_1 = 0, \mu_2 = 0, \sigma_1 = 2, \sigma_2 = 1$ 19
12 Normal distribution with $\mu_1 = 0, \mu_2 = 0, \sigma_1 = 2, \sigma_2 = 1$ 20
13 Normal distribution with $\mu_1 = 0, \mu_2 = 0, \sigma_1 = 2, \sigma_2 = 1$ 21
14 Normal distribution with $\mu_1 = 0, \mu_2 = 0, \sigma_1 = 2, \sigma_2 = 1$ 22
15 Normal distribution with $\mu_1 = 0, \mu_2 = 0, \sigma_1 = 2, \sigma_2 = 1$ 23
16 Ordinal distribution with $\alpha_1 = 5, \beta_1 = 4, \alpha_2 = 5, \beta_2 = 4$ 24
17 Ordinal distribution with $\alpha_1 = 5, \beta_1 = 4, \alpha_2 = 5, \beta_2 = 4$ 25
18 Ordinal distribution with $\alpha_1 = 5, \beta_1 = 4, \alpha_2 = 5, \beta_2 = 4$ 26
19 Ordinal distribution with $\alpha_1 = 5, \beta_1 = 4, \alpha_2 = 5, \beta_2 = 4$ 27
20 Ordinal distribution with $\alpha_1 = 5, \beta_1 = 4, \alpha_2 = 5, \beta_2 = 4$ 28
21 Ordinal distribution with $\alpha_1 = 3, \beta_1 = 3, \alpha_2 = 1, \beta_2 = 1$ 29
22 Ordinal distribution with $\alpha_1 = 3, \beta_1 = 3, \alpha_2 = 1, \beta_2 = 1$ 30
23 Ordinal distribution with $\alpha_1 = 3, \beta_1 = 3, \alpha_2 = 1, \beta_2 = 1$ 31
24 Ordinal distribution with $\alpha_1 = 3, \beta_1 = 3, \alpha_2 = 1, \beta_2 = 1$ 32
25 Ordinal distribution with $\alpha_1 = 3, \beta_1 = 3, \alpha_2 = 1, \beta_2 = 1$ 33
26 Ordinal distribution with $\alpha_1 = 1, \beta_1 = 1, \alpha_2 = 3, \beta_2 = 3$ 34
27 Ordinal distribution with $\alpha_1 = 1, \beta_1 = 1, \alpha_2 = 3, \beta_2 = 3$ 35
28 Ordinal distribution with $\alpha_1 = 1, \beta_1 = 1, \alpha_2 = 3, \beta_2 = 3$ 36
29 Ordinal distribution with $\alpha_1 = 1, \beta_1 = 1, \alpha_2 = 3, \beta_2 = 3$ 37
30 Ordinal distribution with $\alpha_1 = 1, \beta_1 = 1, \alpha_2 = 3, \beta_2 = 3$ 38
31 Poisson distribution with $\lambda_1 = 2, \lambda_2 = 2$ 39
32 Poisson distribution with $\lambda_1 = 2, \lambda_2 = 2$ 40
33 Poisson distribution with $\lambda_1 = 2, \lambda_2 = 2$ 41
34 Poisson distribution with $\lambda_1 = 2, \lambda_2 = 2$ 42
35 Poisson distribution with $\lambda_1 = 2, \lambda_2 = 2$ 43

List of Figures

4 Normal distribution - Setting 1 with total maximum sample size 144 44

5 Normal distribution - Setting 1 with total maximum sample size 288 45

6 Normal distribution - Setting 1 with total maximum sample size 576 46

7 Normal distribution - Setting 1 with total maximum sample size 864 47

8 Normal distribution - Setting 1 with total maximum sample size 1008 48

9 Normal distribution - Setting 2 with total maximum sample size 144 49

10 Normal distribution - Setting 2 with total maximum sample size 288 50

11 Normal distribution - Setting 2 with total maximum sample size 576 51

12 Normal distribution - Setting 2 with total maximum sample size 864 52

13 Normal distribution - Setting 2 with total maximum sample size 1008 53

14 Normal distribution - Setting 3 with total maximum sample size 144 54

15 Normal distribution - Setting 3 with total maximum sample size 288 55

16 Normal distribution - Setting 3 with total maximum sample size 576 56

17 Normal distribution - Setting 3 with total maximum sample size 864 57

18 Normal distribution - Setting 3 with total maximum sample size 1008 58

19 Ordinal distribution - Setting 1 with total maximum sample size 144 59

20 Ordinal distribution - Setting 1 with total maximum sample size 288 60

21 Ordinal distribution - Setting 1 with total maximum sample size 576 61

22 Ordinal distribution - Setting 1 with total maximum sample size 864 62

23 Ordinal distribution - Setting 1 with total maximum sample size 1008 63

24 Ordinal distribution - Setting 2 with total maximum sample size 144 64

25 Ordinal distribution - Setting 2 with total maximum sample size 288 65

26 Ordinal distribution - Setting 2 with total maximum sample size 576 66

27 Ordinal distribution - Setting 2 with total maximum sample size 864 67

28 Ordinal distribution - Setting 2 with total maximum sample size 1008 68

29 Ordinal distribution - Setting 3 with total maximum sample size 144 69

30 Ordinal distribution - Setting 3 with total maximum sample size 288 70

31 Ordinal distribution - Setting 3 with total maximum sample size 576 71

32 Ordinal distribution - Setting 3 with total maximum sample size 864 72

33 Ordinal distribution - Setting 3 with total maximum sample size 1008 73

34 Poisson distribution with total maximum sample size 144 74

35 Poisson distribution with total maximum sample size 288 75

36 Poisson distribution with total maximum sample size 576 76

37 Poisson distribution with total maximum sample size 864 77

38 Poisson distribution with total maximum sample size 1008 78

Table 1: Normal distribution with $\mu_1 = 0, \mu_2 = 0, \sigma_1 = 1, \sigma_2 = 1$

N_K	AR	K	N_k	f	NA	WMW	NA	BM	BM-T	NA	LWO
144	1:1	2	72	asP	0	0.01590	0	0.019540000	0.017860000	0	0.01232
144	1:1	2	144	asP	0	0.02581	0	0.030030000	0.027920000	0	0.02250
144	1:1	3	48	asP	0	0.01119	1	0.016600166	0.013980140	0	0.00699
144	1:1	3	96	asP	0	0.01906	1	0.025530255	0.022530225	0	0.01427
144	1:1	3	144	asP	0	0.02512	1	0.032020320	0.028560286	0	0.02069
144	1:1	4	36	asP	0	0.00769	15	0.014882232	0.011861779	0	0.00371
144	1:1	4	72	asP	0	0.01466	15	0.023273491	0.019682952	0	0.00957
144	1:1	4	108	asP	0	0.01995	15	0.029154373	0.025283793	0	0.01461
144	1:1	4	144	asP	0	0.02461	15	0.033655048	0.029784468	0	0.01961
144	1:1	2	72	asOF	0	0.00127	0	0.004590000	0.003820000	0	0.00012
144	1:1	2	144	asOF	0	0.02516	0	0.028100000	0.026810000	0	0.02373
144	1:1	3	48	asOF	0	0.00001	1	0.002020020	0.001420014	0	0.00000
144	1:1	3	96	asOF	0	0.00614	1	0.011060111	0.009710097	0	0.00285
144	1:1	3	144	asOF	0	0.02543	1	0.029460295	0.027800278	0	0.02325
144	1:1	4	36	asOF	0	0.00000	15	0.001380207	0.000600090	0	0.00000
144	1:1	4	72	asOF	0	0.00127	15	0.005580837	0.004190629	0	0.00012
144	1:1	4	108	asOF	0	0.00966	15	0.015132270	0.013392009	0	0.00554
144	1:1	4	144	asOF	0	0.02538	15	0.029964495	0.027944192	0	0.02300
144	2:1	2	72	asP	0	0.01501	0	0.020310000	0.017470000	0	0.01239
144	2:1	2	144	asP	0	0.02491	0	0.031130000	0.027550000	0	0.02233
144	2:1	3	48	asP	0	0.01022	8	0.017581407	0.014361149	0	0.00749
144	2:1	3	96	asP	0	0.01785	8	0.026722138	0.022831827	0	0.01457
144	2:1	3	144	asP	0	0.02400	8	0.033282663	0.028852308	0	0.02101
144	2:1	4	36	asP	0	0.00853	91	0.018096468	0.013592369	0	0.00471
144	2:1	4	72	asP	0	0.01495	91	0.026854438	0.021439510	0	0.01062
144	2:1	4	108	asP	0	0.02006	91	0.032919957	0.026964538	0	0.01527
144	2:1	4	144	asP	0	0.02446	91	0.037584202	0.031238427	0	0.02002
144	2:1	2	72	asOF	0	0.00140	0	0.005660000	0.004580000	0	0.00035
144	2:1	2	144	asOF	0	0.02510	0	0.029280000	0.027430000	0	0.02387
144	2:1	3	48	asOF	0	0.00005	7	0.002930205	0.002020141	0	0.00000
144	2:1	3	96	asOF	0	0.00579	7	0.012420869	0.010620743	0	0.00344
144	2:1	3	144	asOF	0	0.02512	7	0.030932165	0.028301981	0	0.02366
144	2:1	4	36	asOF	0	0.00000	81	0.002201783	0.001220989	0	0.00000
144	2:1	4	72	asOF	0	0.00130	81	0.007005675	0.005274272	0	0.00035
144	2:1	4	108	asOF	0	0.00893	81	0.017564227	0.015022168	0	0.00575
144	2:1	4	144	asOF	0	0.02514	81	0.032306168	0.029353777	0	0.02318

Table 2: Normal distribution with $\mu_1 = 0, \mu_2 = 0, \sigma_1 = 1, \sigma_2 = 1$

N_K	AR	K	N_k	f	NA	WMW	NA	BM	BM-T	NA	LWO
288	1:1	2	144	asP	0	0.01533	0	0.01730	0.01634	0	0.01358
288	1:1	2	288	asP	0	0.02475	0	0.02702	0.02576	0	0.02309
288	1:1	3	96	asP	0	0.01131	0	0.01424	0.01290	0	0.00922
288	1:1	3	192	asP	0	0.01933	0	0.02279	0.02117	0	0.01675
288	1:1	3	288	asP	0	0.02510	0	0.02864	0.02695	0	0.02262
288	1:1	4	72	asP	0	0.00885	0	0.01194	0.01066	0	0.00640
288	1:1	4	144	asP	0	0.01542	0	0.01936	0.01778	0	0.01256
288	1:1	4	216	asP	0	0.02109	0	0.02550	0.02378	0	0.01776
288	1:1	4	288	asP	0	0.02516	0	0.02960	0.02781	0	0.02206
288	1:1	2	144	asOF	0	0.00141	0	0.00279	0.00249	0	0.00066
288	1:1	2	288	asOF	0	0.02466	0	0.02599	0.02544	0	0.02384
288	1:1	3	96	asOF	0	0.00007	0	0.00050	0.00036	0	0.00002
288	1:1	3	192	asOF	0	0.00569	0	0.00810	0.00751	0	0.00415
288	1:1	3	288	asOF	0	0.02462	0	0.02642	0.02565	0	0.02358
288	1:1	4	72	asOF	0	0.00001	0	0.00037	0.00025	0	0.00000
288	1:1	4	144	asOF	0	0.00138	0	0.00308	0.00267	0	0.00066
288	1:1	4	216	asOF	0	0.00984	0	0.01270	0.01209	0	0.00737
288	1:1	4	288	asOF	0	0.02506	0	0.02723	0.02653	0	0.02372
288	2:1	2	144	asP	0	0.01518	0	0.01765	0.01651	0	0.01360
288	2:1	2	288	asP	0	0.02462	0	0.02750	0.02607	0	0.02337
288	2:1	3	96	asP	0	0.01104	0	0.01441	0.01281	0	0.00933
288	2:1	3	192	asP	0	0.01889	0	0.02280	0.02080	0	0.01675
288	2:1	3	288	asP	0	0.02484	0	0.02894	0.02679	0	0.02287
288	2:1	4	72	asP	0	0.00866	0	0.01345	0.01130	0	0.00692
288	2:1	4	144	asP	0	0.01515	0	0.02080	0.01826	0	0.01293
288	2:1	4	216	asP	0	0.02075	0	0.02669	0.02397	0	0.01834
288	2:1	4	288	asP	0	0.02508	0	0.03111	0.02820	0	0.02281
288	2:1	2	144	asOF	0	0.00135	0	0.00335	0.00297	0	0.00080
288	2:1	2	288	asOF	0	0.02460	0	0.02656	0.02565	0	0.02375
288	2:1	3	96	asOF	0	0.00010	0	0.00106	0.00077	0	0.00001
288	2:1	3	192	asOF	0	0.00585	0	0.00871	0.00804	0	0.00445
288	2:1	3	288	asOF	0	0.02491	0	0.02725	0.02630	0	0.02382
288	2:1	4	72	asOF	0	0.00000	0	0.00063	0.00048	0	0.00000
288	2:1	4	144	asOF	0	0.00135	0	0.00372	0.00324	0	0.00080
288	2:1	4	216	asOF	0	0.00952	0	0.01322	0.01214	0	0.00784
288	2:1	4	288	asOF	0	0.02481	0	0.02802	0.02676	0	0.02370

Table 3: Normal distribution with $\mu_1 = 0, \mu_2 = 0, \sigma_1 = 1, \sigma_2 = 1$

N_K	AR	K	N_k	f	NA	WMW	NA	BM	BM-T	NA	LWO
576	1:1	2	288	asP	0	0.01610	0	0.01706	0.01664	0	0.01521
576	1:1	2	576	asP	0	0.02577	0	0.02691	0.02644	0	0.02496
576	1:1	3	192	asP	0	0.01147	0	0.01274	0.01216	0	0.01036
576	1:1	3	384	asP	0	0.01925	0	0.02088	0.02010	0	0.01798
576	1:1	3	576	asP	0	0.02527	0	0.02698	0.02613	0	0.02407
576	1:1	4	144	asP	0	0.00908	0	0.01064	0.01011	0	0.00787
576	1:1	4	288	asP	0	0.01600	0	0.01799	0.01730	0	0.01440
576	1:1	4	432	asP	0	0.02133	0	0.02337	0.02264	0	0.01961
576	1:1	4	576	asP	0	0.02555	0	0.02762	0.02682	0	0.02398
576	1:1	2	288	asOF	0	0.00150	0	0.00208	0.00199	0	0.00105
576	1:1	2	576	asOF	0	0.02556	0	0.02608	0.02587	0	0.02508
576	1:1	3	192	asOF	0	0.00004	0	0.00029	0.00022	0	0.00001
576	1:1	3	384	asOF	0	0.00589	0	0.00703	0.00673	0	0.00500
576	1:1	3	576	asOF	0	0.02546	0	0.02630	0.02592	0	0.02486
576	1:1	4	144	asOF	0	0.00000	0	0.00007	0.00005	0	0.00000
576	1:1	4	288	asOF	0	0.00150	0	0.00212	0.00201	0	0.00105
576	1:1	4	432	asOF	0	0.00999	0	0.01111	0.01077	0	0.00872
576	1:1	4	576	asOF	0	0.02548	0	0.02639	0.02604	0	0.02477
576	2:1	2	288	asP	0	0.01624	0	0.01748	0.01666	0	0.01547
576	2:1	2	576	asP	0	0.02559	0	0.02700	0.02614	0	0.02497
576	2:1	3	192	asP	0	0.01154	0	0.01292	0.01219	0	0.01061
576	2:1	3	384	asP	0	0.01951	0	0.02147	0.02054	0	0.01842
576	2:1	3	576	asP	0	0.02541	0	0.02749	0.02650	0	0.02458
576	2:1	4	144	asP	0	0.00890	0	0.01124	0.01003	0	0.00782
576	2:1	4	288	asP	0	0.01596	0	0.01906	0.01755	0	0.01472
576	2:1	4	432	asP	0	0.02146	0	0.02477	0.02316	0	0.02027
576	2:1	4	576	asP	0	0.02583	0	0.02909	0.02744	0	0.02477
576	2:1	2	288	asOF	0	0.00169	0	0.00253	0.00232	0	0.00129
576	2:1	2	576	asOF	0	0.02554	0	0.02667	0.02618	0	0.02511
576	2:1	3	192	asOF	0	0.00009	0	0.00046	0.00037	0	0.00003
576	2:1	3	384	asOF	0	0.00617	0	0.00752	0.00715	0	0.00566
576	2:1	3	576	asOF	0	0.02532	0	0.02639	0.02581	0	0.02481
576	2:1	4	144	asOF	0	0.00000	0	0.00013	0.00007	0	0.00000
576	2:1	4	288	asOF	0	0.00169	0	0.00263	0.00236	0	0.00129
576	2:1	4	432	asOF	0	0.01037	0	0.01208	0.01159	0	0.00930
576	2:1	4	576	asOF	0	0.02557	0	0.02686	0.02625	0	0.02476

Table 4: Normal distribution with $\mu_1 = 0, \mu_2 = 0, \sigma_1 = 1, \sigma_2 = 1$

N_K	AR	K	N_k	f	NA	WMW	NA	BM	BM-T	NA	LWO
864	1:1	2	432	asP	0	0.01610	0	0.01692	0.01661	0	0.01553
864	1:1	2	864	asP	0	0.02568	0	0.02652	0.02618	0	0.02512
864	1:1	3	288	asP	0	0.01148	0	0.01234	0.01196	0	0.01074
864	1:1	3	576	asP	0	0.01909	0	0.02024	0.01970	0	0.01827
864	1:1	3	864	asP	0	0.02526	0	0.02641	0.02591	0	0.02446
864	1:1	4	216	asP	0	0.00855	0	0.00956	0.00912	0	0.00772
864	1:1	4	432	asP	0	0.01501	0	0.01634	0.01579	0	0.01420
864	1:1	4	648	asP	0	0.02013	0	0.02163	0.02103	0	0.01923
864	1:1	4	864	asP	0	0.02445	0	0.02603	0.02538	0	0.02361
864	1:1	2	432	asOF	0	0.00143	0	0.00185	0.00179	0	0.00106
864	1:1	2	864	asOF	0	0.02483	0	0.02520	0.02506	0	0.02450
864	1:1	3	288	asOF	0	0.00010	0	0.00021	0.00018	0	0.00006
864	1:1	3	576	asOF	0	0.00612	0	0.00664	0.00649	0	0.00544
864	1:1	3	864	asOF	0	0.02504	0	0.02556	0.02531	0	0.02458
864	1:1	4	216	asOF	0	0.00001	0	0.00006	0.00004	0	0.00000
864	1:1	4	432	asOF	0	0.00143	0	0.00187	0.00181	0	0.00106
864	1:1	4	648	asOF	0	0.00934	0	0.01008	0.00992	0	0.00860
864	1:1	4	864	asOF	0	0.02469	0	0.02528	0.02503	0	0.02421
864	2:1	2	432	asP	0	0.01547	0	0.01620	0.01580	0	0.01511
864	2:1	2	864	asP	0	0.02535	0	0.02617	0.02571	0	0.02500
864	2:1	3	288	asP	0	0.01149	0	0.01265	0.01212	0	0.01094
864	2:1	3	576	asP	0	0.01892	0	0.02030	0.01970	0	0.01822
864	2:1	3	864	asP	0	0.02504	0	0.02641	0.02575	0	0.02439
864	2:1	4	216	asP	0	0.00899	0	0.01042	0.00973	0	0.00837
864	2:1	4	432	asP	0	0.01554	0	0.01752	0.01666	0	0.01477
864	2:1	4	648	asP	0	0.02069	0	0.02266	0.02179	0	0.01990
864	2:1	4	864	asP	0	0.02531	0	0.02743	0.02656	0	0.02470
864	2:1	2	432	asOF	0	0.00143	0	0.00205	0.00193	0	0.00135
864	2:1	2	864	asOF	0	0.02494	0	0.02553	0.02535	0	0.02488
864	2:1	3	288	asOF	0	0.00008	0	0.00030	0.00029	0	0.00005
864	2:1	3	576	asOF	0	0.00578	0	0.00688	0.00671	0	0.00537
864	2:1	3	864	asOF	0	0.02495	0	0.02568	0.02541	0	0.02461
864	2:1	4	216	asOF	0	0.00000	0	0.00011	0.00006	0	0.00000
864	2:1	4	432	asOF	0	0.00143	0	0.00211	0.00195	0	0.00135
864	2:1	4	648	asOF	0	0.00958	0	0.01054	0.01026	0	0.00902
864	2:1	4	864	asOF	0	0.02483	0	0.02585	0.02550	0	0.02464

Table 5: Normal distribution with $\mu_1 = 0, \mu_2 = 0, \sigma_1 = 1, \sigma_2 = 1$

N_K	AR	K	N_k	f	NA	WMW	NA	BM	BM-T	NA	LWO
1008	1:1	2	504	asP	0	0.01552	0	0.01612	0.01584	0	0.01511
1008	1:1	2	1008	asP	0	0.02491	0	0.02554	0.02521	0	0.02450
1008	1:1	3	336	asP	0	0.01067	0	0.01159	0.01127	0	0.01006
1008	1:1	3	672	asP	0	0.01818	0	0.01923	0.01880	0	0.01747
1008	1:1	3	1008	asP	0	0.02395	0	0.02504	0.02460	0	0.02327
1008	1:1	4	252	asP	0	0.00876	0	0.00961	0.00924	0	0.00803
1008	1:1	4	504	asP	0	0.01517	0	0.01618	0.01572	0	0.01429
1008	1:1	4	756	asP	0	0.02028	0	0.02141	0.02093	0	0.01933
1008	1:1	4	1008	asP	0	0.02442	0	0.02559	0.02511	0	0.02354
1008	1:1	2	504	asOF	0	0.00159	0	0.00189	0.00181	0	0.00127
1008	1:1	2	1008	asOF	0	0.02479	0	0.02509	0.02494	0	0.02455
1008	1:1	3	336	asOF	0	0.00004	0	0.00013	0.00011	0	0.00001
1008	1:1	3	672	asOF	0	0.00573	0	0.00645	0.00626	0	0.00529
1008	1:1	3	1008	asOF	0	0.02463	0	0.02517	0.02497	0	0.02437
1008	1:1	4	252	asOF	0	0.00000	0	0.00002	0.00001	0	0.00000
1008	1:1	4	504	asOF	0	0.00159	0	0.00190	0.00180	0	0.00127
1008	1:1	4	756	asOF	0	0.00959	0	0.01014	0.00999	0	0.00884
1008	1:1	4	1008	asOF	0	0.02460	0	0.02506	0.02483	0	0.02418
1008	2:1	2	504	asP	0	0.01527	0	0.01611	0.01573	0	0.01496
1008	2:1	2	1008	asP	0	0.02470	0	0.02561	0.02517	0	0.02447
1008	2:1	3	336	asP	0	0.01102	0	0.01218	0.01164	0	0.01054
1008	2:1	3	672	asP	0	0.01853	0	0.01989	0.01924	0	0.01799
1008	2:1	3	1008	asP	0	0.02444	0	0.02584	0.02517	0	0.02399
1008	2:1	4	252	asP	0	0.00874	0	0.00975	0.00923	0	0.00801
1008	2:1	4	504	asP	0	0.01533	0	0.01674	0.01600	0	0.01443
1008	2:1	4	756	asP	0	0.02064	0	0.02221	0.02139	0	0.01974
1008	2:1	4	1008	asP	0	0.02487	0	0.02643	0.02560	0	0.02408
1008	2:1	2	504	asOF	0	0.00140	0	0.00198	0.00188	0	0.00124
1008	2:1	2	1008	asOF	0	0.02473	0	0.02521	0.02489	0	0.02447
1008	2:1	3	336	asOF	0	0.00010	0	0.00027	0.00022	0	0.00006
1008	2:1	3	672	asOF	0	0.00570	0	0.00645	0.00622	0	0.00516
1008	2:1	3	1008	asOF	0	0.02444	0	0.02507	0.02476	0	0.02405
1008	2:1	4	252	asOF	0	0.00001	0	0.00009	0.00004	0	0.00000
1008	2:1	4	504	asOF	0	0.00140	0	0.00201	0.00189	0	0.00124
1008	2:1	4	756	asOF	0	0.00968	0	0.01048	0.01026	0	0.00893
1008	2:1	4	1008	asOF	0	0.02454	0	0.02507	0.02483	0	0.02411

Table 6: Normal distribution with $\mu_1 = 0, \mu_2 = 0, \sigma_1 = 1, \sigma_2 = 2$

N_K	AR	K	N_k	f	NA	WMW	NA	BM	BM-T	NA	LWO
144	1:1	2	72	asP	0	0.01919	0	0.020500000	0.017990000	0	0.01241
144	1:1	2	144	asP	0	0.03089	0	0.030630000	0.027740000	0	0.02256
144	1:1	3	48	asP	0	0.01390	4	0.017300692	0.014300572	0	0.00713
144	1:1	3	96	asP	0	0.02351	4	0.026761070	0.023070923	0	0.01448
144	1:1	3	144	asP	0	0.03084	4	0.032951318	0.028901156	0	0.02085
144	1:1	4	36	asP	0	0.01006	36	0.015975751	0.012424473	0	0.00394
144	1:1	4	72	asP	0	0.01836	36	0.024778920	0.020067224	0	0.00965
144	1:1	4	108	asP	0	0.02488	36	0.030691049	0.025739266	0	0.01445
144	1:1	4	144	asP	0	0.03045	36	0.035292705	0.030220880	0	0.01955
144	1:1	2	72	asOF	0	0.00195	0	0.004810000	0.003960000	0	0.00014
144	1:1	2	144	asOF	0	0.02980	0	0.028700000	0.026850000	0	0.02385
144	1:1	3	48	asOF	0	0.00006	4	0.002520101	0.001670067	0	0.00000
144	1:1	3	96	asOF	0	0.00788	4	0.012020481	0.010280411	0	0.00294
144	1:1	3	144	asOF	0	0.03036	4	0.030501220	0.028181127	0	0.02339
144	1:1	4	36	asOF	0	0.00000	33	0.001700561	0.000880290	0	0.00000
144	1:1	4	72	asOF	0	0.00195	33	0.006011984	0.004521492	0	0.00014
144	1:1	4	108	asOF	0	0.01214	33	0.016405414	0.014104655	0	0.00570
144	1:1	4	144	asOF	0	0.03027	33	0.031120270	0.028349355	0	0.02324
144	2:1	2	72	asP	0	0.02908	7	0.021491504	0.017931255	0	0.01148
144	2:1	2	144	asP	0	0.04662	7	0.032432270	0.028051964	0	0.02177
144	2:1	3	48	asP	0	0.02131	122	0.020044454	0.014217345	0	0.00663
144	2:1	3	96	asP	0	0.03616	122	0.030186828	0.022777789	0	0.01390
144	2:1	3	144	asP	0	0.04774	122	0.036544584	0.028725045	0	0.02025
144	2:1	4	36	asP	0	0.01837	519	0.019853037	0.012836622	0	0.00421
144	2:1	4	72	asP	0	0.03062	519	0.029040721	0.020134498	0	0.00965
144	2:1	4	108	asP	0	0.04110	519	0.035524371	0.025612931	0	0.01433
144	2:1	4	144	asP	0	0.04952	519	0.039977483	0.029643852	0	0.01916
144	2:1	2	72	asOF	0	0.00398	7	0.007450522	0.005420379	0	0.00037
144	2:1	2	144	asOF	0	0.04330	7	0.031022172	0.027431920	0	0.02355
144	2:1	3	48	asOF	0	0.00034	117	0.004184896	0.002552987	0	0.00003
144	2:1	3	96	asOF	0	0.01368	117	0.014857383	0.011963998	0	0.00306
144	2:1	3	144	asOF	0	0.04457	117	0.032938538	0.028703583	0	0.02324
144	2:1	4	36	asOF	0	0.00001	505	0.003236344	0.001387004	0	0.00000
144	2:1	4	72	asOF	0	0.00388	505	0.008915021	0.005588221	0	0.00037
144	2:1	4	108	asOF	0	0.02028	505	0.019970853	0.015709332	0	0.00549
144	2:1	4	144	asOF	0	0.04520	505	0.033971556	0.028855721	0	0.02307

Table 7: Normal distribution with $\mu_1 = 0, \mu_2 = 0, \sigma_1 = 1, \sigma_2 = 2$

N_K	AR	K	N_k	f	NA	WMW	NA	BM	BM-T	NA	LWO
288	1:1	2	144	asP	0	0.01898	0	0.017830000	0.016580000	0	0.01420
288	1:1	2	288	asP	0	0.03025	0	0.027510000	0.025950000	0	0.02368
288	1:1	3	96	asP	0	0.01400	0	0.014420000	0.012790000	0	0.00908
288	1:1	3	192	asP	0	0.02377	0	0.023010000	0.021190000	0	0.01695
288	1:1	3	288	asP	0	0.03059	0	0.028910000	0.026980000	0	0.02296
288	1:1	4	72	asP	0	0.01095	0	0.012650000	0.010620000	0	0.00645
288	1:1	4	144	asP	0	0.01934	0	0.020650000	0.018040000	0	0.01272
288	1:1	4	216	asP	0	0.02598	0	0.026460000	0.023790000	0	0.01807
288	1:1	4	288	asP	0	0.03108	0	0.030660000	0.027900000	0	0.02237
288	1:1	2	144	asOF	0	0.00208	0	0.003110000	0.002640000	0	0.00072
288	1:1	2	288	asOF	0	0.02934	0	0.026400000	0.025460000	0	0.02393
288	1:1	3	96	asOF	0	0.00011	0	0.000700000	0.000490000	0	0.00002
288	1:1	3	192	asOF	0	0.00817	0	0.008760000	0.008040000	0	0.00457
288	1:1	3	288	asOF	0	0.02962	0	0.026830000	0.025850000	0	0.02361
288	1:1	4	72	asOF	0	0.00001	0	0.000410000	0.000250000	0	0.00000
288	1:1	4	144	asOF	0	0.00203	0	0.003390000	0.002840000	0	0.00072
288	1:1	4	216	asOF	0	0.01238	0	0.012950000	0.012070000	0	0.00780
288	1:1	4	288	asOF	0	0.02979	0	0.027590000	0.026420000	0	0.02369
288	2:1	2	144	asP	0	0.02885	0	0.018110000	0.016200000	0	0.01318
288	2:1	2	288	asP	0	0.04638	0	0.027810000	0.025350000	0	0.02259
288	2:1	3	96	asP	0	0.02265	0	0.016040000	0.013290000	0	0.00892
288	2:1	3	192	asP	0	0.03706	0	0.024800000	0.021530000	0	0.01627
288	2:1	3	288	asP	0	0.04822	0	0.030640000	0.026980000	0	0.02212
288	2:1	4	72	asP	0	0.01840	8	0.014921194	0.011510921	0	0.00635
288	2:1	4	144	asP	0	0.03123	8	0.022461797	0.018291463	0	0.01219
288	2:1	4	216	asP	0	0.04130	8	0.028282263	0.023811905	0	0.01714
288	2:1	4	288	asP	0	0.04928	8	0.032442595	0.027772222	0	0.02147
288	2:1	2	144	asOF	0	0.00442	0	0.004250000	0.003360000	0	0.00080
288	2:1	2	288	asOF	0	0.04348	0	0.027450000	0.025770000	0	0.02405
288	2:1	3	96	asOF	0	0.00053	0	0.001560000	0.001110000	0	0.00004
288	2:1	3	192	asOF	0	0.01363	0	0.010000000	0.008680000	0	0.00434
288	2:1	3	288	asOF	0	0.04455	0	0.028080000	0.026320000	0	0.02377
288	2:1	4	72	asOF	0	0.00005	8	0.001070086	0.000640051	0	0.00001
288	2:1	4	144	asOF	0	0.00442	8	0.004900392	0.003750300	0	0.00080
288	2:1	4	216	asOF	0	0.02064	8	0.014611169	0.012981038	0	0.00775
288	2:1	4	288	asOF	0	0.04517	8	0.029222338	0.027082167	0	0.02368

Table 8: Normal distribution with $\mu_1 = 0, \mu_2 = 0, \sigma_1 = 1, \sigma_2 = 2$

N_K	AR	K	N_k	f	NA	WMW	NA	BM	BM-T	NA	LWO
576	1:1	2	288	asP	0	0.01943	0	0.01730	0.01674	0	0.01552
576	1:1	2	576	asP	0	0.03093	0	0.02688	0.02614	0	0.02502
576	1:1	3	192	asP	0	0.01433	0	0.01294	0.01210	0	0.01044
576	1:1	3	384	asP	0	0.02411	0	0.02140	0.02030	0	0.01821
576	1:1	3	576	asP	0	0.03116	0	0.02739	0.02623	0	0.02428
576	1:1	4	144	asP	0	0.01064	0	0.01041	0.00951	0	0.00737
576	1:1	4	288	asP	0	0.01942	0	0.01805	0.01688	0	0.01427
576	1:1	4	432	asP	0	0.02573	0	0.02331	0.02203	0	0.01932
576	1:1	4	576	asP	0	0.03089	0	0.02767	0.02634	0	0.02384
576	1:1	2	288	asOF	0	0.00215	0	0.00222	0.00207	0	0.00122
576	1:1	2	576	asOF	0	0.03002	0	0.02587	0.02537	0	0.02471
576	1:1	3	192	asOF	0	0.00008	0	0.00033	0.00025	0	0.00001
576	1:1	3	384	asOF	0	0.00793	0	0.00729	0.00688	0	0.00513
576	1:1	3	576	asOF	0	0.03022	0	0.02636	0.02588	0	0.02475
576	1:1	4	144	asOF	0	0.00000	0	0.00006	0.00004	0	0.00000
576	1:1	4	288	asOF	0	0.00215	0	0.00225	0.00209	0	0.00122
576	1:1	4	432	asOF	0	0.01227	0	0.01095	0.01055	0	0.00859
576	1:1	4	576	asOF	0	0.03000	0	0.02620	0.02574	0	0.02461
576	2:1	2	288	asP	0	0.02999	0	0.01800	0.01686	0	0.01540
576	2:1	2	576	asP	0	0.04730	0	0.02782	0.02647	0	0.02502
576	2:1	3	192	asP	0	0.02337	0	0.01412	0.01268	0	0.01058
576	2:1	3	384	asP	0	0.03826	0	0.02255	0.02080	0	0.01844
576	2:1	3	576	asP	0	0.04925	0	0.02831	0.02635	0	0.02418
576	2:1	4	144	asP	0	0.01823	0	0.01182	0.01017	0	0.00782
576	2:1	4	288	asP	0	0.03225	0	0.01963	0.01748	0	0.01444
576	2:1	4	432	asP	0	0.04231	0	0.02526	0.02289	0	0.01964
576	2:1	4	576	asP	0	0.05041	0	0.02941	0.02707	0	0.02411
576	2:1	2	288	asOF	0	0.00513	0	0.00322	0.00277	0	0.00134
576	2:1	2	576	asOF	0	0.04474	0	0.02626	0.02555	0	0.02463
576	2:1	3	192	asOF	0	0.00056	0	0.00073	0.00052	0	0.00000
576	2:1	3	384	asOF	0	0.01449	0	0.00833	0.00760	0	0.00550
576	2:1	3	576	asOF	0	0.04567	0	0.02683	0.02610	0	0.02485
576	2:1	4	144	asOF	0	0.00005	0	0.00023	0.00012	0	0.00000
576	2:1	4	288	asOF	0	0.00514	0	0.00339	0.00285	0	0.00134
576	2:1	4	432	asOF	0	0.02182	0	0.01266	0.01171	0	0.00859
576	2:1	4	576	asOF	0	0.04685	0	0.02759	0.02653	0	0.02472

Table 9: Normal distribution with $\mu_1 = 0, \mu_2 = 0, \sigma_1 = 1, \sigma_2 = 2$

N_K	AR	K	N_k	f	NA	WMW	NA	BM	BM-T	NA	LWO
864	1:1	2	432	asP	0	0.01974	0	0.01672	0.01635	0	0.01540
864	1:1	2	864	asP	0	0.03087	0	0.02606	0.02564	0	0.02478
864	1:1	3	288	asP	0	0.01443	0	0.01235	0.01200	0	0.01069
864	1:1	3	576	asP	0	0.02440	0	0.02100	0.02048	0	0.01890
864	1:1	3	864	asP	0	0.03143	0	0.02674	0.02609	0	0.02460
864	1:1	4	216	asP	0	0.01118	0	0.01022	0.00962	0	0.00824
864	1:1	4	432	asP	0	0.01979	0	0.01743	0.01665	0	0.01483
864	1:1	4	648	asP	0	0.02629	0	0.02286	0.02204	0	0.02018
864	1:1	4	864	asP	0	0.03144	0	0.02698	0.02615	0	0.02436
864	1:1	2	432	asOF	0	0.00189	0	0.00175	0.00165	0	0.00098
864	1:1	2	864	asOF	0	0.02920	0	0.02531	0.02492	0	0.02440
864	1:1	3	288	asOF	0	0.00010	0	0.00018	0.00014	0	0.00005
864	1:1	3	576	asOF	0	0.00813	0	0.00694	0.00675	0	0.00538
864	1:1	3	864	asOF	0	0.02969	0	0.02550	0.02521	0	0.02448
864	1:1	4	216	asOF	0	0.00000	0	0.00007	0.00006	0	0.00000
864	1:1	4	432	asOF	0	0.00189	0	0.00181	0.00170	0	0.00098
864	1:1	4	648	asOF	0	0.01216	0	0.01050	0.01023	0	0.00875
864	1:1	4	864	asOF	0	0.02978	0	0.02562	0.02525	0	0.02430
864	2:1	2	432	asP	0	0.02895	0	0.01649	0.01574	0	0.01491
864	2:1	2	864	asP	0	0.04603	0	0.02628	0.02544	0	0.02461
864	2:1	3	288	asP	0	0.02312	0	0.01268	0.01165	0	0.01044
864	2:1	3	576	asP	0	0.03783	0	0.02073	0.01960	0	0.01822
864	2:1	3	864	asP	0	0.04853	0	0.02682	0.02566	0	0.02433
864	2:1	4	216	asP	0	0.01875	0	0.01066	0.00961	0	0.00796
864	2:1	4	432	asP	0	0.03204	0	0.01743	0.01617	0	0.01425
864	2:1	4	648	asP	0	0.04136	0	0.02298	0.02162	0	0.01947
864	2:1	4	864	asP	0	0.04950	0	0.02754	0.02608	0	0.02412
864	2:1	2	432	asOF	0	0.00470	0	0.00223	0.00199	0	0.00103
864	2:1	2	864	asOF	0	0.04312	0	0.02558	0.02513	0	0.02459
864	2:1	3	288	asOF	0	0.00050	0	0.00037	0.00028	0	0.00002
864	2:1	3	576	asOF	0	0.01407	0	0.00719	0.00687	0	0.00525
864	2:1	3	864	asOF	0	0.04403	0	0.02588	0.02539	0	0.02452
864	2:1	4	216	asOF	0	0.00008	0	0.00009	0.00007	0	0.00000
864	2:1	4	432	asOF	0	0.00471	0	0.00229	0.00205	0	0.00103
864	2:1	4	648	asOF	0	0.02043	0	0.01105	0.01060	0	0.00874
864	2:1	4	864	asOF	0	0.04438	0	0.02598	0.02540	0	0.02445

Table 10: Normal distribution with $\mu_1 = 0, \mu_2 = 0, \sigma_1 = 1, \sigma_2 = 2$

N_K	AR	K	N_k	f	NA	WMW	NA	BM	BM-T	NA	LWO
1008	1:1	2	504	asP	0	0.01887	0	0.01631	0.01594	0	0.01517
1008	1:1	2	1008	asP	0	0.03028	0	0.02569	0.02524	0	0.02451
1008	1:1	3	336	asP	0	0.01381	0	0.01179	0.01124	0	0.01031
1008	1:1	3	672	asP	0	0.02278	0	0.01934	0.01879	0	0.01773
1008	1:1	3	1008	asP	0	0.02991	0	0.02538	0.02474	0	0.02376
1008	1:1	4	252	asP	0	0.01113	0	0.00980	0.00932	0	0.00814
1008	1:1	4	504	asP	0	0.01910	0	0.01672	0.01607	0	0.01464
1008	1:1	4	756	asP	0	0.02523	0	0.02187	0.02120	0	0.01967
1008	1:1	4	1008	asP	0	0.03040	0	0.02626	0.02550	0	0.02404
1008	1:1	2	504	asOF	0	0.00218	0	0.00200	0.00188	0	0.00129
1008	1:1	2	1008	asOF	0	0.02969	0	0.02555	0.02528	0	0.02484
1008	1:1	3	336	asOF	0	0.00008	0	0.00016	0.00013	0	0.00002
1008	1:1	3	672	asOF	0	0.00765	0	0.00665	0.00642	0	0.00551
1008	1:1	3	1008	asOF	0	0.02961	0	0.02544	0.02519	0	0.02470
1008	1:1	4	252	asOF	0	0.00000	0	0.00001	0.00000	0	0.00000
1008	1:1	4	504	asOF	0	0.00218	0	0.00198	0.00188	0	0.00129
1008	1:1	4	756	asOF	0	0.01234	0	0.01040	0.01013	0	0.00893
1008	1:1	4	1008	asOF	0	0.03014	0	0.02581	0.02542	0	0.02466
1008	2:1	2	504	asP	0	0.02922	0	0.01632	0.01590	0	0.01508
1008	2:1	2	1008	asP	0	0.04628	0	0.02576	0.02520	0	0.02439
1008	2:1	3	336	asP	0	0.02260	0	0.01260	0.01195	0	0.01062
1008	2:1	3	672	asP	0	0.03709	0	0.02013	0.01932	0	0.01783
1008	2:1	3	1008	asP	0	0.04835	0	0.02629	0.02544	0	0.02404
1008	2:1	4	252	asP	0	0.01882	0	0.01030	0.00926	0	0.00794
1008	2:1	4	504	asP	0	0.03210	0	0.01724	0.01597	0	0.01451
1008	2:1	4	756	asP	0	0.04202	0	0.02257	0.02127	0	0.01966
1008	2:1	4	1008	asP	0	0.04950	0	0.02703	0.02571	0	0.02421
1008	2:1	2	504	asOF	0	0.00480	0	0.00218	0.00202	0	0.00118
1008	2:1	2	1008	asOF	0	0.04331	0	0.02528	0.02493	0	0.02431
1008	2:1	3	336	asOF	0	0.00064	0	0.00032	0.00025	0	0.00006
1008	2:1	3	672	asOF	0	0.01388	0	0.00700	0.00670	0	0.00545
1008	2:1	3	1008	asOF	0	0.04405	0	0.02541	0.02500	0	0.02431
1008	2:1	4	252	asOF	0	0.00012	0	0.00012	0.00008	0	0.00000
1008	2:1	4	504	asOF	0	0.00482	0	0.00225	0.00206	0	0.00117
1008	2:1	4	756	asOF	0	0.02116	0	0.01101	0.01054	0	0.00899
1008	2:1	4	1008	asOF	0	0.04468	0	0.02582	0.02525	0	0.02434

Table 11: Normal distribution with $\mu_1 = 0, \mu_2 = 0, \sigma_1 = 2, \sigma_2 = 1$

N_K	AR	K	N_k	f	NA	WMW	NA	BM	BM-T	NA	LWO
144	1:1	2	72	asP	0	0.01908	0	0.019970000	0.017520000	0	0.01241
144	1:1	2	144	asP	0	0.03104	0	0.030470000	0.027630000	0	0.02248
144	1:1	3	48	asP	0	0.01385	3	0.017470524	0.013900417	0	0.00705
144	1:1	3	96	asP	0	0.02322	3	0.026580797	0.022290669	0	0.01392
144	1:1	3	144	asP	0	0.03041	3	0.032870986	0.028270848	0	0.02029
144	1:1	4	36	asP	0	0.01001	33	0.016195344	0.012344074	0	0.00372
144	1:1	4	72	asP	0	0.01858	33	0.025038263	0.020056619	0	0.00982
144	1:1	4	108	asP	0	0.02525	33	0.031200296	0.025838527	0	0.01464
144	1:1	4	144	asP	0	0.03063	33	0.035581742	0.030159953	0	0.01968
144	1:1	2	72	asOF	0	0.00193	0	0.005450000	0.004120000	0	0.00014
144	1:1	2	144	asOF	0	0.03007	0	0.029420000	0.027280000	0	0.02396
144	1:1	3	48	asOF	0	0.00006	3	0.002530076	0.001630049	0	0.00000
144	1:1	3	96	asOF	0	0.00761	3	0.011550347	0.009680290	0	0.00272
144	1:1	3	144	asOF	0	0.03047	3	0.030360911	0.027900837	0	0.02361
144	1:1	4	36	asOF	0	0.00000	33	0.001680555	0.000880290	0	0.00000
144	1:1	4	72	asOF	0	0.00193	33	0.006522152	0.004641532	0	0.00014
144	1:1	4	108	asOF	0	0.01209	33	0.016695510	0.014284714	0	0.00576
144	1:1	4	144	asOF	0	0.03077	33	0.031540408	0.028519411	0	0.02354
144	2:1	2	72	asP	0	0.00935	0	0.018820000	0.017020000	0	0.01224
144	2:1	2	144	asP	0	0.01568	0	0.029000000	0.026800000	0	0.02200
144	2:1	3	48	asP	0	0.00614	0	0.015940000	0.013630000	0	0.00693
144	2:1	3	96	asP	0	0.01075	0	0.025220000	0.022180000	0	0.01403
144	2:1	3	144	asP	0	0.01468	0	0.031320000	0.028150000	0	0.02045
144	2:1	4	36	asP	0	0.00442	8	0.015261221	0.011840947	0	0.00369
144	2:1	4	72	asP	0	0.00804	8	0.023481879	0.019331547	0	0.00913
144	2:1	4	108	asP	0	0.01139	8	0.029682375	0.025182015	0	0.01401
144	2:1	4	144	asP	0	0.01405	8	0.034022722	0.029522362	0	0.01892
144	2:1	2	72	asOF	0	0.00058	0	0.004100000	0.003260000	0	0.00016
144	2:1	2	144	asOF	0	0.01643	0	0.028200000	0.026780000	0	0.02385
144	2:1	3	48	asOF	0	0.00002	0	0.001590000	0.001170000	0	0.00000
144	2:1	3	96	asOF	0	0.00304	0	0.010100000	0.008890000	0	0.00281
144	2:1	3	144	asOF	0	0.01621	0	0.029040000	0.027400000	0	0.02342
144	2:1	4	36	asOF	0	0.00000	8	0.001240099	0.000730058	0	0.00000
144	2:1	4	72	asOF	0	0.00055	8	0.004880390	0.003690295	0	0.00016
144	2:1	4	108	asOF	0	0.00524	8	0.014951196	0.013101048	0	0.00553
144	2:1	4	144	asOF	0	0.01592	8	0.029752380	0.027802224	0	0.02296

Table 12: Normal distribution with $\mu_1 = 0, \mu_2 = 0, \sigma_1 = 2, \sigma_2 = 1$

N_K	AR	K	N_k	f	NA	WMW	NA	BM	BM-T	NA	LWO
288	1:1	2	144	asP	0	0.01879	0	0.01784	0.01661	0	0.01405
288	1:1	2	288	asP	0	0.02961	0	0.02692	0.02546	0	0.02294
288	1:1	3	96	asP	0	0.01351	0	0.01394	0.01224	0	0.00880
288	1:1	3	192	asP	0	0.02278	0	0.02241	0.02030	0	0.01604
288	1:1	3	288	asP	0	0.02940	0	0.02801	0.02571	0	0.02159
288	1:1	4	72	asP	0	0.01113	0	0.01267	0.01107	0	0.00645
288	1:1	4	144	asP	0	0.01939	0	0.02030	0.01810	0	0.01256
288	1:1	4	216	asP	0	0.02586	0	0.02600	0.02359	0	0.01751
288	1:1	4	288	asP	0	0.03056	0	0.02988	0.02739	0	0.02171
288	1:1	2	144	asOF	0	0.00221	0	0.00328	0.00277	0	0.00067
288	1:1	2	288	asOF	0	0.02872	0	0.02601	0.02502	0	0.02321
288	1:1	3	96	asOF	0	0.00016	0	0.00078	0.00058	0	0.00000
288	1:1	3	192	asOF	0	0.00732	0	0.00801	0.00736	0	0.00411
288	1:1	3	288	asOF	0	0.02877	0	0.02612	0.02508	0	0.02286
288	1:1	4	72	asOF	0	0.00001	0	0.00040	0.00023	0	0.00000
288	1:1	4	144	asOF	0	0.00220	0	0.00354	0.00293	0	0.00067
288	1:1	4	216	asOF	0	0.01194	0	0.01249	0.01160	0	0.00725
288	1:1	4	288	asOF	0	0.02922	0	0.02674	0.02559	0	0.02298
288	2:1	2	144	asP	0	0.00898	0	0.01673	0.01589	0	0.01337
288	2:1	2	288	asP	0	0.01469	0	0.02637	0.02529	0	0.02287
288	2:1	3	96	asP	0	0.00619	0	0.01382	0.01247	0	0.00916
288	2:1	3	192	asP	0	0.01074	0	0.02192	0.02032	0	0.01625
288	2:1	3	288	asP	0	0.01437	0	0.02810	0.02633	0	0.02243
288	2:1	4	72	asP	0	0.00476	0	0.01176	0.01043	0	0.00637
288	2:1	4	144	asP	0	0.00833	0	0.01905	0.01711	0	0.01224
288	2:1	4	216	asP	0	0.01141	0	0.02452	0.02248	0	0.01720
288	2:1	4	288	asP	0	0.01402	0	0.02889	0.02680	0	0.02168
288	2:1	2	144	asOF	0	0.00049	0	0.00258	0.00222	0	0.00058
288	2:1	2	288	asOF	0	0.01596	0	0.02610	0.02546	0	0.02408
288	2:1	3	96	asOF	0	0.00003	0	0.00065	0.00055	0	0.00000
288	2:1	3	192	asOF	0	0.00287	0	0.00741	0.00698	0	0.00402
288	2:1	3	288	asOF	0	0.01565	0	0.02643	0.02591	0	0.02373
288	2:1	4	72	asOF	0	0.00000	0	0.00020	0.00013	0	0.00000
288	2:1	4	144	asOF	0	0.00049	0	0.00273	0.00231	0	0.00058
288	2:1	4	216	asOF	0	0.00501	0	0.01098	0.01046	0	0.00693
288	2:1	4	288	asOF	0	0.01543	0	0.02663	0.02577	0	0.02347

Table 13: Normal distribution with $\mu_1 = 0, \mu_2 = 0, \sigma_1 = 2, \sigma_2 = 1$

N_K	AR	K	N_k	f	NA	WMW	NA	BM	BM-T	NA	LWO
576	1:1	2	288	asP	0	0.01880	0	0.01634	0.01577	0	0.01442
576	1:1	2	576	asP	0	0.03053	0	0.02640	0.02561	0	0.02428
576	1:1	3	192	asP	0	0.01414	0	0.01283	0.01212	0	0.01036
576	1:1	3	384	asP	0	0.02395	0	0.02110	0.02020	0	0.01808
576	1:1	3	576	asP	0	0.03124	0	0.02728	0.02625	0	0.02429
576	1:1	4	144	asP	0	0.01115	0	0.01065	0.00984	0	0.00762
576	1:1	4	288	asP	0	0.01938	0	0.01772	0.01666	0	0.01391
576	1:1	4	432	asP	0	0.02595	0	0.02323	0.02210	0	0.01916
576	1:1	4	576	asP	0	0.03119	0	0.02756	0.02638	0	0.02357
576	1:1	2	288	asOF	0	0.00212	0	0.00223	0.00206	0	0.00116
576	1:1	2	576	asOF	0	0.03042	0	0.02617	0.02572	0	0.02503
576	1:1	3	192	asOF	0	0.00007	0	0.00020	0.00017	0	0.00000
576	1:1	3	384	asOF	0	0.00780	0	0.00716	0.00681	0	0.00504
576	1:1	3	576	asOF	0	0.03047	0	0.02629	0.02578	0	0.02482
576	1:1	4	144	asOF	0	0.00002	0	0.00013	0.00006	0	0.00000
576	1:1	4	288	asOF	0	0.00212	0	0.00233	0.00211	0	0.00116
576	1:1	4	432	asOF	0	0.01260	0	0.01120	0.01087	0	0.00879
576	1:1	4	576	asOF	0	0.03050	0	0.02654	0.02617	0	0.02481
576	2:1	2	288	asP	0	0.00963	0	0.01649	0.01617	0	0.01490
576	2:1	2	576	asP	0	0.01574	0	0.02635	0.02592	0	0.02453
576	2:1	3	192	asP	0	0.00660	0	0.01238	0.01186	0	0.01033
576	2:1	3	384	asP	0	0.01125	0	0.02041	0.01984	0	0.01795
576	2:1	3	576	asP	0	0.01517	0	0.02655	0.02589	0	0.02408
576	2:1	4	144	asP	0	0.00491	0	0.01036	0.00967	0	0.00779
576	2:1	4	288	asP	0	0.00905	0	0.01744	0.01652	0	0.01438
576	2:1	4	432	asP	0	0.01244	0	0.02303	0.02209	0	0.01968
576	2:1	4	576	asP	0	0.01508	0	0.02738	0.02640	0	0.02415
576	2:1	2	288	asOF	0	0.00057	0	0.00201	0.00183	0	0.00103
576	2:1	2	576	asOF	0	0.01642	0	0.02594	0.02559	0	0.02485
576	2:1	3	192	asOF	0	0.00002	0	0.00028	0.00024	0	0.00003
576	2:1	3	384	asOF	0	0.00318	0	0.00687	0.00665	0	0.00511
576	2:1	3	576	asOF	0	0.01621	0	0.02591	0.02561	0	0.02462
576	2:1	4	144	asOF	0	0.00000	0	0.00009	0.00005	0	0.00000
576	2:1	4	288	asOF	0	0.00057	0	0.00207	0.00187	0	0.00103
576	2:1	4	432	asOF	0	0.00549	0	0.01098	0.01063	0	0.00886
576	2:1	4	576	asOF	0	0.01633	0	0.02618	0.02570	0	0.02455

Table 14: Normal distribution with $\mu_1 = 0, \mu_2 = 0, \sigma_1 = 2, \sigma_2 = 1$

N_K	AR	K	N_k	f	NA	WMW	NA	BM	BM-T	NA	LWO
864	1:1	2	432	asP	0	0.01892	0	0.01665	0.01616	0	0.01522
864	1:1	2	864	asP	0	0.03032	0	0.02605	0.02553	0	0.02465
864	1:1	3	288	asP	0	0.01376	0	0.01228	0.01169	0	0.01056
864	1:1	3	576	asP	0	0.02370	0	0.02068	0.02000	0	0.01865
864	1:1	3	864	asP	0	0.03080	0	0.02640	0.02568	0	0.02442
864	1:1	4	216	asP	0	0.01055	0	0.00956	0.00890	0	0.00780
864	1:1	4	432	asP	0	0.01885	0	0.01646	0.01560	0	0.01413
864	1:1	4	648	asP	0	0.02509	0	0.02194	0.02098	0	0.01943
864	1:1	4	864	asP	0	0.03009	0	0.02594	0.02498	0	0.02351
864	1:1	2	432	asOF	0	0.00196	0	0.00193	0.00178	0	0.00116
864	1:1	2	864	asOF	0	0.02975	0	0.02564	0.02531	0	0.02476
864	1:1	3	288	asOF	0	0.00019	0	0.00024	0.00023	0	0.00009
864	1:1	3	576	asOF	0	0.00774	0	0.00674	0.00649	0	0.00533
864	1:1	3	864	asOF	0	0.02977	0	0.02563	0.02535	0	0.02479
864	1:1	4	216	asOF	0	0.00002	0	0.00007	0.00006	0	0.00001
864	1:1	4	432	asOF	0	0.00197	0	0.00197	0.00181	0	0.00116
864	1:1	4	648	asOF	0	0.01198	0	0.01070	0.01039	0	0.00888
864	1:1	4	864	asOF	0	0.02991	0	0.02592	0.02542	0	0.02450
864	2:1	2	432	asP	0	0.00983	0	0.01625	0.01604	0	0.01510
864	2:1	2	864	asP	0	0.01547	0	0.02613	0.02586	0	0.02501
864	2:1	3	288	asP	0	0.00666	0	0.01220	0.01180	0	0.01087
864	2:1	3	576	asP	0	0.01101	0	0.01988	0.01943	0	0.01824
864	2:1	3	864	asP	0	0.01442	0	0.02572	0.02521	0	0.02403
864	2:1	4	216	asP	0	0.00539	0	0.00971	0.00944	0	0.00824
864	2:1	4	432	asP	0	0.00909	0	0.01680	0.01644	0	0.01488
864	2:1	4	648	asP	0	0.01218	0	0.02185	0.02146	0	0.01972
864	2:1	4	864	asP	0	0.01479	0	0.02600	0.02556	0	0.02381
864	2:1	2	432	asOF	0	0.00052	0	0.00183	0.00174	0	0.00109
864	2:1	2	864	asOF	0	0.01655	0	0.02581	0.02556	0	0.02512
864	2:1	3	288	asOF	0	0.00002	0	0.00023	0.00021	0	0.00005
864	2:1	3	576	asOF	0	0.00316	0	0.00646	0.00626	0	0.00542
864	2:1	3	864	asOF	0	0.01627	0	0.02576	0.02559	0	0.02491
864	2:1	4	216	asOF	0	0.00000	0	0.00003	0.00003	0	0.00000
864	2:1	4	432	asOF	0	0.00052	0	0.00185	0.00175	0	0.00109
864	2:1	4	648	asOF	0	0.00537	0	0.00990	0.00974	0	0.00860
864	2:1	4	864	asOF	0	0.01608	0	0.02559	0.02543	0	0.02470

Table 15: Normal distribution with $\mu_1 = 0, \mu_2 = 0, \sigma_1 = 2, \sigma_2 = 1$

N_K	AR	K	N_k	f	NA	WMW	NA	BM	BM-T	NA	LWO
1008	1:1	2	504	asP	0	0.01938	0	0.01680	0.01644	0	0.01553
1008	1:1	2	1008	asP	0	0.03074	0	0.02615	0.02576	0	0.02486
1008	1:1	3	336	asP	0	0.01416	0	0.01204	0.01152	0	0.01043
1008	1:1	3	672	asP	0	0.02325	0	0.01958	0.01899	0	0.01778
1008	1:1	3	1008	asP	0	0.03029	0	0.02534	0.02481	0	0.02359
1008	1:1	4	252	asP	0	0.01126	0	0.01018	0.00949	0	0.00804
1008	1:1	4	504	asP	0	0.01949	0	0.01699	0.01612	0	0.01429
1008	1:1	4	756	asP	0	0.02561	0	0.02220	0.02117	0	0.01932
1008	1:1	4	1008	asP	0	0.03097	0	0.02647	0.02545	0	0.02365
1008	1:1	2	504	asOF	0	0.00199	0	0.00179	0.00169	0	0.00119
1008	1:1	2	1008	asOF	0	0.02992	0	0.02549	0.02525	0	0.02485
1008	1:1	3	336	asOF	0	0.00012	0	0.00022	0.00015	0	0.00006
1008	1:1	3	672	asOF	0	0.00772	0	0.00673	0.00646	0	0.00545
1008	1:1	3	1008	asOF	0	0.03009	0	0.02585	0.02552	0	0.02485
1008	1:1	4	252	asOF	0	0.00001	0	0.00002	0.00002	0	0.00000
1008	1:1	4	504	asOF	0	0.00197	0	0.00178	0.00169	0	0.00119
1008	1:1	4	756	asOF	0	0.01200	0	0.01021	0.01001	0	0.00882
1008	1:1	4	1008	asOF	0	0.03033	0	0.02580	0.02557	0	0.02478
1008	2:1	2	504	asP	0	0.00931	0	0.01578	0.01548	0	0.01476
1008	2:1	2	1008	asP	0	0.01521	0	0.02533	0.02495	0	0.02427
1008	2:1	3	336	asP	0	0.00671	0	0.01206	0.01161	0	0.01069
1008	2:1	3	672	asP	0	0.01103	0	0.01969	0.01914	0	0.01806
1008	2:1	3	1008	asP	0	0.01472	0	0.02559	0.02501	0	0.02399
1008	2:1	4	252	asP	0	0.00514	0	0.00989	0.00946	0	0.00824
1008	2:1	4	504	asP	0	0.00877	0	0.01675	0.01619	0	0.01478
1008	2:1	4	756	asP	0	0.01144	0	0.02159	0.02099	0	0.01939
1008	2:1	4	1008	asP	0	0.01421	0	0.02604	0.02542	0	0.02396
1008	2:1	2	504	asOF	0	0.00055	0	0.00173	0.00165	0	0.00119
1008	2:1	2	1008	asOF	0	0.01606	0	0.02504	0.02489	0	0.02453
1008	2:1	3	336	asOF	0	0.00005	0	0.00020	0.00017	0	0.00007
1008	2:1	3	672	asOF	0	0.00288	0	0.00612	0.00598	0	0.00508
1008	2:1	3	1008	asOF	0	0.01580	0	0.02511	0.02496	0	0.02435
1008	2:1	4	252	asOF	0	0.00000	0	0.00002	0.00001	0	0.00000
1008	2:1	4	504	asOF	0	0.00054	0	0.00172	0.00165	0	0.00119
1008	2:1	4	756	asOF	0	0.00469	0	0.00938	0.00924	0	0.00815
1008	2:1	4	1008	asOF	0	0.01554	0	0.02511	0.02487	0	0.02421

Table 16: Ordinal distribution with $\alpha_1 = 5, \beta_1 = 4, \alpha_2 = 5, \beta_2 = 4$

N_K	AR	K	N_k	f	NA	WMW	NA	BM	BM-T	NA	LWO
144	1:1	2	72	asP	0	0.01501	0	0.018970000	0.017170000	0	0.01300
144	1:1	2	144	asP	0	0.02506	0	0.029240000	0.027100000	0	0.02310
144	1:1	3	48	asP	0	0.01073	1	0.016300163	0.013840138	0	0.00823
144	1:1	3	96	asP	0	0.01875	1	0.025400254	0.022440224	0	0.01571
144	1:1	3	144	asP	0	0.02486	1	0.031750318	0.028460285	0	0.02218
144	1:1	4	36	asP	0	0.00808	12	0.015541865	0.012461495	0	0.00538
144	1:1	4	72	asP	0	0.01458	12	0.023842861	0.020172421	0	0.01112
144	1:1	4	108	asP	0	0.02018	12	0.030303636	0.026263152	0	0.01630
144	1:1	4	144	asP	0	0.02445	12	0.034704164	0.030343641	0	0.02099
144	1:1	2	72	asOF	0	0.00113	0	0.004170000	0.003410000	0	0.00039
144	1:1	2	144	asOF	0	0.02497	0	0.027970000	0.026630000	0	0.02403
144	1:1	3	48	asOF	0	0.00005	1	0.001860019	0.001210012	0	0.00000
144	1:1	3	96	asOF	0	0.00586	1	0.010930109	0.009500095	0	0.00375
144	1:1	3	144	asOF	0	0.02485	1	0.029080291	0.027210272	0	0.02370
144	1:1	4	36	asOF	0	0.00001	12	0.001230148	0.000690083	0	0.00000
144	1:1	4	72	asOF	0	0.00114	12	0.005110613	0.003900468	0	0.00039
144	1:1	4	108	asOF	0	0.00977	12	0.015671881	0.014161699	0	0.00711
144	1:1	4	144	asOF	0	0.02490	12	0.029793575	0.027903348	0	0.02359
144	2:1	2	72	asP	0	0.01579	2	0.021450429	0.018840377	0	0.01492
144	2:1	2	144	asP	0	0.02501	2	0.031340627	0.028230565	0	0.02449
144	2:1	3	48	asP	0	0.01078	14	0.018392575	0.015022103	0	0.00955
144	2:1	3	96	asP	0	0.01933	14	0.028503991	0.024203388	0	0.01772
144	2:1	3	144	asP	0	0.02495	14	0.034354810	0.029704159	0	0.02357
144	2:1	4	36	asP	0	0.00804	101	0.018248431	0.013703841	0	0.00683
144	2:1	4	72	asP	0	0.01496	101	0.027818096	0.021992212	0	0.01378
144	2:1	4	108	asP	0	0.02058	101	0.033874213	0.027928207	0	0.01900
144	2:1	4	144	asP	0	0.02440	101	0.037768146	0.031641958	0	0.02325
144	2:1	2	72	asOF	0	0.00118	2	0.006530131	0.005220104	0	0.00072
144	2:1	2	144	asOF	0	0.02505	2	0.029960599	0.027800556	0	0.02440
144	2:1	3	48	asOF	0	0.00002	13	0.003340434	0.002410313	0	0.00000
144	2:1	3	96	asOF	0	0.00624	13	0.014081831	0.011941552	0	0.00487
144	2:1	3	144	asOF	0	0.02521	13	0.031784132	0.029143789	0	0.02462
144	2:1	4	36	asOF	0	0.00000	97	0.002021961	0.001171136	0	0.00000
144	2:1	4	72	asOF	0	0.00118	97	0.007727496	0.005865690	0	0.00072
144	2:1	4	108	asOF	0	0.01008	97	0.018838273	0.016425933	0	0.00804
144	2:1	4	144	asOF	0	0.02535	97	0.032471497	0.029588701	0	0.02420

Table 17: Ordinal distribution with $\alpha_1 = 5, \beta_1 = 4, \alpha_2 = 5, \beta_2 = 4$

N_K	AR	K	N_k	f	NA	WMW	NA	BM	BM-T	NA	LWO
288	1:1	2	144	asP	0	0.01557	0	0.01775	0.01682	0	0.01451
288	1:1	2	288	asP	0	0.02506	0	0.02723	0.02626	0	0.02404
288	1:1	3	96	asP	0	0.01102	0	0.01372	0.01270	0	0.00986
288	1:1	3	192	asP	0	0.01897	0	0.02219	0.02110	0	0.01759
288	1:1	3	288	asP	0	0.02501	0	0.02827	0.02712	0	0.02374
288	1:1	4	72	asP	0	0.00838	0	0.01188	0.01037	0	0.00679
288	1:1	4	144	asP	0	0.01489	0	0.01937	0.01746	0	0.01303
288	1:1	4	216	asP	0	0.01991	0	0.02480	0.02277	0	0.01779
288	1:1	4	288	asP	0	0.02446	0	0.02946	0.02732	0	0.02248
288	1:1	2	144	asOF	0	0.00137	0	0.00277	0.00240	0	0.00092
288	1:1	2	288	asOF	0	0.02480	0	0.02636	0.02564	0	0.02426
288	1:1	3	96	asOF	0	0.00004	0	0.00066	0.00049	0	0.00001
288	1:1	3	192	asOF	0	0.00596	0	0.00832	0.00789	0	0.00501
288	1:1	3	288	asOF	0	0.02510	0	0.02696	0.02639	0	0.02445
288	1:1	4	72	asOF	0	0.00001	0	0.00031	0.00022	0	0.00000
288	1:1	4	144	asOF	0	0.00137	0	0.00293	0.00251	0	0.00092
288	1:1	4	216	asOF	0	0.00903	0	0.01193	0.01118	0	0.00750
288	1:1	4	288	asOF	0	0.02477	0	0.02715	0.02628	0	0.02384
288	2:1	2	144	asP	0	0.01571	0	0.01867	0.01737	0	0.01503
288	2:1	2	288	asP	0	0.02504	0	0.02826	0.02660	0	0.02449
288	2:1	3	96	asP	0	0.01108	0	0.01512	0.01352	0	0.01027
288	2:1	3	192	asP	0	0.01937	0	0.02444	0.02249	0	0.01831
288	2:1	3	288	asP	0	0.02499	0	0.03004	0.02782	0	0.02401
288	2:1	4	72	asP	0	0.00873	0	0.01370	0.01155	0	0.00794
288	2:1	4	144	asP	0	0.01533	0	0.02147	0.01875	0	0.01430
288	2:1	4	216	asP	0	0.02053	0	0.02726	0.02434	0	0.01965
288	2:1	4	288	asP	0	0.02462	0	0.03143	0.02843	0	0.02394
288	2:1	2	144	asOF	0	0.00139	0	0.00358	0.00304	0	0.00119
288	2:1	2	288	asOF	0	0.02517	0	0.02726	0.02617	0	0.02482
288	2:1	3	96	asOF	0	0.00002	0	0.00116	0.00069	0	0.00002
288	2:1	3	192	asOF	0	0.00613	0	0.00955	0.00860	0	0.00531
288	2:1	3	288	asOF	0	0.02508	0	0.02796	0.02682	0	0.02490
288	2:1	4	72	asOF	0	0.00001	0	0.00052	0.00028	0	0.00000
288	2:1	4	144	asOF	0	0.00140	0	0.00393	0.00322	0	0.00119
288	2:1	4	216	asOF	0	0.00958	0	0.01377	0.01259	0	0.00872
288	2:1	4	288	asOF	0	0.02525	0	0.02856	0.02709	0	0.02457

Table 18: Ordinal distribution with $\alpha_1 = 5, \beta_1 = 4, \alpha_2 = 5, \beta_2 = 4$

N_K	AR	K	N_k	f	NA	WMW	NA	BM	BM-T	NA	LWO
576	1:1	2	288	asP	0	0.01597	0	0.01714	0.01663	0	0.01553
576	1:1	2	576	asP	0	0.02477	0	0.02609	0.02544	0	0.02434
576	1:1	3	192	asP	0	0.01113	0	0.01266	0.01197	0	0.01047
576	1:1	3	384	asP	0	0.01931	0	0.02113	0.02042	0	0.01859
576	1:1	3	576	asP	0	0.02490	0	0.02672	0.02595	0	0.02421
576	1:1	4	144	asP	0	0.00867	0	0.01028	0.00964	0	0.00803
576	1:1	4	288	asP	0	0.01543	0	0.01764	0.01681	0	0.01468
576	1:1	4	432	asP	0	0.02061	0	0.02286	0.02195	0	0.01979
576	1:1	4	576	asP	0	0.02468	0	0.02695	0.02599	0	0.02394
576	1:1	2	288	asOF	0	0.00188	0	0.00238	0.00223	0	0.00141
576	1:1	2	576	asOF	0	0.02489	0	0.02555	0.02529	0	0.02458
576	1:1	3	192	asOF	0	0.00009	0	0.00039	0.00034	0	0.00007
576	1:1	3	384	asOF	0	0.00619	0	0.00743	0.00719	0	0.00568
576	1:1	3	576	asOF	0	0.02484	0	0.02593	0.02546	0	0.02448
576	1:1	4	144	asOF	0	0.00000	0	0.00011	0.00007	0	0.00000
576	1:1	4	288	asOF	0	0.00188	0	0.00248	0.00229	0	0.00140
576	1:1	4	432	asOF	0	0.00985	0	0.01088	0.01054	0	0.00897
576	1:1	4	576	asOF	0	0.02474	0	0.02583	0.02532	0	0.02420
576	2:1	2	288	asP	0	0.01587	0	0.01736	0.01671	0	0.01550
576	2:1	2	576	asP	0	0.02508	0	0.02667	0.02586	0	0.02472
576	2:1	3	192	asP	0	0.01124	0	0.01316	0.01228	0	0.01091
576	2:1	3	384	asP	0	0.01887	0	0.02130	0.02013	0	0.01847
576	2:1	3	576	asP	0	0.02453	0	0.02691	0.02576	0	0.02415
576	2:1	4	144	asP	0	0.00880	0	0.01132	0.01039	0	0.00849
576	2:1	4	288	asP	0	0.01571	0	0.01882	0.01768	0	0.01536
576	2:1	4	432	asP	0	0.02082	0	0.02407	0.02280	0	0.02036
576	2:1	4	576	asP	0	0.02502	0	0.02835	0.02702	0	0.02465
576	2:1	2	288	asOF	0	0.00142	0	0.00245	0.00220	0	0.00131
576	2:1	2	576	asOF	0	0.02439	0	0.02559	0.02516	0	0.02457
576	2:1	3	192	asOF	0	0.00009	0	0.00044	0.00035	0	0.00004
576	2:1	3	384	asOF	0	0.00581	0	0.00746	0.00706	0	0.00540
576	2:1	3	576	asOF	0	0.02466	0	0.02611	0.02553	0	0.02452
576	2:1	4	144	asOF	0	0.00001	0	0.00016	0.00011	0	0.00000
576	2:1	4	288	asOF	0	0.00143	0	0.00249	0.00222	0	0.00130
576	2:1	4	432	asOF	0	0.00945	0	0.01105	0.01068	0	0.00889
576	2:1	4	576	asOF	0	0.02485	0	0.02613	0.02568	0	0.02460

Table 19: Ordinal distribution with $\alpha_1 = 5, \beta_1 = 4, \alpha_2 = 5, \beta_2 = 4$

N_K	AR	K	N_k	f	NA	WMW	NA	BM	BM-T	NA	LWO
864	1:1	2	432	asP	0	0.01553	0	0.01615	0.01590	0	0.01524
864	1:1	2	864	asP	0	0.02507	0	0.02580	0.02546	0	0.02481
864	1:1	3	288	asP	0	0.01115	0	0.01218	0.01179	0	0.01076
864	1:1	3	576	asP	0	0.01889	0	0.02017	0.01966	0	0.01846
864	1:1	3	864	asP	0	0.02467	0	0.02600	0.02542	0	0.02431
864	1:1	4	216	asP	0	0.00909	0	0.01013	0.00979	0	0.00860
864	1:1	4	432	asP	0	0.01528	0	0.01675	0.01621	0	0.01473
864	1:1	4	648	asP	0	0.02069	0	0.02240	0.02182	0	0.02012
864	1:1	4	864	asP	0	0.02493	0	0.02659	0.02598	0	0.02440
864	1:1	2	432	asOF	0	0.00146	0	0.00187	0.00179	0	0.00128
864	1:1	2	864	asOF	0	0.02499	0	0.02537	0.02525	0	0.02481
864	1:1	3	288	asOF	0	0.00005	0	0.00020	0.00018	0	0.00003
864	1:1	3	576	asOF	0	0.00604	0	0.00674	0.00651	0	0.00559
864	1:1	3	864	asOF	0	0.02513	0	0.02554	0.02533	0	0.02490
864	1:1	4	216	asOF	0	0.00001	0	0.00003	0.00003	0	0.00000
864	1:1	4	432	asOF	0	0.00147	0	0.00188	0.00180	0	0.00128
864	1:1	4	648	asOF	0	0.00963	0	0.01054	0.01035	0	0.00906
864	1:1	4	864	asOF	0	0.02529	0	0.02594	0.02575	0	0.02497
864	2:1	2	432	asP	0	0.01541	0	0.01630	0.01591	0	0.01534
864	2:1	2	864	asP	0	0.02507	0	0.02605	0.02561	0	0.02508
864	2:1	3	288	asP	0	0.01152	0	0.01282	0.01218	0	0.01134
864	2:1	3	576	asP	0	0.01943	0	0.02101	0.02025	0	0.01923
864	2:1	3	864	asP	0	0.02532	0	0.02698	0.02617	0	0.02522
864	2:1	4	216	asP	0	0.00919	0	0.01065	0.01006	0	0.00892
864	2:1	4	432	asP	0	0.01578	0	0.01778	0.01703	0	0.01563
864	2:1	4	648	asP	0	0.02069	0	0.02290	0.02207	0	0.02053
864	2:1	4	864	asP	0	0.02504	0	0.02720	0.02632	0	0.02486
864	2:1	2	432	asOF	0	0.00147	0	0.00210	0.00198	0	0.00139
864	2:1	2	864	asOF	0	0.02554	0	0.02600	0.02575	0	0.02536
864	2:1	3	288	asOF	0	0.00007	0	0.00032	0.00027	0	0.00006
864	2:1	3	576	asOF	0	0.00626	0	0.00725	0.00705	0	0.00606
864	2:1	3	864	asOF	0	0.02563	0	0.02649	0.02616	0	0.02562
864	2:1	4	216	asOF	0	0.00000	0	0.00007	0.00007	0	0.00000
864	2:1	4	432	asOF	0	0.00146	0	0.00214	0.00202	0	0.00139
864	2:1	4	648	asOF	0	0.00957	0	0.01105	0.01074	0	0.00937
864	2:1	4	864	asOF	0	0.02536	0	0.02652	0.02607	0	0.02532

Table 20: Ordinal distribution with $\alpha_1 = 5, \beta_1 = 4, \alpha_2 = 5, \beta_2 = 4$

N_K	AR	K	N_k	f	NA	WMW	NA	BM	BM-T	NA	LWO
1008	1:1	2	504	asP	0	0.01572	0	0.01634	0.01605	0	0.01544
1008	1:1	2	1008	asP	0	0.02505	0	0.02569	0.02535	0	0.02481
1008	1:1	3	336	asP	0	0.01132	0	0.01209	0.01171	0	0.01099
1008	1:1	3	672	asP	0	0.01889	0	0.01980	0.01938	0	0.01850
1008	1:1	3	1008	asP	0	0.02474	0	0.02569	0.02521	0	0.02435
1008	1:1	4	252	asP	0	0.00914	0	0.00999	0.00963	0	0.00872
1008	1:1	4	504	asP	0	0.01542	0	0.01657	0.01612	0	0.01496
1008	1:1	4	756	asP	0	0.02023	0	0.02149	0.02099	0	0.01970
1008	1:1	4	1008	asP	0	0.02456	0	0.02586	0.02532	0	0.02408
1008	1:1	2	504	asOF	0	0.00168	0	0.00202	0.00197	0	0.00145
1008	1:1	2	1008	asOF	0	0.02488	0	0.02522	0.02507	0	0.02469
1008	1:1	3	336	asOF	0	0.00011	0	0.00024	0.00021	0	0.00007
1008	1:1	3	672	asOF	0	0.00599	0	0.00656	0.00642	0	0.00566
1008	1:1	3	1008	asOF	0	0.02483	0	0.02535	0.02519	0	0.02459
1008	1:1	4	252	asOF	0	0.00000	0	0.00004	0.00001	0	0.00000
1008	1:1	4	504	asOF	0	0.00168	0	0.00203	0.00197	0	0.00144
1008	1:1	4	756	asOF	0	0.00938	0	0.01007	0.00992	0	0.00884
1008	1:1	4	1008	asOF	0	0.02487	0	0.02538	0.02518	0	0.02454
1008	2:1	2	504	asP	0	0.01584	0	0.01643	0.01622	0	0.01565
1008	2:1	2	1008	asP	0	0.02528	0	0.02587	0.02555	0	0.02497
1008	2:1	3	336	asP	0	0.01127	0	0.01247	0.01199	0	0.01115
1008	2:1	3	672	asP	0	0.01896	0	0.02050	0.01989	0	0.01874
1008	2:1	3	1008	asP	0	0.02479	0	0.02645	0.02578	0	0.02462
1008	2:1	4	252	asP	0	0.00856	0	0.00990	0.00924	0	0.00821
1008	2:1	4	504	asP	0	0.01542	0	0.01695	0.01613	0	0.01496
1008	2:1	4	756	asP	0	0.02042	0	0.02212	0.02129	0	0.01993
1008	2:1	4	1008	asP	0	0.02472	0	0.02645	0.02561	0	0.02433
1008	2:1	2	504	asOF	0	0.00160	0	0.00210	0.00199	0	0.00149
1008	2:1	2	1008	asOF	0	0.02499	0	0.02560	0.02534	0	0.02500
1008	2:1	3	336	asOF	0	0.00007	0	0.00026	0.00021	0	0.00009
1008	2:1	3	672	asOF	0	0.00598	0	0.00692	0.00677	0	0.00584
1008	2:1	3	1008	asOF	0	0.02501	0	0.02570	0.02542	0	0.02491
1008	2:1	4	252	asOF	0	0.00000	0	0.00004	0.00004	0	0.00001
1008	2:1	4	504	asOF	0	0.00159	0	0.00210	0.00199	0	0.00149
1008	2:1	4	756	asOF	0	0.00957	0	0.01050	0.01034	0	0.00937
1008	2:1	4	1008	asOF	0	0.02478	0	0.02567	0.02537	0	0.02477

Table 21: Ordinal distribution with $\alpha_1 = 3, \beta_1 = 3, \alpha_2 = 1, \beta_2 = 1$

N_K	AR	K	N_k	f	NA	WMW	NA	BM	BM-T	NA	LWO
144	1:1	2	72	asP	0	0.01501	0	0.018970000	0.017170000	0	0.01300
144	1:1	2	144	asP	0	0.02506	0	0.029240000	0.027100000	0	0.02310
144	1:1	3	48	asP	0	0.01073	1	0.016300163	0.013840138	0	0.00823
144	1:1	3	96	asP	0	0.01875	1	0.025400254	0.022440224	0	0.01571
144	1:1	3	144	asP	0	0.02486	1	0.031750318	0.028460285	0	0.02218
144	1:1	4	36	asP	0	0.00808	12	0.015541865	0.012461495	0	0.00538
144	1:1	4	72	asP	0	0.01458	12	0.023842861	0.020172421	0	0.01112
144	1:1	4	108	asP	0	0.02018	12	0.030303636	0.026263152	0	0.01630
144	1:1	4	144	asP	0	0.02445	12	0.034704164	0.030343641	0	0.02099
144	1:1	2	72	asOF	0	0.00113	0	0.004170000	0.003410000	0	0.00039
144	1:1	2	144	asOF	0	0.02497	0	0.027970000	0.026630000	0	0.02403
144	1:1	3	48	asOF	0	0.00005	1	0.001860019	0.001210012	0	0.00000
144	1:1	3	96	asOF	0	0.00586	1	0.010930109	0.009500095	0	0.00375
144	1:1	3	144	asOF	0	0.02485	1	0.029080291	0.027210272	0	0.02370
144	1:1	4	36	asOF	0	0.00001	12	0.001230148	0.000690083	0	0.00000
144	1:1	4	72	asOF	0	0.00114	12	0.005110613	0.003900468	0	0.00039
144	1:1	4	108	asOF	0	0.00977	12	0.015671881	0.014161699	0	0.00711
144	1:1	4	144	asOF	0	0.02490	12	0.029793575	0.027903348	0	0.02359
144	2:1	2	72	asP	0	0.01579	2	0.021450429	0.018840377	0	0.01492
144	2:1	2	144	asP	0	0.02501	2	0.031340627	0.028230565	0	0.02449
144	2:1	3	48	asP	0	0.01078	14	0.018392575	0.015022103	0	0.00955
144	2:1	3	96	asP	0	0.01933	14	0.028503991	0.024203388	0	0.01772
144	2:1	3	144	asP	0	0.02495	14	0.034354810	0.029704159	0	0.02357
144	2:1	4	36	asP	0	0.00804	101	0.018248431	0.013703841	0	0.00683
144	2:1	4	72	asP	0	0.01496	101	0.027818096	0.021992212	0	0.01378
144	2:1	4	108	asP	0	0.02058	101	0.033874213	0.027928207	0	0.01900
144	2:1	4	144	asP	0	0.02440	101	0.037768146	0.031641958	0	0.02325
144	2:1	2	72	asOF	0	0.00118	2	0.006530131	0.005220104	0	0.00072
144	2:1	2	144	asOF	0	0.02505	2	0.029960599	0.027800556	0	0.02440
144	2:1	3	48	asOF	0	0.00002	13	0.003340434	0.002410313	0	0.00000
144	2:1	3	96	asOF	0	0.00624	13	0.014081831	0.011941552	0	0.00487
144	2:1	3	144	asOF	0	0.02521	13	0.031784132	0.029143789	0	0.02462
144	2:1	4	36	asOF	0	0.00000	97	0.002021961	0.001171136	0	0.00000
144	2:1	4	72	asOF	0	0.00118	97	0.007727496	0.005865690	0	0.00072
144	2:1	4	108	asOF	0	0.01008	97	0.018838273	0.016425933	0	0.00804
144	2:1	4	144	asOF	0	0.02535	97	0.032471497	0.029588701	0	0.02420

Table 22: Ordinal distribution with $\alpha_1 = 3, \beta_1 = 3, \alpha_2 = 1, \beta_2 = 1$

N_K	AR	K	N_k	f	NA	WMW	NA	BM	BM-T	NA	LWO
288	1:1	2	144	asP	0	0.01557	0	0.01775	0.01682	0	0.01451
288	1:1	2	288	asP	0	0.02506	0	0.02723	0.02626	0	0.02404
288	1:1	3	96	asP	0	0.01102	0	0.01372	0.01270	0	0.00986
288	1:1	3	192	asP	0	0.01897	0	0.02219	0.02110	0	0.01759
288	1:1	3	288	asP	0	0.02501	0	0.02827	0.02712	0	0.02374
288	1:1	4	72	asP	0	0.00838	0	0.01188	0.01037	0	0.00679
288	1:1	4	144	asP	0	0.01489	0	0.01937	0.01746	0	0.01303
288	1:1	4	216	asP	0	0.01991	0	0.02480	0.02277	0	0.01779
288	1:1	4	288	asP	0	0.02446	0	0.02946	0.02732	0	0.02248
288	1:1	2	144	asOF	0	0.00137	0	0.00277	0.00240	0	0.00092
288	1:1	2	288	asOF	0	0.02480	0	0.02636	0.02564	0	0.02426
288	1:1	3	96	asOF	0	0.00004	0	0.00066	0.00049	0	0.00001
288	1:1	3	192	asOF	0	0.00596	0	0.00832	0.00789	0	0.00501
288	1:1	3	288	asOF	0	0.02510	0	0.02696	0.02639	0	0.02445
288	1:1	4	72	asOF	0	0.00001	0	0.00031	0.00022	0	0.00000
288	1:1	4	144	asOF	0	0.00137	0	0.00293	0.00251	0	0.00092
288	1:1	4	216	asOF	0	0.00903	0	0.01193	0.01118	0	0.00750
288	1:1	4	288	asOF	0	0.02477	0	0.02715	0.02628	0	0.02384
288	2:1	2	144	asP	0	0.01571	0	0.01867	0.01737	0	0.01503
288	2:1	2	288	asP	0	0.02504	0	0.02826	0.02660	0	0.02449
288	2:1	3	96	asP	0	0.01108	0	0.01512	0.01352	0	0.01027
288	2:1	3	192	asP	0	0.01937	0	0.02444	0.02249	0	0.01831
288	2:1	3	288	asP	0	0.02499	0	0.03004	0.02782	0	0.02401
288	2:1	4	72	asP	0	0.00873	0	0.01370	0.01155	0	0.00794
288	2:1	4	144	asP	0	0.01533	0	0.02147	0.01875	0	0.01430
288	2:1	4	216	asP	0	0.02053	0	0.02726	0.02434	0	0.01965
288	2:1	4	288	asP	0	0.02462	0	0.03143	0.02843	0	0.02394
288	2:1	2	144	asOF	0	0.00139	0	0.00358	0.00304	0	0.00119
288	2:1	2	288	asOF	0	0.02517	0	0.02726	0.02617	0	0.02482
288	2:1	3	96	asOF	0	0.00002	0	0.00116	0.00069	0	0.00002
288	2:1	3	192	asOF	0	0.00613	0	0.00955	0.00860	0	0.00531
288	2:1	3	288	asOF	0	0.02508	0	0.02796	0.02682	0	0.02490
288	2:1	4	72	asOF	0	0.00001	0	0.00052	0.00028	0	0.00000
288	2:1	4	144	asOF	0	0.00140	0	0.00393	0.00322	0	0.00119
288	2:1	4	216	asOF	0	0.00958	0	0.01377	0.01259	0	0.00872
288	2:1	4	288	asOF	0	0.02525	0	0.02856	0.02709	0	0.02457

Table 23: Ordinal distribution with $\alpha_1 = 3, \beta_1 = 3, \alpha_2 = 1, \beta_2 = 1$

N_K	AR	K	N_k	f	NA	WMW	NA	BM	BM-T	NA	LWO
576	1:1	2	288	asP	0	0.01597	0	0.01714	0.01663	0	0.01553
576	1:1	2	576	asP	0	0.02477	0	0.02609	0.02544	0	0.02434
576	1:1	3	192	asP	0	0.01113	0	0.01266	0.01197	0	0.01047
576	1:1	3	384	asP	0	0.01931	0	0.02113	0.02042	0	0.01859
576	1:1	3	576	asP	0	0.02490	0	0.02672	0.02595	0	0.02421
576	1:1	4	144	asP	0	0.00867	0	0.01028	0.00964	0	0.00803
576	1:1	4	288	asP	0	0.01543	0	0.01764	0.01681	0	0.01468
576	1:1	4	432	asP	0	0.02061	0	0.02286	0.02195	0	0.01979
576	1:1	4	576	asP	0	0.02468	0	0.02695	0.02599	0	0.02394
576	1:1	2	288	asOF	0	0.00188	0	0.00238	0.00223	0	0.00141
576	1:1	2	576	asOF	0	0.02489	0	0.02555	0.02529	0	0.02458
576	1:1	3	192	asOF	0	0.00009	0	0.00039	0.00034	0	0.00007
576	1:1	3	384	asOF	0	0.00619	0	0.00743	0.00719	0	0.00568
576	1:1	3	576	asOF	0	0.02484	0	0.02593	0.02546	0	0.02448
576	1:1	4	144	asOF	0	0.00000	0	0.00011	0.00007	0	0.00000
576	1:1	4	288	asOF	0	0.00188	0	0.00248	0.00229	0	0.00140
576	1:1	4	432	asOF	0	0.00985	0	0.01088	0.01054	0	0.00897
576	1:1	4	576	asOF	0	0.02474	0	0.02583	0.02532	0	0.02420
576	2:1	2	288	asP	0	0.01587	0	0.01736	0.01671	0	0.01550
576	2:1	2	576	asP	0	0.02508	0	0.02667	0.02586	0	0.02472
576	2:1	3	192	asP	0	0.01124	0	0.01316	0.01228	0	0.01091
576	2:1	3	384	asP	0	0.01887	0	0.02130	0.02013	0	0.01847
576	2:1	3	576	asP	0	0.02453	0	0.02691	0.02576	0	0.02415
576	2:1	4	144	asP	0	0.00880	0	0.01132	0.01039	0	0.00849
576	2:1	4	288	asP	0	0.01571	0	0.01882	0.01768	0	0.01536
576	2:1	4	432	asP	0	0.02082	0	0.02407	0.02280	0	0.02036
576	2:1	4	576	asP	0	0.02502	0	0.02835	0.02702	0	0.02465
576	2:1	2	288	asOF	0	0.00142	0	0.00245	0.00220	0	0.00131
576	2:1	2	576	asOF	0	0.02439	0	0.02559	0.02516	0	0.02457
576	2:1	3	192	asOF	0	0.00009	0	0.00044	0.00035	0	0.00004
576	2:1	3	384	asOF	0	0.00581	0	0.00746	0.00706	0	0.00540
576	2:1	3	576	asOF	0	0.02466	0	0.02611	0.02553	0	0.02452
576	2:1	4	144	asOF	0	0.00001	0	0.00016	0.00011	0	0.00000
576	2:1	4	288	asOF	0	0.00143	0	0.00249	0.00222	0	0.00130
576	2:1	4	432	asOF	0	0.00945	0	0.01105	0.01068	0	0.00889
576	2:1	4	576	asOF	0	0.02485	0	0.02613	0.02568	0	0.02460

Table 24: Ordinal distribution with $\alpha_1 = 3, \beta_1 = 3, \alpha_2 = 1, \beta_2 = 1$

N_K	AR	K	N_k	f	NA	WMW	NA	BM	BM-T	NA	LWO
864	1:1	2	432	asP	0	0.01553	0	0.01615	0.01590	0	0.01524
864	1:1	2	864	asP	0	0.02507	0	0.02580	0.02546	0	0.02481
864	1:1	3	288	asP	0	0.01115	0	0.01218	0.01179	0	0.01076
864	1:1	3	576	asP	0	0.01889	0	0.02017	0.01966	0	0.01846
864	1:1	3	864	asP	0	0.02467	0	0.02600	0.02542	0	0.02431
864	1:1	4	216	asP	0	0.00909	0	0.01013	0.00979	0	0.00860
864	1:1	4	432	asP	0	0.01528	0	0.01675	0.01621	0	0.01473
864	1:1	4	648	asP	0	0.02069	0	0.02240	0.02182	0	0.02012
864	1:1	4	864	asP	0	0.02493	0	0.02659	0.02598	0	0.02440
864	1:1	2	432	asOF	0	0.00146	0	0.00187	0.00179	0	0.00128
864	1:1	2	864	asOF	0	0.02499	0	0.02537	0.02525	0	0.02481
864	1:1	3	288	asOF	0	0.00005	0	0.00020	0.00018	0	0.00003
864	1:1	3	576	asOF	0	0.00604	0	0.00674	0.00651	0	0.00559
864	1:1	3	864	asOF	0	0.02513	0	0.02554	0.02533	0	0.02490
864	1:1	4	216	asOF	0	0.00001	0	0.00003	0.00003	0	0.00000
864	1:1	4	432	asOF	0	0.00147	0	0.00188	0.00180	0	0.00128
864	1:1	4	648	asOF	0	0.00963	0	0.01054	0.01035	0	0.00906
864	1:1	4	864	asOF	0	0.02529	0	0.02594	0.02575	0	0.02497
864	2:1	2	432	asP	0	0.01541	0	0.01630	0.01591	0	0.01534
864	2:1	2	864	asP	0	0.02507	0	0.02605	0.02561	0	0.02508
864	2:1	3	288	asP	0	0.01152	0	0.01282	0.01218	0	0.01134
864	2:1	3	576	asP	0	0.01943	0	0.02101	0.02025	0	0.01923
864	2:1	3	864	asP	0	0.02532	0	0.02698	0.02617	0	0.02522
864	2:1	4	216	asP	0	0.00919	0	0.01065	0.01006	0	0.00892
864	2:1	4	432	asP	0	0.01578	0	0.01778	0.01703	0	0.01563
864	2:1	4	648	asP	0	0.02069	0	0.02290	0.02207	0	0.02053
864	2:1	4	864	asP	0	0.02504	0	0.02720	0.02632	0	0.02486
864	2:1	2	432	asOF	0	0.00147	0	0.00210	0.00198	0	0.00139
864	2:1	2	864	asOF	0	0.02554	0	0.02600	0.02575	0	0.02536
864	2:1	3	288	asOF	0	0.00007	0	0.00032	0.00027	0	0.00006
864	2:1	3	576	asOF	0	0.00626	0	0.00725	0.00705	0	0.00606
864	2:1	3	864	asOF	0	0.02563	0	0.02649	0.02616	0	0.02562
864	2:1	4	216	asOF	0	0.00000	0	0.00007	0.00007	0	0.00000
864	2:1	4	432	asOF	0	0.00146	0	0.00214	0.00202	0	0.00139
864	2:1	4	648	asOF	0	0.00957	0	0.01105	0.01074	0	0.00937
864	2:1	4	864	asOF	0	0.02536	0	0.02652	0.02607	0	0.02532

Table 25: Ordinal distribution with $\alpha_1 = 3, \beta_1 = 3, \alpha_2 = 1, \beta_2 = 1$

N_K	AR	K	N_k	f	NA	WMW	NA	BM	BM-T	NA	LWO
1008	1:1	2	504	asP	0	0.01572	0	0.01634	0.01605	0	0.01544
1008	1:1	2	1008	asP	0	0.02505	0	0.02569	0.02535	0	0.02481
1008	1:1	3	336	asP	0	0.01132	0	0.01209	0.01171	0	0.01099
1008	1:1	3	672	asP	0	0.01889	0	0.01980	0.01938	0	0.01850
1008	1:1	3	1008	asP	0	0.02474	0	0.02569	0.02521	0	0.02435
1008	1:1	4	252	asP	0	0.00914	0	0.00999	0.00963	0	0.00872
1008	1:1	4	504	asP	0	0.01542	0	0.01657	0.01612	0	0.01496
1008	1:1	4	756	asP	0	0.02023	0	0.02149	0.02099	0	0.01970
1008	1:1	4	1008	asP	0	0.02456	0	0.02586	0.02532	0	0.02408
1008	1:1	2	504	asOF	0	0.00168	0	0.00202	0.00197	0	0.00145
1008	1:1	2	1008	asOF	0	0.02488	0	0.02522	0.02507	0	0.02469
1008	1:1	3	336	asOF	0	0.00011	0	0.00024	0.00021	0	0.00007
1008	1:1	3	672	asOF	0	0.00599	0	0.00656	0.00642	0	0.00566
1008	1:1	3	1008	asOF	0	0.02483	0	0.02535	0.02519	0	0.02459
1008	1:1	4	252	asOF	0	0.00000	0	0.00004	0.00001	0	0.00000
1008	1:1	4	504	asOF	0	0.00168	0	0.00203	0.00197	0	0.00144
1008	1:1	4	756	asOF	0	0.00938	0	0.01007	0.00992	0	0.00884
1008	1:1	4	1008	asOF	0	0.02487	0	0.02538	0.02518	0	0.02454
1008	2:1	2	504	asP	0	0.01584	0	0.01643	0.01622	0	0.01565
1008	2:1	2	1008	asP	0	0.02528	0	0.02587	0.02555	0	0.02497
1008	2:1	3	336	asP	0	0.01127	0	0.01247	0.01199	0	0.01115
1008	2:1	3	672	asP	0	0.01896	0	0.02050	0.01989	0	0.01874
1008	2:1	3	1008	asP	0	0.02479	0	0.02645	0.02578	0	0.02462
1008	2:1	4	252	asP	0	0.00856	0	0.00990	0.00924	0	0.00821
1008	2:1	4	504	asP	0	0.01542	0	0.01695	0.01613	0	0.01496
1008	2:1	4	756	asP	0	0.02042	0	0.02212	0.02129	0	0.01993
1008	2:1	4	1008	asP	0	0.02472	0	0.02645	0.02561	0	0.02433
1008	2:1	2	504	asOF	0	0.00160	0	0.00210	0.00199	0	0.00149
1008	2:1	2	1008	asOF	0	0.02499	0	0.02560	0.02534	0	0.02500
1008	2:1	3	336	asOF	0	0.00007	0	0.00026	0.00021	0	0.00009
1008	2:1	3	672	asOF	0	0.00598	0	0.00692	0.00677	0	0.00584
1008	2:1	3	1008	asOF	0	0.02501	0	0.02570	0.02542	0	0.02491
1008	2:1	4	252	asOF	0	0.00000	0	0.00004	0.00004	0	0.00001
1008	2:1	4	504	asOF	0	0.00159	0	0.00210	0.00199	0	0.00149
1008	2:1	4	756	asOF	0	0.00957	0	0.01050	0.01034	0	0.00937
1008	2:1	4	1008	asOF	0	0.02478	0	0.02567	0.02537	0	0.02477

Table 26: Ordinal distribution with $\alpha_1 = 1, \beta_1 = 1, \alpha_2 = 3, \beta_2 = 3$

N_K	AR	K	N_k	f	NA	WMW	NA	BM	BM-T	NA	LWO
144	1:1	2	72	asP	0	0.01729	0	0.019670000	0.017590000	0	0.01293
144	1:1	2	144	asP	0	0.02865	0	0.030030000	0.027510000	0	0.02303
144	1:1	3	48	asP	0	0.01239	1	0.017520175	0.014390144	0	0.00799
144	1:1	3	96	asP	0	0.02110	1	0.026500265	0.022740227	0	0.01519
144	1:1	3	144	asP	0	0.02858	1	0.032740327	0.028610286	0	0.02158
144	1:1	4	36	asP	0	0.00933	25	0.016454114	0.012473118	0	0.00450
144	1:1	4	72	asP	0	0.01700	25	0.025216304	0.020195049	0	0.01017
144	1:1	4	108	asP	0	0.02264	25	0.030977744	0.025716429	0	0.01496
144	1:1	4	144	asP	0	0.02806	25	0.035358840	0.030017504	0	0.01992
144	1:1	2	72	asOF	0	0.00158	0	0.005080000	0.004080000	0	0.00037
144	1:1	2	144	asOF	0	0.02883	0	0.028930000	0.027110000	0	0.02426
144	1:1	3	48	asOF	0	0.00008	1	0.002510025	0.001690017	0	0.00000
144	1:1	3	96	asOF	0	0.00621	1	0.011700117	0.010210102	0	0.00342
144	1:1	3	144	asOF	0	0.02852	1	0.030020300	0.028180282	0	0.02384
144	1:1	4	36	asOF	0	0.00000	22	0.001470323	0.000770169	0	0.00000
144	1:1	4	72	asOF	0	0.00158	22	0.006041329	0.004480986	0	0.00037
144	1:1	4	108	asOF	0	0.00977	22	0.016413611	0.014173118	0	0.00590
144	1:1	4	144	asOF	0	0.02855	22	0.031086839	0.028626298	0	0.02348
144	2:1	2	72	asP	0	0.00935	0	0.019420000	0.017720000	0	0.01285
144	2:1	2	144	asP	0	0.01615	0	0.029530000	0.027640000	0	0.02295
144	2:1	3	48	asP	0	0.00634	0	0.016840000	0.014130000	0	0.00795
144	2:1	3	96	asP	0	0.01127	0	0.026080000	0.022850000	0	0.01518
144	2:1	3	144	asP	0	0.01554	0	0.032210000	0.028830000	0	0.02160
144	2:1	4	36	asP	0	0.00466	11	0.015851744	0.012581384	0	0.00529
144	2:1	4	72	asP	0	0.00869	11	0.024182660	0.020122213	0	0.01133
144	2:1	4	108	asP	0	0.01198	11	0.030403344	0.026102871	0	0.01647
144	2:1	4	144	asP	0	0.01512	11	0.034853834	0.030313334	0	0.02106
144	2:1	2	72	asOF	0	0.00042	0	0.004730000	0.003870000	0	0.00030
144	2:1	2	144	asOF	0	0.01752	0	0.028240000	0.026810000	0	0.02391
144	2:1	3	48	asOF	0	0.00002	0	0.002130000	0.001370000	0	0.00000
144	2:1	3	96	asOF	0	0.00262	0	0.011270000	0.009700000	0	0.00348
144	2:1	3	144	asOF	0	0.01723	0	0.029610000	0.027650000	0	0.02350
144	2:1	4	36	asOF	0	0.00000	10	0.001540154	0.000900090	0	0.00000
144	2:1	4	72	asOF	0	0.00042	10	0.005760576	0.004470447	0	0.00030
144	2:1	4	108	asOF	0	0.00492	10	0.016221622	0.014641464	0	0.00685
144	2:1	4	144	asOF	0	0.01707	10	0.030563056	0.028512851	0	0.02330

Table 27: Ordinal distribution with $\alpha_1 = 1, \beta_1 = 1, \alpha_2 = 3, \beta_2 = 3$

N_K	AR	K	N_k	f	NA	WMW	NA	BM	BM-T	NA	LWO
288	1:1	2	144	asP	0	0.01779	0	0.01799	0.01690	0	0.01443
288	1:1	2	288	asP	0	0.02854	0	0.02748	0.02613	0	0.02366
288	1:1	3	96	asP	0	0.01242	0	0.01385	0.01243	0	0.00939
288	1:1	3	192	asP	0	0.02115	0	0.02215	0.02037	0	0.01669
288	1:1	3	288	asP	0	0.02833	0	0.02817	0.02611	0	0.02255
288	1:1	4	72	asP	0	0.00979	0	0.01201	0.01046	0	0.00700
288	1:1	4	144	asP	0	0.01750	0	0.01977	0.01771	0	0.01330
288	1:1	4	216	asP	0	0.02351	0	0.02535	0.02321	0	0.01856
288	1:1	4	288	asP	0	0.02861	0	0.02945	0.02719	0	0.02277
288	1:1	2	144	asOF	0	0.00157	0	0.00306	0.00270	0	0.00084
288	1:1	2	288	asOF	0	0.02845	0	0.02673	0.02575	0	0.02443
288	1:1	3	96	asOF	0	0.00008	0	0.00085	0.00063	0	0.00000
288	1:1	3	192	asOF	0	0.00637	0	0.00846	0.00792	0	0.00484
288	1:1	3	288	asOF	0	0.02841	0	0.02703	0.02615	0	0.02426
288	1:1	4	72	asOF	0	0.00000	0	0.00038	0.00026	0	0.00000
288	1:1	4	144	asOF	0	0.00157	0	0.00333	0.00289	0	0.00083
288	1:1	4	216	asOF	0	0.01048	0	0.01294	0.01214	0	0.00823
288	1:1	4	288	asOF	0	0.02842	0	0.02757	0.02646	0	0.02388
288	2:1	2	144	asP	0	0.00961	0	0.01684	0.01600	0	0.01377
288	2:1	2	288	asP	0	0.01608	0	0.02641	0.02534	0	0.02305
288	2:1	3	96	asP	0	0.00623	0	0.01354	0.01216	0	0.00922
288	2:1	3	192	asP	0	0.01087	0	0.02166	0.02000	0	0.01647
288	2:1	3	288	asP	0	0.01516	0	0.02756	0.02583	0	0.02240
288	2:1	4	72	asP	0	0.00465	0	0.01187	0.01034	0	0.00671
288	2:1	4	144	asP	0	0.00869	0	0.01933	0.01750	0	0.01281
288	2:1	4	216	asP	0	0.01190	0	0.02434	0.02237	0	0.01730
288	2:1	4	288	asP	0	0.01500	0	0.02864	0.02663	0	0.02186
288	2:1	2	144	asOF	0	0.00045	0	0.00295	0.00281	0	0.00082
288	2:1	2	288	asOF	0	0.01683	0	0.02667	0.02609	0	0.02460
288	2:1	3	96	asOF	0	0.00004	0	0.00069	0.00045	0	0.00002
288	2:1	3	192	asOF	0	0.00254	0	0.00783	0.00725	0	0.00446
288	2:1	3	288	asOF	0	0.01631	0	0.02673	0.02591	0	0.02415
288	2:1	4	72	asOF	0	0.00000	0	0.00023	0.00015	0	0.00000
288	2:1	4	144	asOF	0	0.00045	0	0.00303	0.00286	0	0.00081
288	2:1	4	216	asOF	0	0.00451	0	0.01145	0.01102	0	0.00723
288	2:1	4	288	asOF	0	0.01604	0	0.02660	0.02591	0	0.02349

Table 28: Ordinal distribution with $\alpha_1 = 1, \beta_1 = 1, \alpha_2 = 3, \beta_2 = 3$

N_K	AR	K	N_k	f	NA	WMW	NA	BM	BM-T	NA	LWO
576	1:1	2	288	asP	0	0.01713	0	0.01613	0.01554	0	0.01428
576	1:1	2	576	asP	0	0.02826	0	0.02557	0.02491	0	0.02371
576	1:1	3	192	asP	0	0.01282	0	0.01273	0.01210	0	0.01047
576	1:1	3	384	asP	0	0.02154	0	0.02066	0.01991	0	0.01798
576	1:1	3	576	asP	0	0.02872	0	0.02662	0.02579	0	0.02392
576	1:1	4	144	asP	0	0.00999	0	0.01069	0.00980	0	0.00792
576	1:1	4	288	asP	0	0.01759	0	0.01784	0.01678	0	0.01450
576	1:1	4	432	asP	0	0.02353	0	0.02325	0.02203	0	0.01954
576	1:1	4	576	asP	0	0.02900	0	0.02768	0.02641	0	0.02399
576	1:1	2	288	asOF	0	0.00143	0	0.00207	0.00189	0	0.00099
576	1:1	2	576	asOF	0	0.02776	0	0.02520	0.02485	0	0.02411
576	1:1	3	192	asOF	0	0.00011	0	0.00029	0.00021	0	0.00002
576	1:1	3	384	asOF	0	0.00609	0	0.00694	0.00656	0	0.00516
576	1:1	3	576	asOF	0	0.02807	0	0.02578	0.02522	0	0.02422
576	1:1	4	144	asOF	0	0.00001	0	0.00004	0.00003	0	0.00000
576	1:1	4	288	asOF	0	0.00143	0	0.00207	0.00188	0	0.00099
576	1:1	4	432	asOF	0	0.00990	0	0.01084	0.01050	0	0.00857
576	1:1	4	576	asOF	0	0.02787	0	0.02577	0.02540	0	0.02416
576	2:1	2	288	asP	0	0.01007	0	0.01662	0.01626	0	0.01507
576	2:1	2	576	asP	0	0.01626	0	0.02570	0.02522	0	0.02407
576	2:1	3	192	asP	0	0.00702	0	0.01300	0.01246	0	0.01084
576	2:1	3	384	asP	0	0.01161	0	0.02060	0.01998	0	0.01793
576	2:1	3	576	asP	0	0.01552	0	0.02645	0.02581	0	0.02386
576	2:1	4	144	asP	0	0.00523	0	0.01053	0.00992	0	0.00817
576	2:1	4	288	asP	0	0.00922	0	0.01782	0.01696	0	0.01462
576	2:1	4	432	asP	0	0.01210	0	0.02289	0.02190	0	0.01950
576	2:1	4	576	asP	0	0.01519	0	0.02713	0.02608	0	0.02376
576	2:1	2	288	asOF	0	0.00041	0	0.00219	0.00202	0	0.00112
576	2:1	2	576	asOF	0	0.01657	0	0.02495	0.02463	0	0.02387
576	2:1	3	192	asOF	0	0.00002	0	0.00027	0.00022	0	0.00005
576	2:1	3	384	asOF	0	0.00277	0	0.00655	0.00632	0	0.00493
576	2:1	3	576	asOF	0	0.01638	0	0.02499	0.02471	0	0.02387
576	2:1	4	144	asOF	0	0.00000	0	0.00009	0.00004	0	0.00000
576	2:1	4	288	asOF	0	0.00041	0	0.00223	0.00205	0	0.00112
576	2:1	4	432	asOF	0	0.00423	0	0.01054	0.01022	0	0.00829
576	2:1	4	576	asOF	0	0.01613	0	0.02558	0.02521	0	0.02390

Table 29: Ordinal distribution with $\alpha_1 = 1, \beta_1 = 1, \alpha_2 = 3, \beta_2 = 3$

N_K	AR	K	N_k	f	NA	WMW	NA	BM	BM-T	NA	LWO
864	1:1	2	432	asP	0	0.01681	0	0.01555	0.01514	0	0.01432
864	1:1	2	864	asP	0	0.02779	0	0.02515	0.02473	0	0.02396
864	1:1	3	288	asP	0	0.01241	0	0.01196	0.01141	0	0.01042
864	1:1	3	576	asP	0	0.02115	0	0.01986	0.01933	0	0.01827
864	1:1	3	864	asP	0	0.02824	0	0.02595	0.02535	0	0.02434
864	1:1	4	216	asP	0	0.00986	0	0.00975	0.00929	0	0.00796
864	1:1	4	432	asP	0	0.01717	0	0.01639	0.01576	0	0.01414
864	1:1	4	648	asP	0	0.02327	0	0.02185	0.02110	0	0.01931
864	1:1	4	864	asP	0	0.02844	0	0.02625	0.02545	0	0.02374
864	1:1	2	432	asOF	0	0.00157	0	0.00199	0.00186	0	0.00118
864	1:1	2	864	asOF	0	0.02797	0	0.02502	0.02479	0	0.02436
864	1:1	3	288	asOF	0	0.00009	0	0.00020	0.00018	0	0.00004
864	1:1	3	576	asOF	0	0.00633	0	0.00669	0.00650	0	0.00551
864	1:1	3	864	asOF	0	0.02801	0	0.02540	0.02517	0	0.02451
864	1:1	4	216	asOF	0	0.00001	0	0.00004	0.00003	0	0.00000
864	1:1	4	432	asOF	0	0.00157	0	0.00200	0.00188	0	0.00118
864	1:1	4	648	asOF	0	0.01015	0	0.01073	0.01049	0	0.00921
864	1:1	4	864	asOF	0	0.02792	0	0.02564	0.02529	0	0.02454
864	2:1	2	432	asP	0	0.01012	0	0.01675	0.01645	0	0.01575
864	2:1	2	864	asP	0	0.01629	0	0.02598	0.02562	0	0.02490
864	2:1	3	288	asP	0	0.00693	0	0.01239	0.01190	0	0.01069
864	2:1	3	576	asP	0	0.01195	0	0.02034	0.01972	0	0.01836
864	2:1	3	864	asP	0	0.01574	0	0.02620	0.02558	0	0.02428
864	2:1	4	216	asP	0	0.00505	0	0.01014	0.00951	0	0.00843
864	2:1	4	432	asP	0	0.00912	0	0.01712	0.01639	0	0.01492
864	2:1	4	648	asP	0	0.01253	0	0.02255	0.02183	0	0.02028
864	2:1	4	864	asP	0	0.01513	0	0.02655	0.02579	0	0.02425
864	2:1	2	432	asOF	0	0.00067	0	0.00211	0.00203	0	0.00135
864	2:1	2	864	asOF	0	0.01691	0	0.02523	0.02489	0	0.02431
864	2:1	3	288	asOF	0	0.00001	0	0.00031	0.00030	0	0.00004
864	2:1	3	576	asOF	0	0.00291	0	0.00715	0.00696	0	0.00590
864	2:1	3	864	asOF	0	0.01668	0	0.02521	0.02493	0	0.02420
864	2:1	4	216	asOF	0	0.00000	0	0.00008	0.00006	0	0.00000
864	2:1	4	432	asOF	0	0.00066	0	0.00212	0.00205	0	0.00135
864	2:1	4	648	asOF	0	0.00509	0	0.01081	0.01064	0	0.00943
864	2:1	4	864	asOF	0	0.01650	0	0.02528	0.02503	0	0.02419

Table 30: Ordinal distribution with $\alpha_1 = 1, \beta_1 = 1, \alpha_2 = 3, \beta_2 = 3$

N_K	AR	K	N_k	f	NA	WMW	NA	BM	BM-T	NA	LWO
1008	1:1	2	504	asP	0	0.01694	0	0.01553	0.01521	0	0.01462
1008	1:1	2	1008	asP	0	0.02834	0	0.02541	0.02495	0	0.02441
1008	1:1	3	336	asP	0	0.01233	0	0.01165	0.01142	0	0.01059
1008	1:1	3	672	asP	0	0.02048	0	0.01892	0.01855	0	0.01764
1008	1:1	3	1008	asP	0	0.02788	0	0.02511	0.02466	0	0.02376
1008	1:1	4	252	asP	0	0.01020	0	0.00999	0.00940	0	0.00838
1008	1:1	4	504	asP	0	0.01719	0	0.01632	0.01567	0	0.01431
1008	1:1	4	756	asP	0	0.02311	0	0.02165	0.02096	0	0.01937
1008	1:1	4	1008	asP	0	0.02849	0	0.02584	0.02517	0	0.02364
1008	1:1	2	504	asOF	0	0.00146	0	0.00184	0.00172	0	0.00119
1008	1:1	2	1008	asOF	0	0.02812	0	0.02540	0.02518	0	0.02474
1008	1:1	3	336	asOF	0	0.00012	0	0.00018	0.00015	0	0.00010
1008	1:1	3	672	asOF	0	0.00601	0	0.00627	0.00611	0	0.00538
1008	1:1	3	1008	asOF	0	0.02800	0	0.02529	0.02503	0	0.02460
1008	1:1	4	252	asOF	0	0.00003	0	0.00009	0.00008	0	0.00003
1008	1:1	4	504	asOF	0	0.00147	0	0.00187	0.00174	0	0.00119
1008	1:1	4	756	asOF	0	0.00981	0	0.01017	0.00999	0	0.00878
1008	1:1	4	1008	asOF	0	0.02797	0	0.02532	0.02506	0	0.02425
1008	2:1	2	504	asP	0	0.00934	0	0.01558	0.01535	0	0.01467
1008	2:1	2	1008	asP	0	0.01596	0	0.02524	0.02498	0	0.02431
1008	2:1	3	336	asP	0	0.00679	0	0.01207	0.01177	0	0.01096
1008	2:1	3	672	asP	0	0.01141	0	0.01989	0.01956	0	0.01847
1008	2:1	3	1008	asP	0	0.01568	0	0.02617	0.02580	0	0.02474
1008	2:1	4	252	asP	0	0.00541	0	0.01004	0.00971	0	0.00852
1008	2:1	4	504	asP	0	0.00934	0	0.01643	0.01595	0	0.01455
1008	2:1	4	756	asP	0	0.01239	0	0.02135	0.02082	0	0.01934
1008	2:1	4	1008	asP	0	0.01550	0	0.02584	0.02529	0	0.02390
1008	2:1	2	504	asOF	0	0.00057	0	0.00194	0.00186	0	0.00142
1008	2:1	2	1008	asOF	0	0.01754	0	0.02553	0.02541	0	0.02507
1008	2:1	3	336	asOF	0	0.00004	0	0.00019	0.00019	0	0.00011
1008	2:1	3	672	asOF	0	0.00273	0	0.00635	0.00623	0	0.00551
1008	2:1	3	1008	asOF	0	0.01733	0	0.02573	0.02552	0	0.02511
1008	2:1	4	252	asOF	0	0.00000	0	0.00004	0.00004	0	0.00001
1008	2:1	4	504	asOF	0	0.00057	0	0.00195	0.00186	0	0.00142
1008	2:1	4	756	asOF	0	0.00478	0	0.00996	0.00977	0	0.00884
1008	2:1	4	1008	asOF	0	0.01691	0	0.02549	0.02524	0	0.02471

Table 31: Poisson distribution with $\lambda_1 = 2, \lambda_2 = 2$

N_K	AR	K	N_k	f	NA	WMW	NA	BM	BM-T	NA	LWO
144	1:1	2	72	asP	0	0.01518	0	0.019520000	0.017660000	0	0.01255
144	1:1	2	144	asP	0	0.02433	0	0.029340000	0.027030000	0	0.02190
144	1:1	3	48	asP	0	0.01133	1	0.017120171	0.014420144	0	0.00822
144	1:1	3	96	asP	0	0.01912	1	0.025900259	0.022830228	0	0.01549
144	1:1	3	144	asP	0	0.02487	1	0.031660317	0.028550286	0	0.02155
144	1:1	4	36	asP	0	0.00869	17	0.016122741	0.012992209	0	0.00516
144	1:1	4	72	asP	0	0.01508	17	0.024384145	0.020383465	0	0.01092
144	1:1	4	108	asP	0	0.02058	17	0.030595201	0.026464499	0	0.01601
144	1:1	4	144	asP	0	0.02447	17	0.034565876	0.030305152	0	0.02030
144	1:1	2	72	asOF	0	0.00155	0	0.005010000	0.004170000	0	0.00035
144	1:1	2	144	asOF	0	0.02528	0	0.028600000	0.027380000	0	0.02414
144	1:1	3	48	asOF	0	0.00005	1	0.002340023	0.001630016	0	0.00000
144	1:1	3	96	asOF	0	0.00603	1	0.011280113	0.009930099	0	0.00345
144	1:1	3	144	asOF	0	0.02545	1	0.029820298	0.028190282	0	0.02373
144	1:1	4	36	asOF	0	0.00001	17	0.001480252	0.000880150	0	0.00000
144	1:1	4	72	asOF	0	0.00155	17	0.005921007	0.004740806	0	0.00035
144	1:1	4	108	asOF	0	0.00995	17	0.016302771	0.014552474	0	0.00641
144	1:1	4	144	asOF	0	0.02526	17	0.030665213	0.028524849	0	0.02330
144	2:1	2	72	asP	0	0.01450	1	0.019290193	0.016800168	0	0.01208
144	2:1	2	144	asP	0	0.02384	1	0.029240292	0.026220262	0	0.02165
144	2:1	3	48	asP	0	0.00998	8	0.017441395	0.013951116	0	0.00771
144	2:1	3	96	asP	0	0.01784	8	0.026552124	0.022481799	0	0.01495
144	2:1	3	144	asP	0	0.02373	8	0.032622610	0.028312265	0	0.02117
144	2:1	4	36	asP	0	0.00817	102	0.017267613	0.013143406	0	0.00543
144	2:1	4	72	asP	0	0.01443	102	0.025526037	0.020400809	0	0.01127
144	2:1	4	108	asP	0	0.01950	102	0.031542173	0.026046567	0	0.01608
144	2:1	4	144	asP	0	0.02378	102	0.035846563	0.030070672	0	0.02067
144	2:1	2	72	asOF	0	0.00131	1	0.005340053	0.004250043	0	0.00059
144	2:1	2	144	asOF	0	0.02386	1	0.027450275	0.025820258	0	0.02295
144	2:1	3	48	asOF	0	0.00003	7	0.002640185	0.001810127	0	0.00001
144	2:1	3	96	asOF	0	0.00555	7	0.012260858	0.010490734	0	0.00368
144	2:1	3	144	asOF	0	0.02375	7	0.029392057	0.027081896	0	0.02270
144	2:1	4	36	asOF	0	0.00000	94	0.002342202	0.001391308	0	0.00000
144	2:1	4	72	asOF	0	0.00131	94	0.006936520	0.005104799	0	0.00059
144	2:1	4	108	asOF	0	0.00921	94	0.017366324	0.014763878	0	0.00664
144	2:1	4	144	asOF	0	0.02392	94	0.030848998	0.028066382	0	0.02278

Table 32: Poisson distribution with $\lambda_1 = 2, \lambda_2 = 2$

N_K	AR	K	N_k	f	NA	WMW	NA	BM	BM-T	NA	LWO
288	1:1	2	144	asP	0	0.01575	0	0.01764	0.01677	0	0.01435
288	1:1	2	288	asP	0	0.02527	0	0.02744	0.02642	0	0.02387
288	1:1	3	96	asP	0	0.01085	0	0.01366	0.01247	0	0.00926
288	1:1	3	192	asP	0	0.01881	0	0.02214	0.02079	0	0.01690
288	1:1	3	288	asP	0	0.02485	0	0.02827	0.02687	0	0.02310
288	1:1	4	72	asP	0	0.00791	0	0.01148	0.00993	0	0.00612
288	1:1	4	144	asP	0	0.01453	0	0.01916	0.01718	0	0.01236
288	1:1	4	216	asP	0	0.02013	0	0.02499	0.02290	0	0.01781
288	1:1	4	288	asP	0	0.02440	0	0.02924	0.02707	0	0.02219
288	1:1	2	144	asOF	0	0.00143	0	0.00294	0.00261	0	0.00070
288	1:1	2	288	asOF	0	0.02473	0	0.02616	0.02560	0	0.02410
288	1:1	3	96	asOF	0	0.00010	0	0.00062	0.00053	0	0.00000
288	1:1	3	192	asOF	0	0.00596	0	0.00805	0.00780	0	0.00481
288	1:1	3	288	asOF	0	0.02476	0	0.02665	0.02603	0	0.02391
288	1:1	4	72	asOF	0	0.00002	0	0.00037	0.00026	0	0.00000
288	1:1	4	144	asOF	0	0.00142	0	0.00310	0.00271	0	0.00070
288	1:1	4	216	asOF	0	0.00972	0	0.01256	0.01182	0	0.00789
288	1:1	4	288	asOF	0	0.02472	0	0.02696	0.02609	0	0.02353
288	2:1	2	144	asP	0	0.01562	0	0.01814	0.01694	0	0.01448
288	2:1	2	288	asP	0	0.02552	0	0.02813	0.02680	0	0.02440
288	2:1	3	96	asP	0	0.01069	0	0.01411	0.01246	0	0.00942
288	2:1	3	192	asP	0	0.01896	0	0.02327	0.02116	0	0.01734
288	2:1	3	288	asP	0	0.02535	0	0.02973	0.02736	0	0.02372
288	2:1	4	72	asP	0	0.00869	0	0.01289	0.01100	0	0.00739
288	2:1	4	144	asP	0	0.01564	0	0.02085	0.01845	0	0.01387
288	2:1	4	216	asP	0	0.02083	0	0.02667	0.02403	0	0.01904
288	2:1	4	288	asP	0	0.02560	0	0.03127	0.02852	0	0.02384
288	2:1	2	144	asOF	0	0.00149	0	0.00361	0.00298	0	0.00090
288	2:1	2	288	asOF	0	0.02587	0	0.02778	0.02670	0	0.02504
288	2:1	3	96	asOF	0	0.00007	0	0.00101	0.00078	0	0.00003
288	2:1	3	192	asOF	0	0.00630	0	0.00933	0.00868	0	0.00508
288	2:1	3	288	asOF	0	0.02587	0	0.02812	0.02709	0	0.02485
288	2:1	4	72	asOF	0	0.00000	0	0.00045	0.00023	0	0.00000
288	2:1	4	144	asOF	0	0.00149	0	0.00392	0.00313	0	0.00090
288	2:1	4	216	asOF	0	0.00990	0	0.01367	0.01257	0	0.00850
288	2:1	4	288	asOF	0	0.02558	0	0.02853	0.02720	0	0.02466

Table 33: Poisson distribution with $\lambda_1 = 2, \lambda_2 = 2$

N_K	AR	K	N_k	f	NA	WMW	NA	BM	BM-T	NA	LWO
576	1:1	2	288	asP	0	0.01563	0	0.01669	0.01621	0	0.01501
576	1:1	2	576	asP	0	0.02526	0	0.02652	0.02591	0	0.02472
576	1:1	3	192	asP	0	0.01122	0	0.01252	0.01206	0	0.01039
576	1:1	3	384	asP	0	0.01869	0	0.02043	0.01973	0	0.01775
576	1:1	3	576	asP	0	0.02477	0	0.02655	0.02579	0	0.02391
576	1:1	4	144	asP	0	0.00878	0	0.01056	0.00992	0	0.00784
576	1:1	4	288	asP	0	0.01545	0	0.01774	0.01688	0	0.01431
576	1:1	4	432	asP	0	0.02072	0	0.02313	0.02223	0	0.01943
576	1:1	4	576	asP	0	0.02531	0	0.02770	0.02677	0	0.02405
576	1:1	2	288	asOF	0	0.00157	0	0.00224	0.00210	0	0.00112
576	1:1	2	576	asOF	0	0.02497	0	0.02557	0.02528	0	0.02462
576	1:1	3	192	asOF	0	0.00012	0	0.00036	0.00031	0	0.00006
576	1:1	3	384	asOF	0	0.00572	0	0.00683	0.00661	0	0.00503
576	1:1	3	576	asOF	0	0.02483	0	0.02586	0.02553	0	0.02433
576	1:1	4	144	asOF	0	0.00001	0	0.00012	0.00008	0	0.00000
576	1:1	4	288	asOF	0	0.00157	0	0.00231	0.00216	0	0.00112
576	1:1	4	432	asOF	0	0.00946	0	0.01073	0.01046	0	0.00845
576	1:1	4	576	asOF	0	0.02529	0	0.02619	0.02587	0	0.02473
576	2:1	2	288	asP	0	0.01562	0	0.01666	0.01599	0	0.01499
576	2:1	2	576	asP	0	0.02508	0	0.02636	0.02546	0	0.02443
576	2:1	3	192	asP	0	0.01074	0	0.01252	0.01179	0	0.01012
576	2:1	3	384	asP	0	0.01837	0	0.02037	0.01936	0	0.01747
576	2:1	3	576	asP	0	0.02432	0	0.02647	0.02529	0	0.02343
576	2:1	4	144	asP	0	0.00816	0	0.01025	0.00920	0	0.00752
576	2:1	4	288	asP	0	0.01506	0	0.01791	0.01656	0	0.01430
576	2:1	4	432	asP	0	0.02019	0	0.02329	0.02177	0	0.01928
576	2:1	4	576	asP	0	0.02445	0	0.02754	0.02592	0	0.02360
576	2:1	2	288	asOF	0	0.00130	0	0.00236	0.00213	0	0.00105
576	2:1	2	576	asOF	0	0.02523	0	0.02618	0.02569	0	0.02495
576	2:1	3	192	asOF	0	0.00009	0	0.00039	0.00031	0	0.00005
576	2:1	3	384	asOF	0	0.00592	0	0.00719	0.00686	0	0.00522
576	2:1	3	576	asOF	0	0.02520	0	0.02614	0.02568	0	0.02462
576	2:1	4	144	asOF	0	0.00000	0	0.00008	0.00006	0	0.00000
576	2:1	4	288	asOF	0	0.00129	0	0.00240	0.00214	0	0.00104
576	2:1	4	432	asOF	0	0.00951	0	0.01103	0.01055	0	0.00856
576	2:1	4	576	asOF	0	0.02504	0	0.02617	0.02559	0	0.02438

Table 34: Poisson distribution with $\lambda_1 = 2, \lambda_2 = 2$

N_K	AR	K	N_k	f	NA	WMW	NA	BM	BM-T	NA	LWO
864	1:1	2	432	asP	0	0.01590	0	0.01653	0.01630	0	0.01550
864	1:1	2	864	asP	0	0.02523	0	0.02597	0.02563	0	0.02483
864	1:1	3	288	asP	0	0.01136	0	0.01219	0.01186	0	0.01083
864	1:1	3	576	asP	0	0.01928	0	0.02031	0.01989	0	0.01866
864	1:1	3	864	asP	0	0.02496	0	0.02594	0.02551	0	0.02438
864	1:1	4	216	asP	0	0.00904	0	0.01025	0.00975	0	0.00847
864	1:1	4	432	asP	0	0.01616	0	0.01780	0.01714	0	0.01543
864	1:1	4	648	asP	0	0.02112	0	0.02283	0.02219	0	0.02028
864	1:1	4	864	asP	0	0.02517	0	0.02690	0.02622	0	0.02437
864	1:1	2	432	asOF	0	0.00155	0	0.00210	0.00198	0	0.00131
864	1:1	2	864	asOF	0	0.02471	0	0.02521	0.02501	0	0.02444
864	1:1	3	288	asOF	0	0.00011	0	0.00024	0.00021	0	0.00008
864	1:1	3	576	asOF	0	0.00588	0	0.00659	0.00645	0	0.00538
864	1:1	3	864	asOF	0	0.02461	0	0.02530	0.02508	0	0.02430
864	1:1	4	216	asOF	0	0.00000	0	0.00002	0.00001	0	0.00000
864	1:1	4	432	asOF	0	0.00155	0	0.00210	0.00198	0	0.00131
864	1:1	4	648	asOF	0	0.00932	0	0.01026	0.01010	0	0.00870
864	1:1	4	864	asOF	0	0.02452	0	0.02522	0.02498	0	0.02414
864	2:1	2	432	asP	0	0.01569	0	0.01665	0.01612	0	0.01517
864	2:1	2	864	asP	0	0.02504	0	0.02610	0.02552	0	0.02460
864	2:1	3	288	asP	0	0.01202	0	0.01326	0.01272	0	0.01150
864	2:1	3	576	asP	0	0.02010	0	0.02158	0.02099	0	0.01949
864	2:1	3	864	asP	0	0.02605	0	0.02751	0.02689	0	0.02545
864	2:1	4	216	asP	0	0.00915	0	0.01069	0.00998	0	0.00854
864	2:1	4	432	asP	0	0.01566	0	0.01754	0.01659	0	0.01493
864	2:1	4	648	asP	0	0.02114	0	0.02316	0.02211	0	0.02035
864	2:1	4	864	asP	0	0.02545	0	0.02738	0.02624	0	0.02463
864	2:1	2	432	asOF	0	0.00145	0	0.00195	0.00181	0	0.00115
864	2:1	2	864	asOF	0	0.02530	0	0.02589	0.02562	0	0.02510
864	2:1	3	288	asOF	0	0.00011	0	0.00035	0.00031	0	0.00006
864	2:1	3	576	asOF	0	0.00604	0	0.00689	0.00667	0	0.00569
864	2:1	3	864	asOF	0	0.02539	0	0.02605	0.02569	0	0.02503
864	2:1	4	216	asOF	0	0.00001	0	0.00011	0.00009	0	0.00000
864	2:1	4	432	asOF	0	0.00145	0	0.00199	0.00183	0	0.00115
864	2:1	4	648	asOF	0	0.01011	0	0.01115	0.01082	0	0.00926
864	2:1	4	864	asOF	0	0.02524	0	0.02616	0.02570	0	0.02479

Table 35: Poisson distribution with $\lambda_1 = 2, \lambda_2 = 2$

N_K	AR	K	N_k	f	NA	WMW	NA	BM	BM-T	NA	LWO
1008	1:1	2	504	asP	0	0.01528	0	0.01580	0.01557	0	0.01497
1008	1:1	2	1008	asP	0	0.02469	0	0.02529	0.02501	0	0.02435
1008	1:1	3	336	asP	0	0.01143	0	0.01215	0.01179	0	0.01104
1008	1:1	3	672	asP	0	0.01870	0	0.01964	0.01927	0	0.01823
1008	1:1	3	1008	asP	0	0.02485	0	0.02580	0.02540	0	0.02442
1008	1:1	4	252	asP	0	0.00921	0	0.01021	0.00978	0	0.00867
1008	1:1	4	504	asP	0	0.01602	0	0.01723	0.01670	0	0.01530
1008	1:1	4	756	asP	0	0.02082	0	0.02219	0.02162	0	0.02009
1008	1:1	4	1008	asP	0	0.02511	0	0.02643	0.02586	0	0.02441
1008	1:1	2	504	asOF	0	0.00146	0	0.00184	0.00178	0	0.00134
1008	1:1	2	1008	asOF	0	0.02379	0	0.02420	0.02401	0	0.02365
1008	1:1	3	336	asOF	0	0.00012	0	0.00025	0.00021	0	0.00006
1008	1:1	3	672	asOF	0	0.00537	0	0.00604	0.00590	0	0.00506
1008	1:1	3	1008	asOF	0	0.02377	0	0.02433	0.02415	0	0.02357
1008	1:1	4	252	asOF	0	0.00002	0	0.00005	0.00002	0	0.00000
1008	1:1	4	504	asOF	0	0.00147	0	0.00187	0.00179	0	0.00133
1008	1:1	4	756	asOF	0	0.00902	0	0.00973	0.00959	0	0.00851
1008	1:1	4	1008	asOF	0	0.02391	0	0.02439	0.02423	0	0.02369
1008	2:1	2	504	asP	0	0.01540	0	0.01612	0.01579	0	0.01505
1008	2:1	2	1008	asP	0	0.02489	0	0.02553	0.02514	0	0.02453
1008	2:1	3	336	asP	0	0.01115	0	0.01213	0.01163	0	0.01071
1008	2:1	3	672	asP	0	0.01880	0	0.01992	0.01931	0	0.01833
1008	2:1	3	1008	asP	0	0.02456	0	0.02562	0.02500	0	0.02405
1008	2:1	4	252	asP	0	0.00876	0	0.00975	0.00923	0	0.00805
1008	2:1	4	504	asP	0	0.01505	0	0.01644	0.01571	0	0.01424
1008	2:1	4	756	asP	0	0.02042	0	0.02189	0.02114	0	0.01952
1008	2:1	4	1008	asP	0	0.02442	0	0.02597	0.02520	0	0.02369
1008	2:1	2	504	asOF	0	0.00150	0	0.00201	0.00188	0	0.00133
1008	2:1	2	1008	asOF	0	0.02539	0	0.02591	0.02564	0	0.02522
1008	2:1	3	336	asOF	0	0.00006	0	0.00028	0.00020	0	0.00003
1008	2:1	3	672	asOF	0	0.00609	0	0.00683	0.00663	0	0.00557
1008	2:1	3	1008	asOF	0	0.02540	0	0.02587	0.02554	0	0.02496
1008	2:1	4	252	asOF	0	0.00001	0	0.00007	0.00007	0	0.00000
1008	2:1	4	504	asOF	0	0.00151	0	0.00204	0.00192	0	0.00133
1008	2:1	4	756	asOF	0	0.00971	0	0.01071	0.01037	0	0.00914
1008	2:1	4	1008	asOF	0	0.02544	0	0.02621	0.02588	0	0.02504

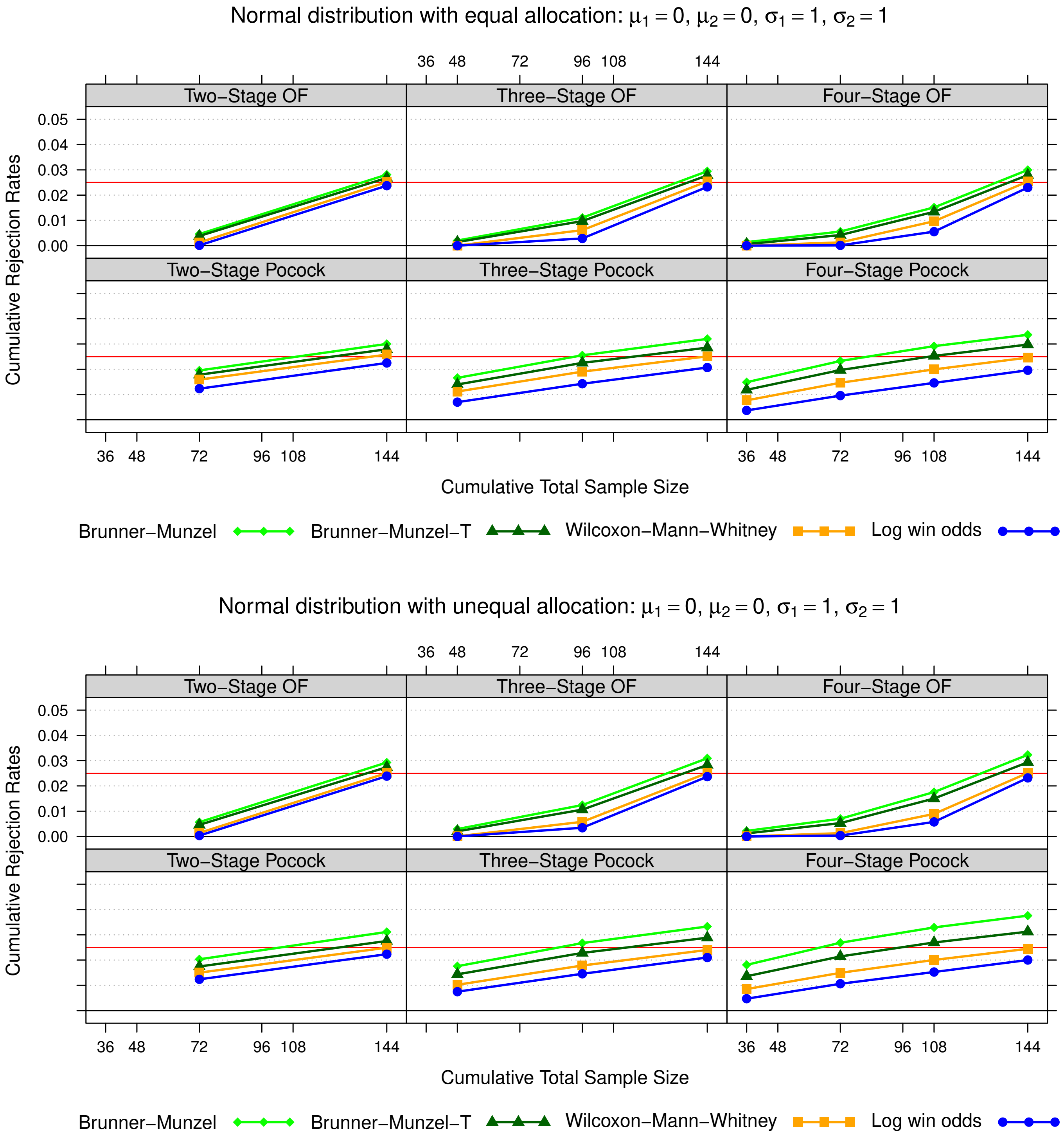

Figure 4: Normal distribution - Setting 1 with total maximum sample size 144

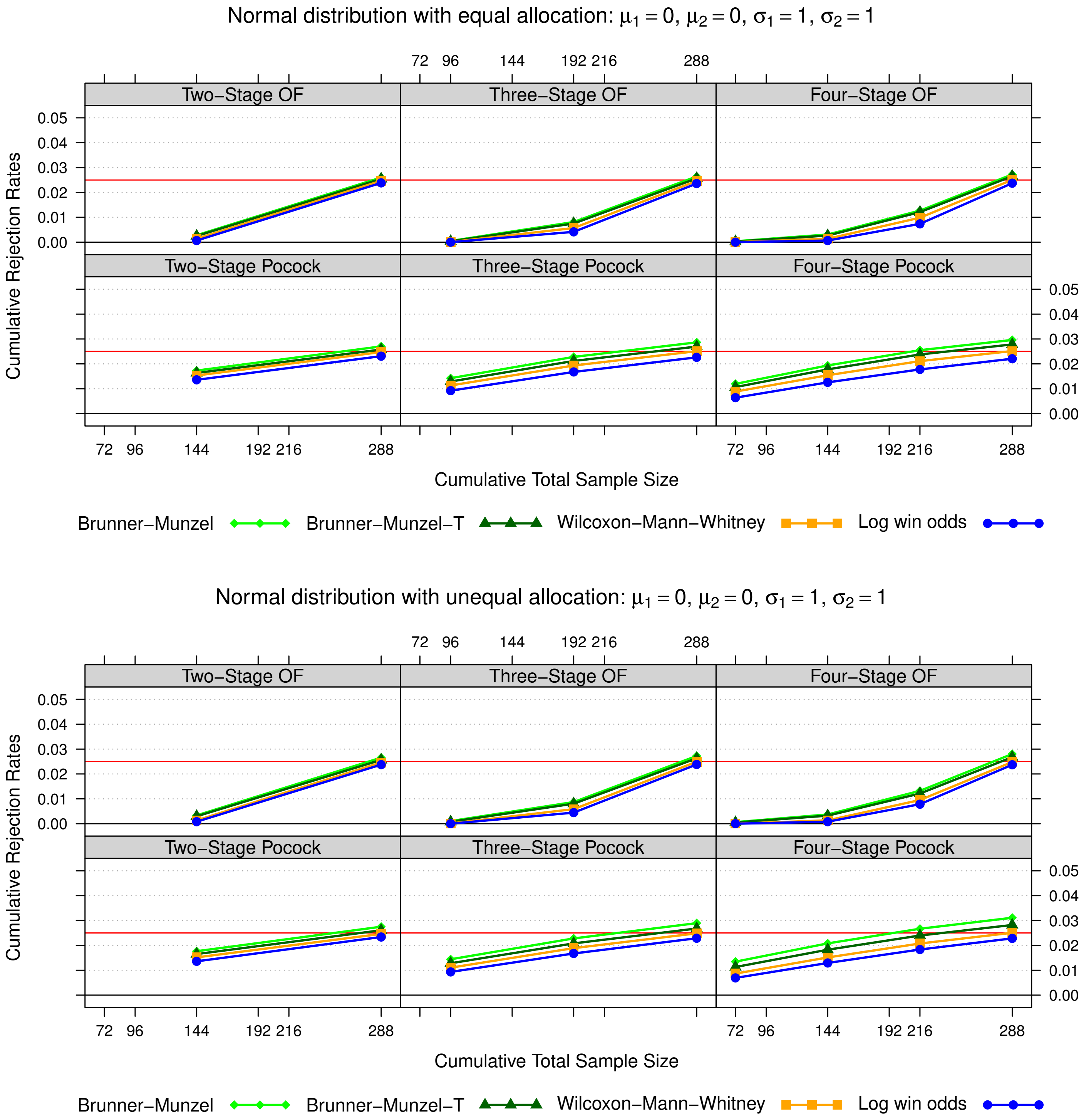

Figure 5: Normal distribution - Setting 1 with total maximum sample size 288

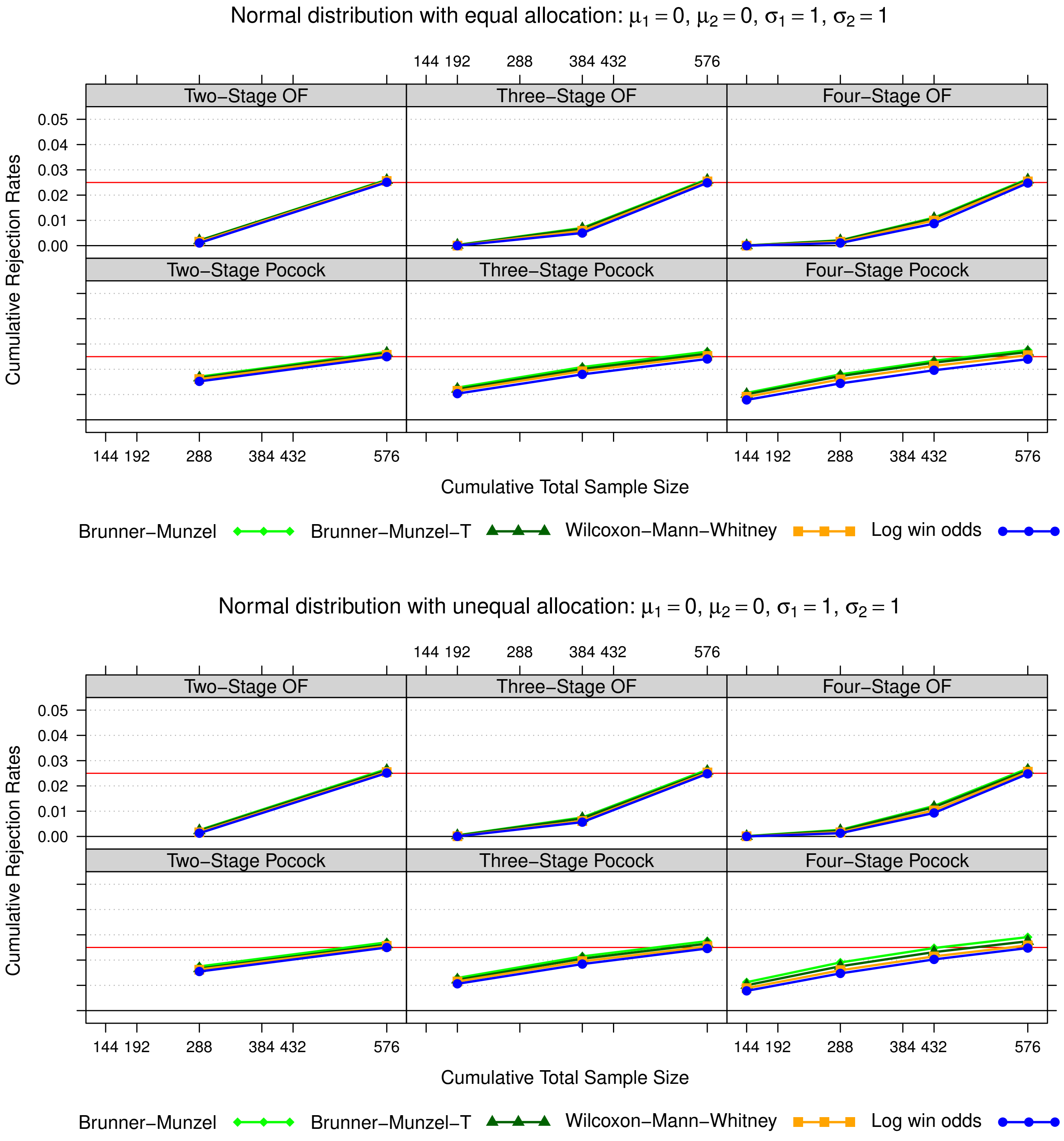

Figure 6: Normal distribution - Setting 1 with total maximum sample size 576

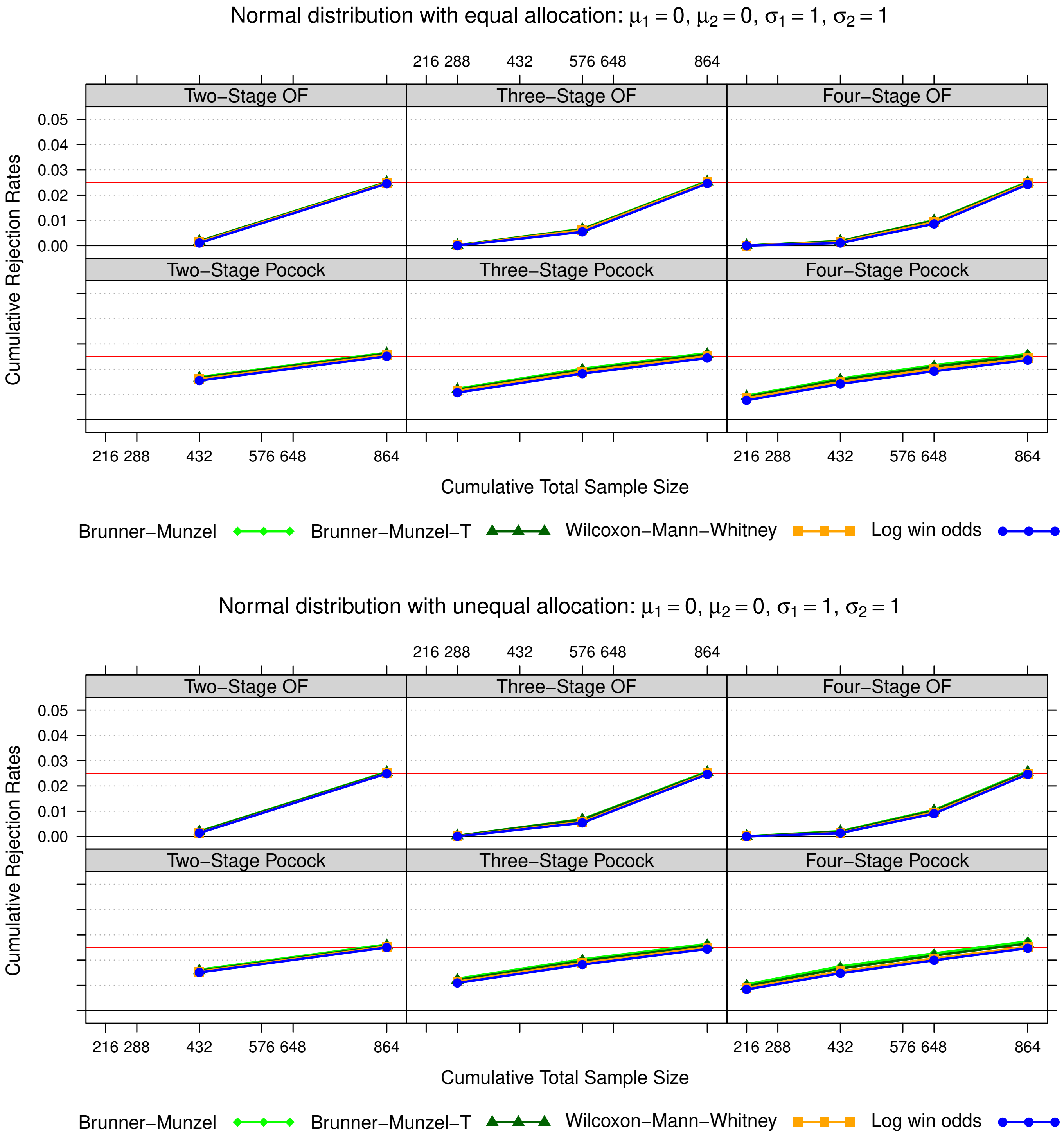

Figure 7: Normal distribution - Setting 1 with total maximum sample size 864

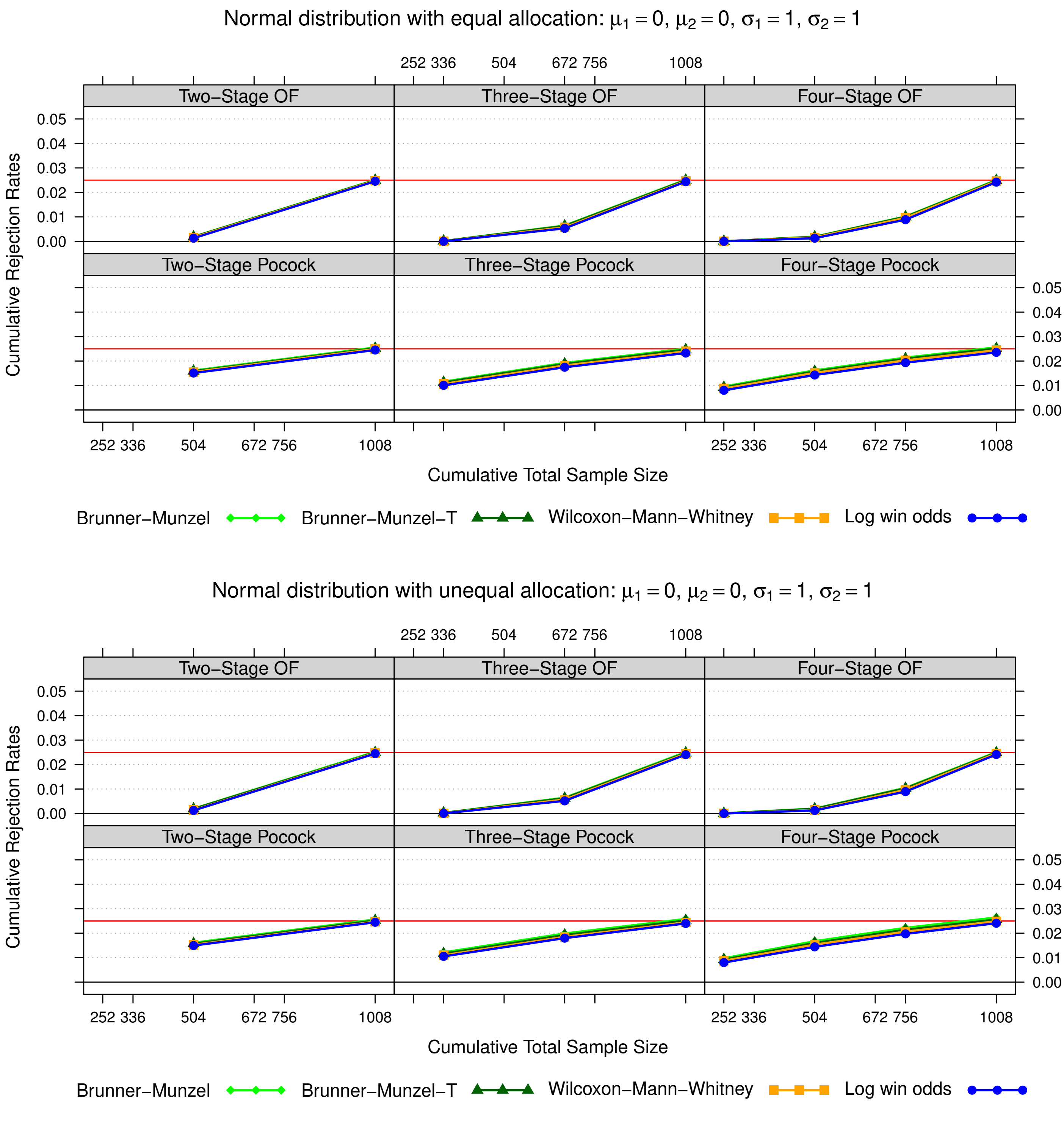

Figure 8: Normal distribution - Setting 1 with total maximum sample size 1008

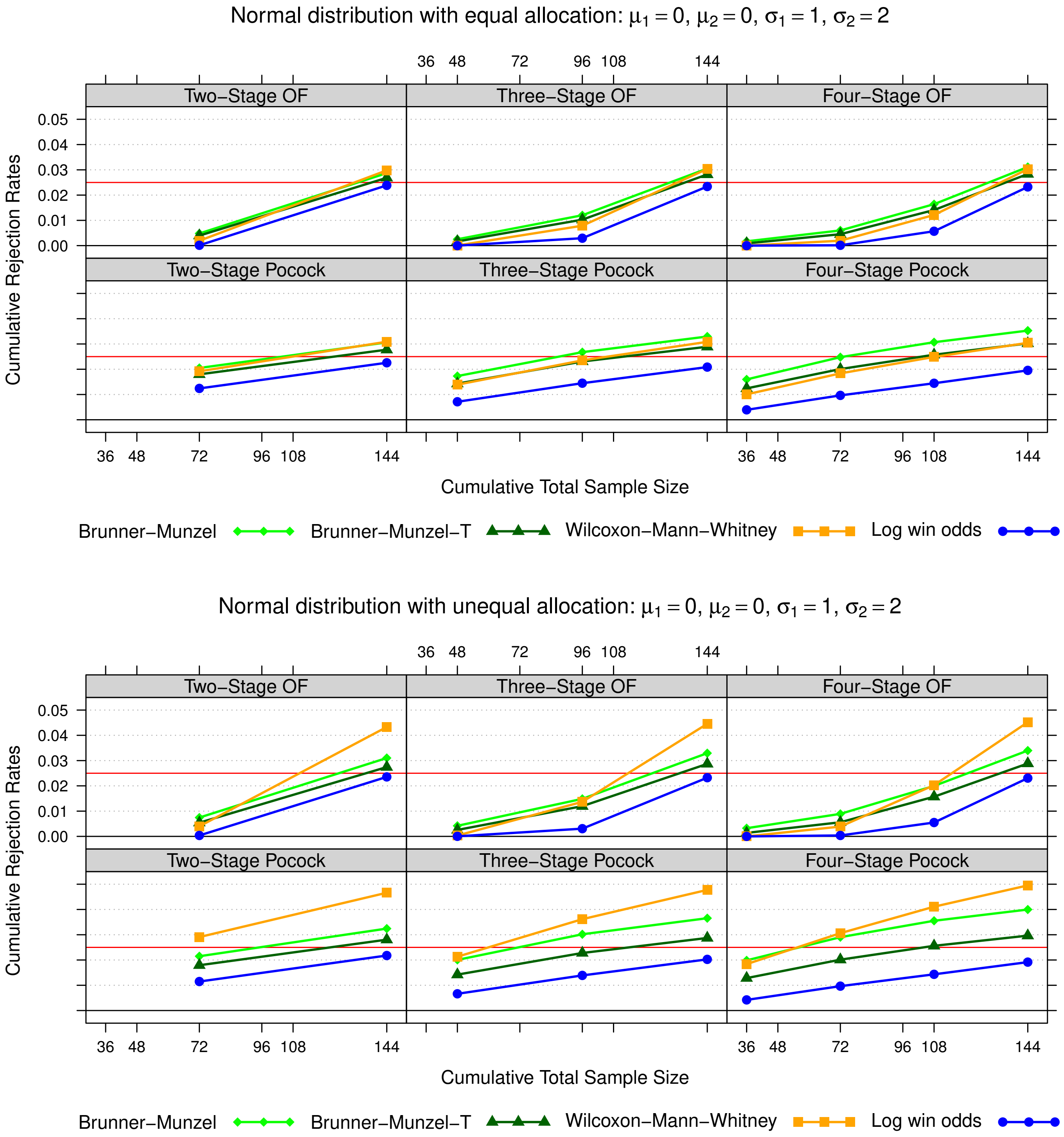

Figure 9: Normal distribution - Setting 2 with total maximum sample size 144

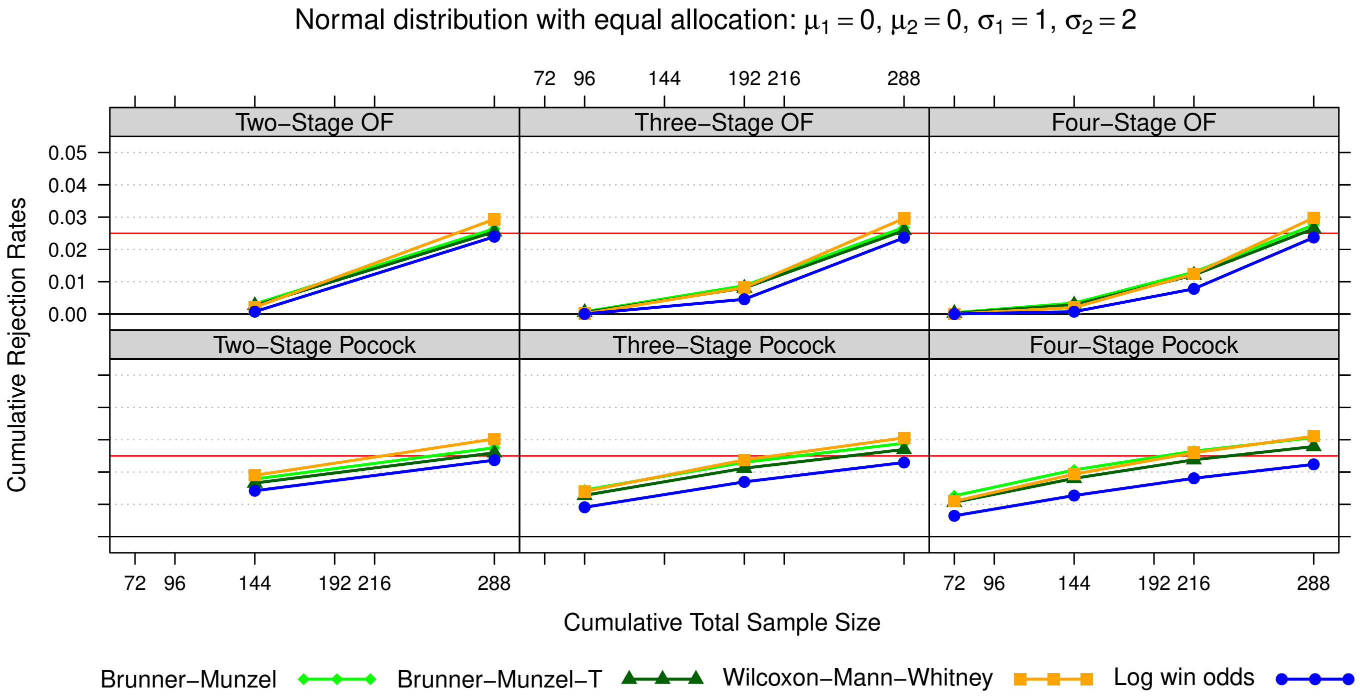

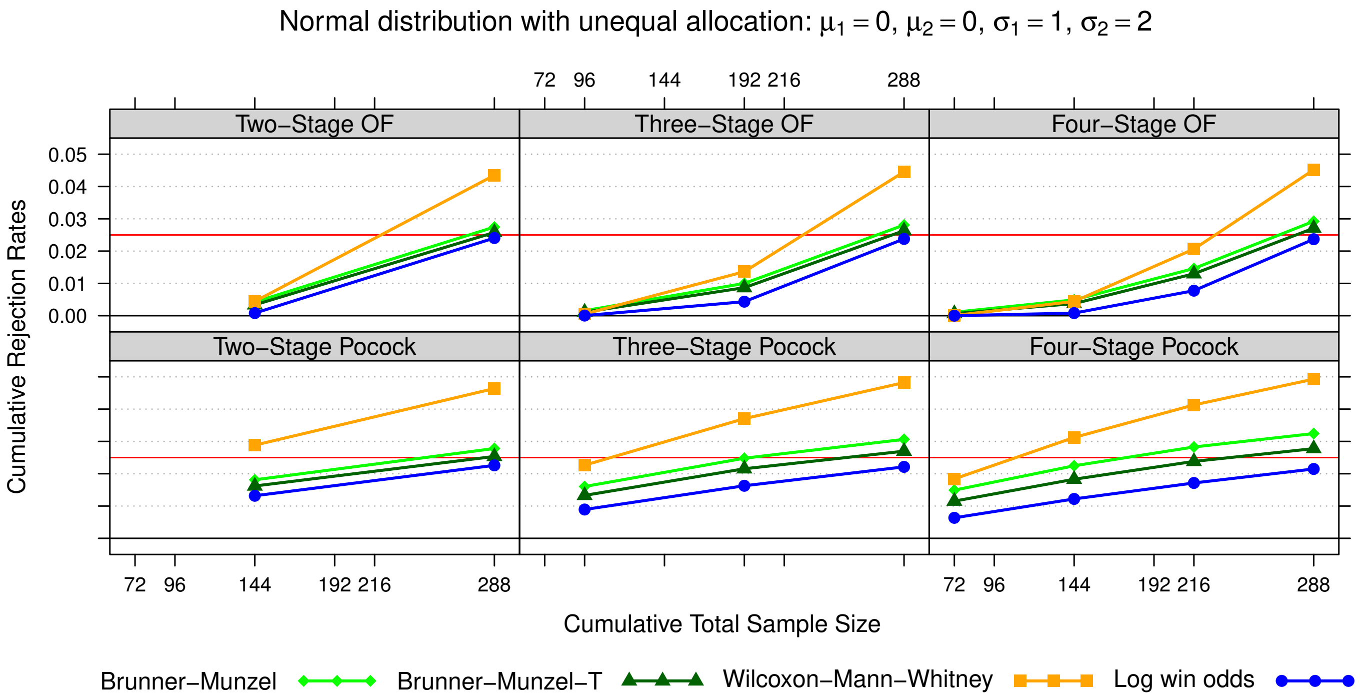

Figure 10: Normal distribution - Setting 2 with total maximum sample size 288

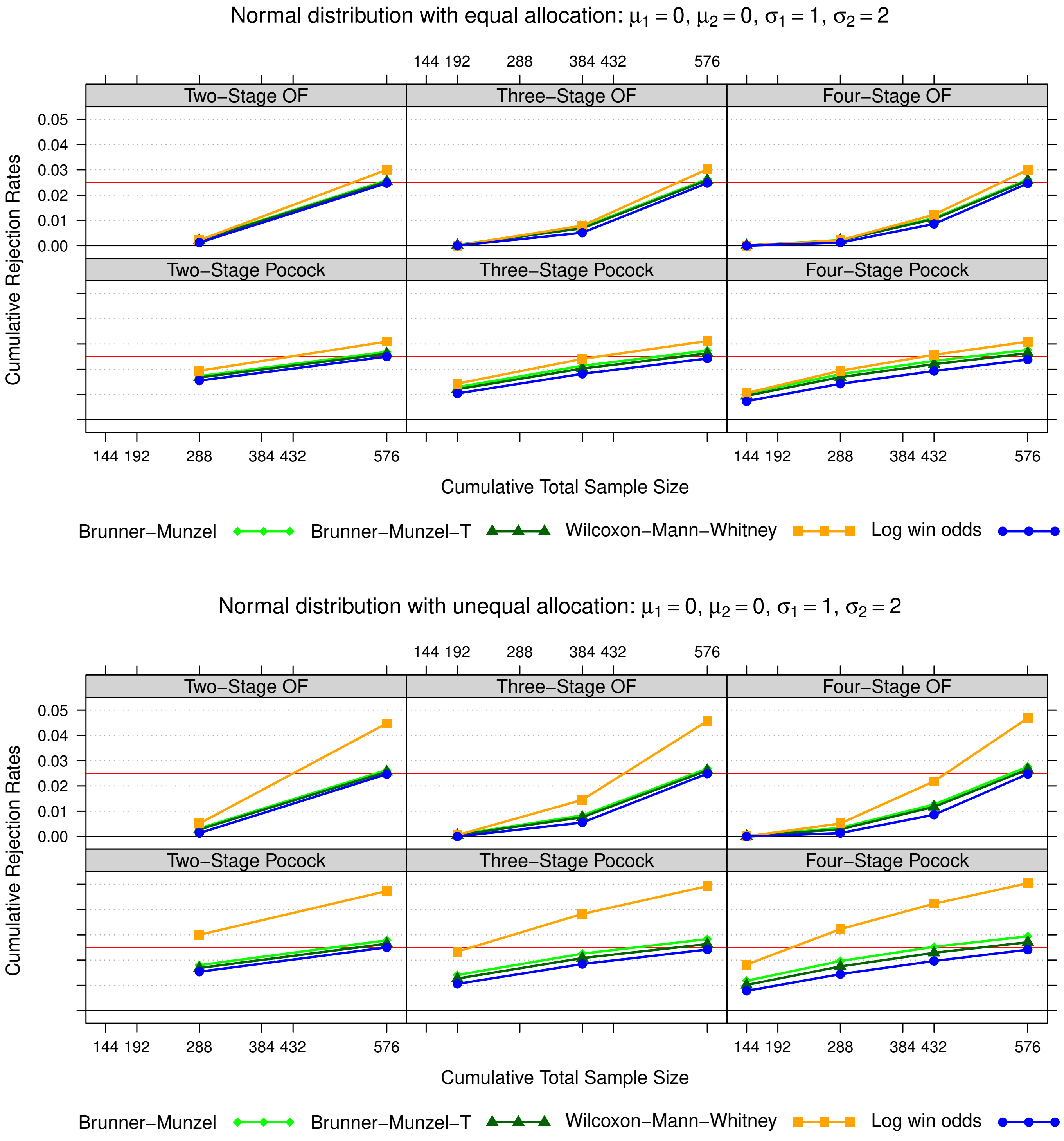

Figure 11: Normal distribution - Setting 2 with total maximum sample size 576

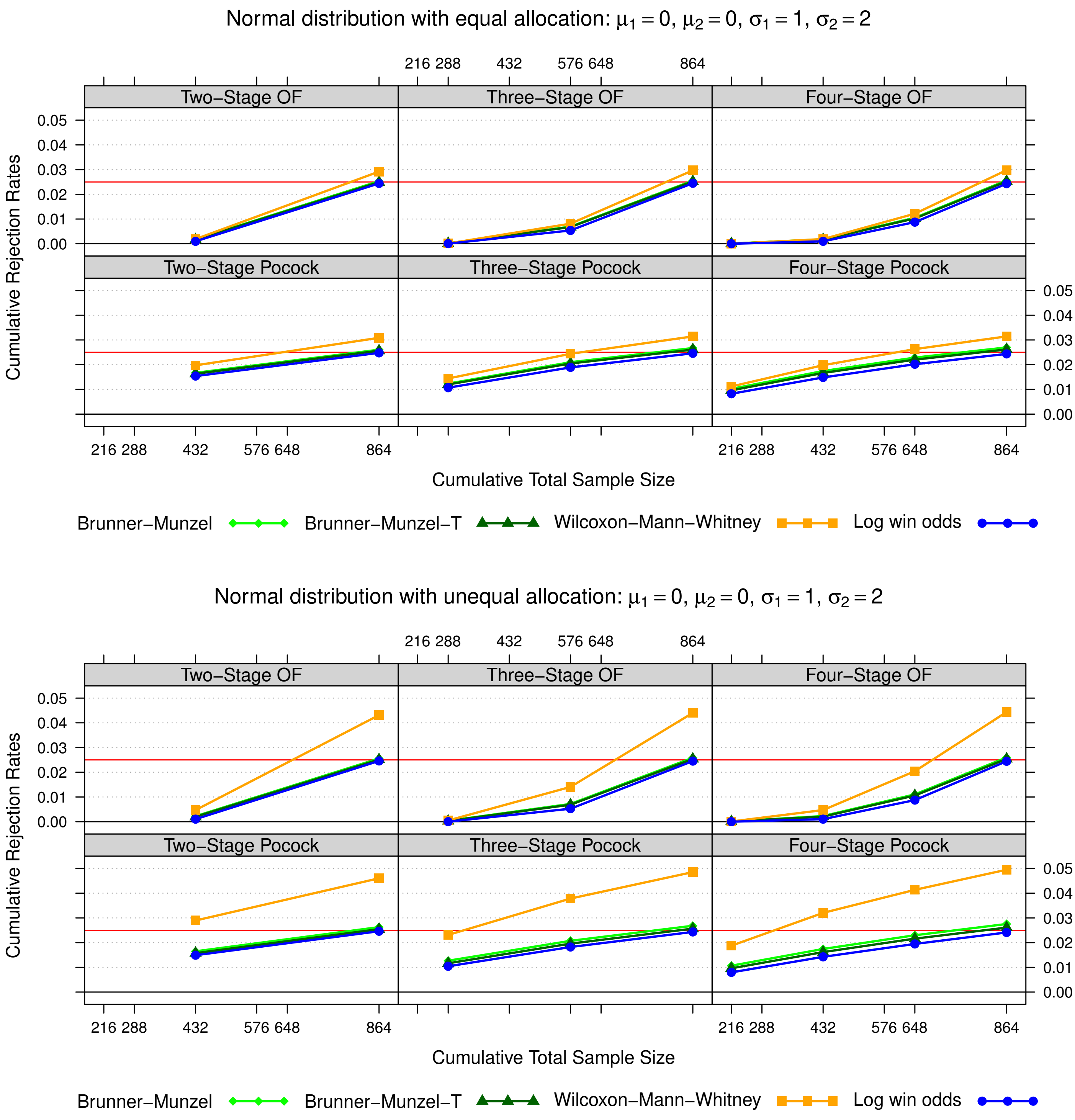

Figure 12: Normal distribution - Setting 2 with total maximum sample size 864

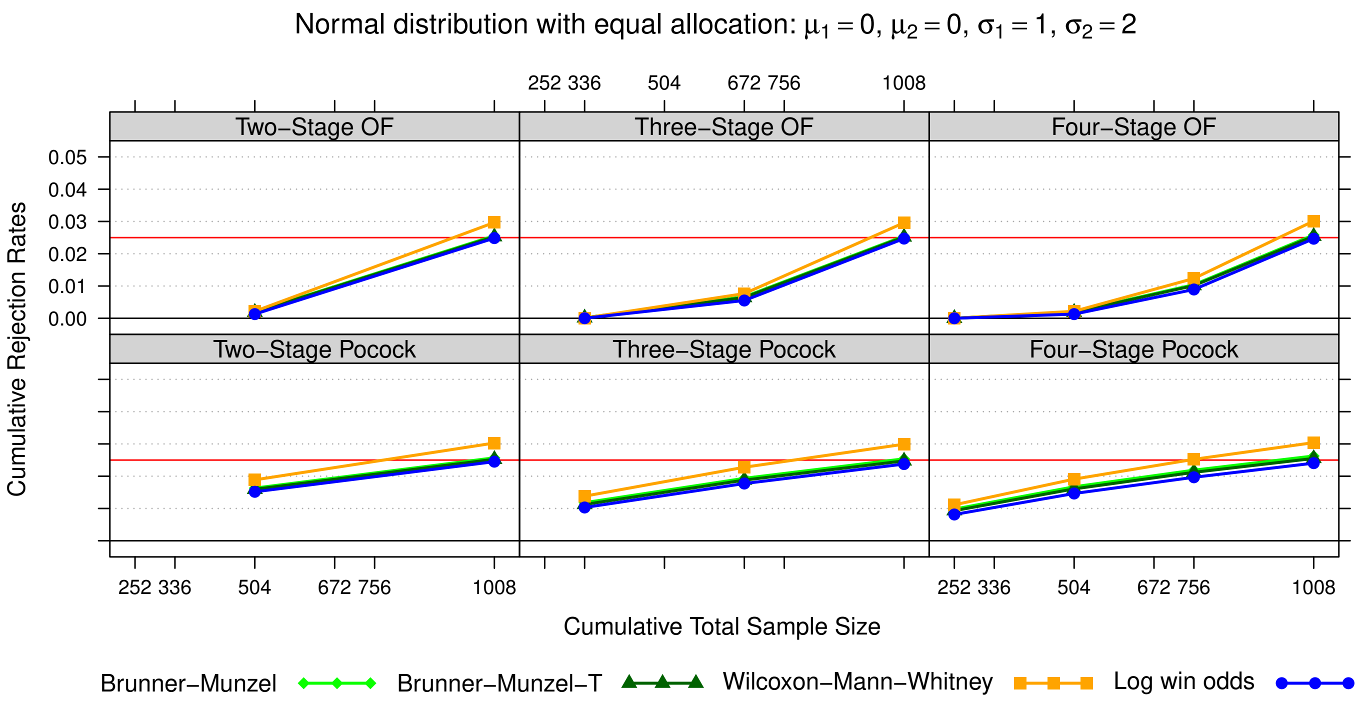

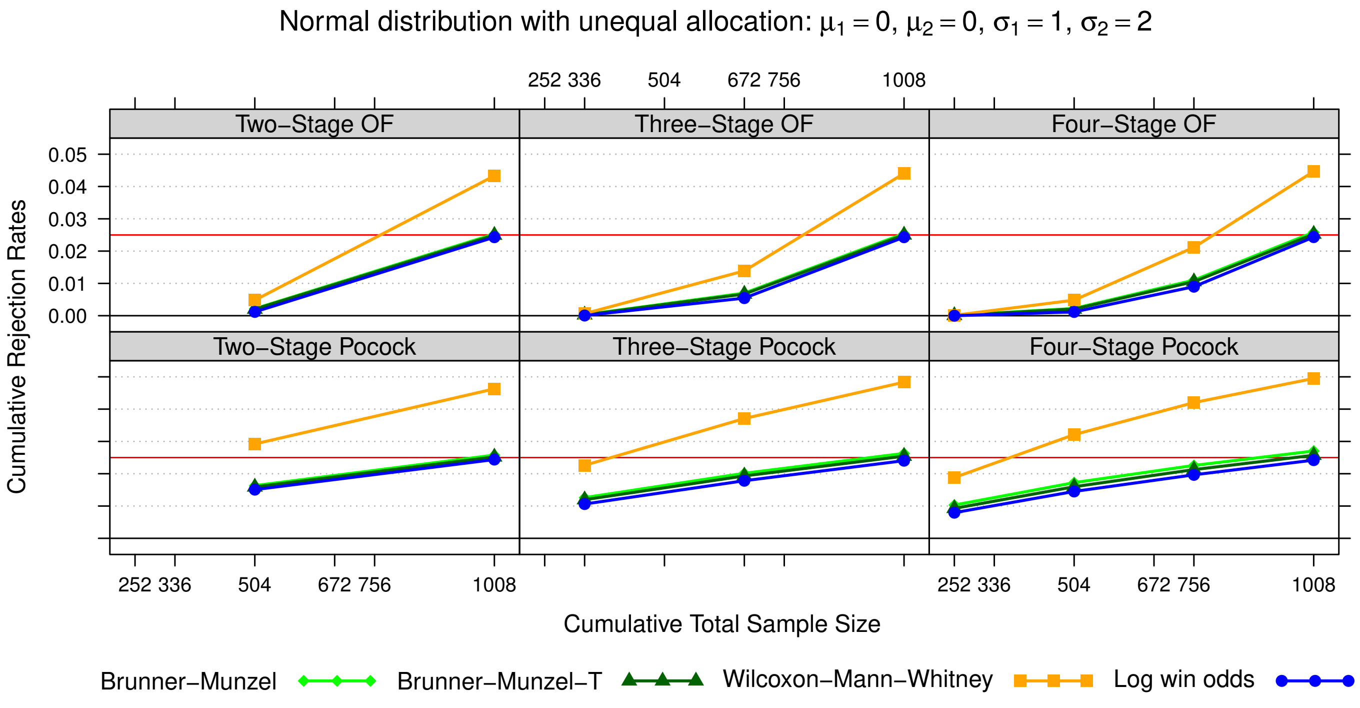

Figure 13: Normal distribution - Setting 2 with total maximum sample size 1008

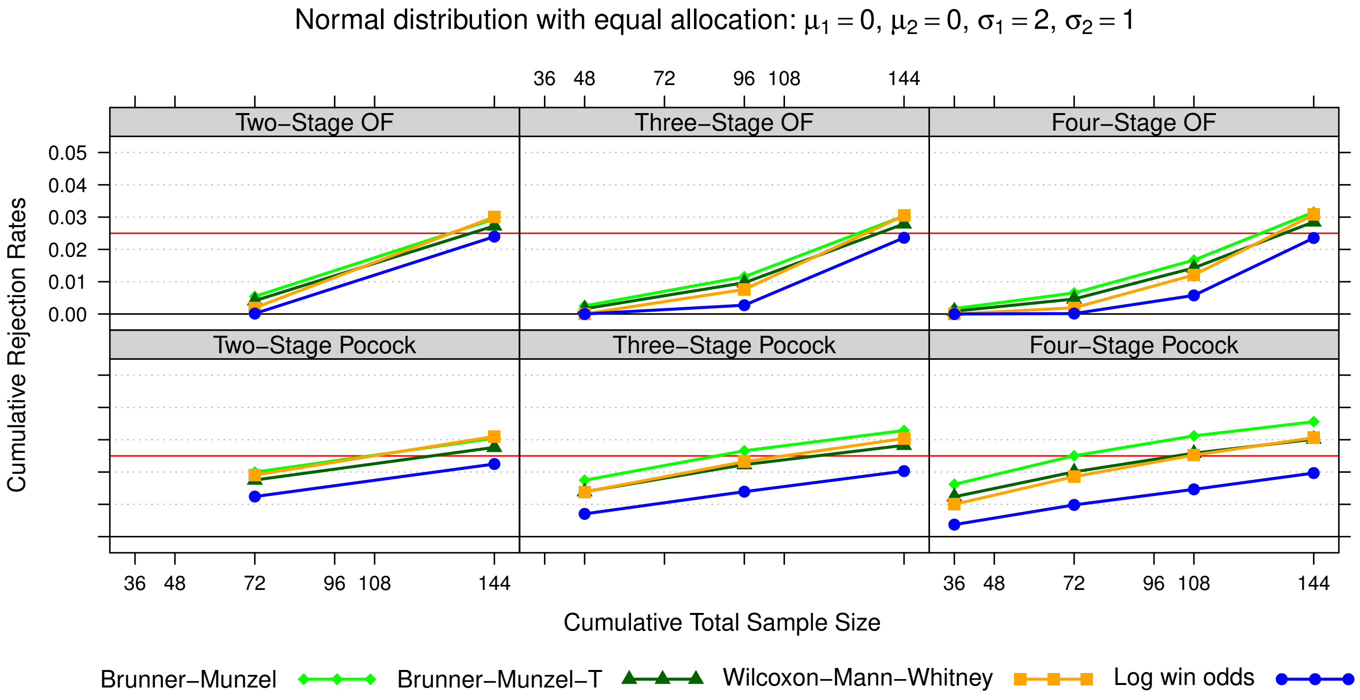

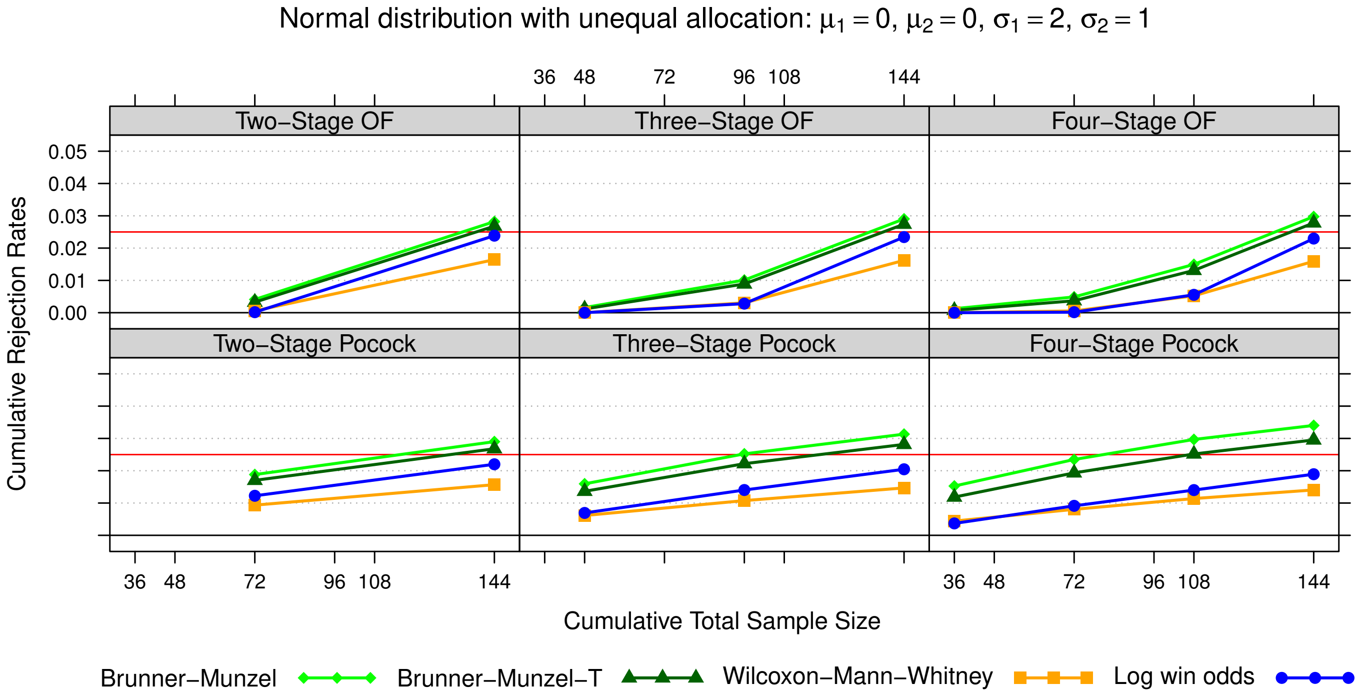

Figure 14: Normal distribution - Setting 3 with total maximum sample size 144

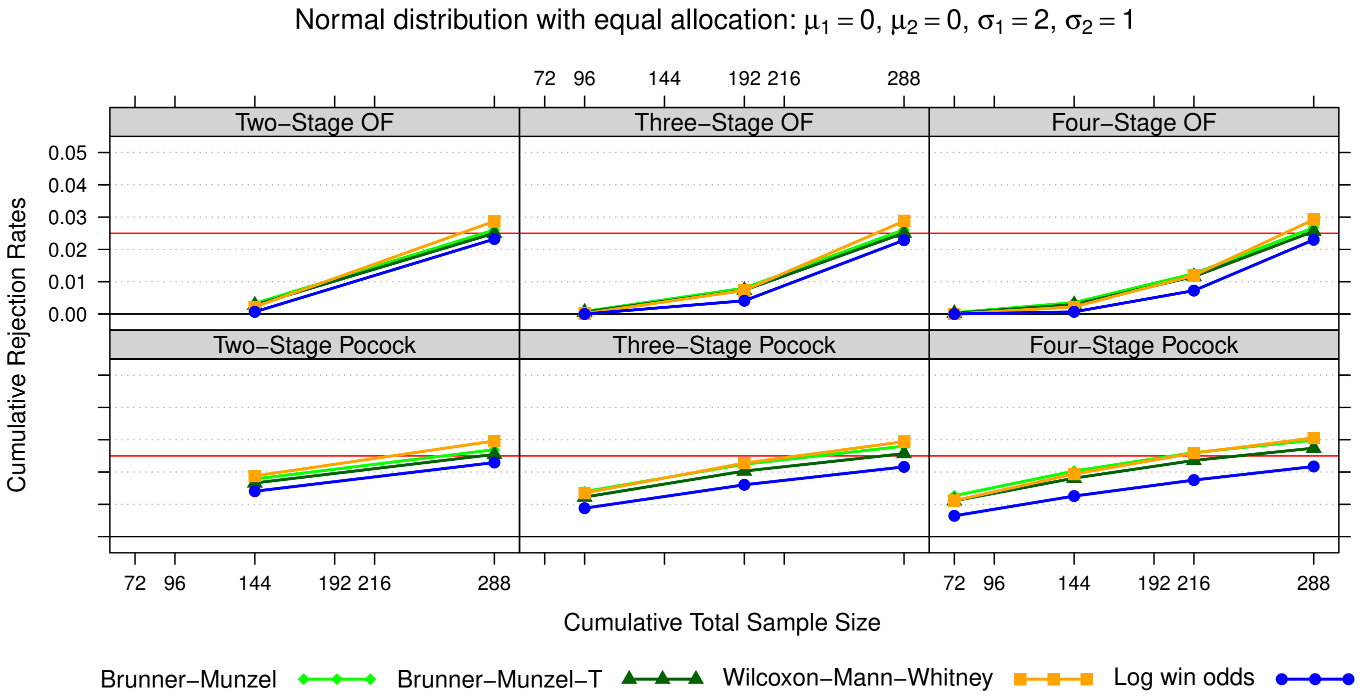

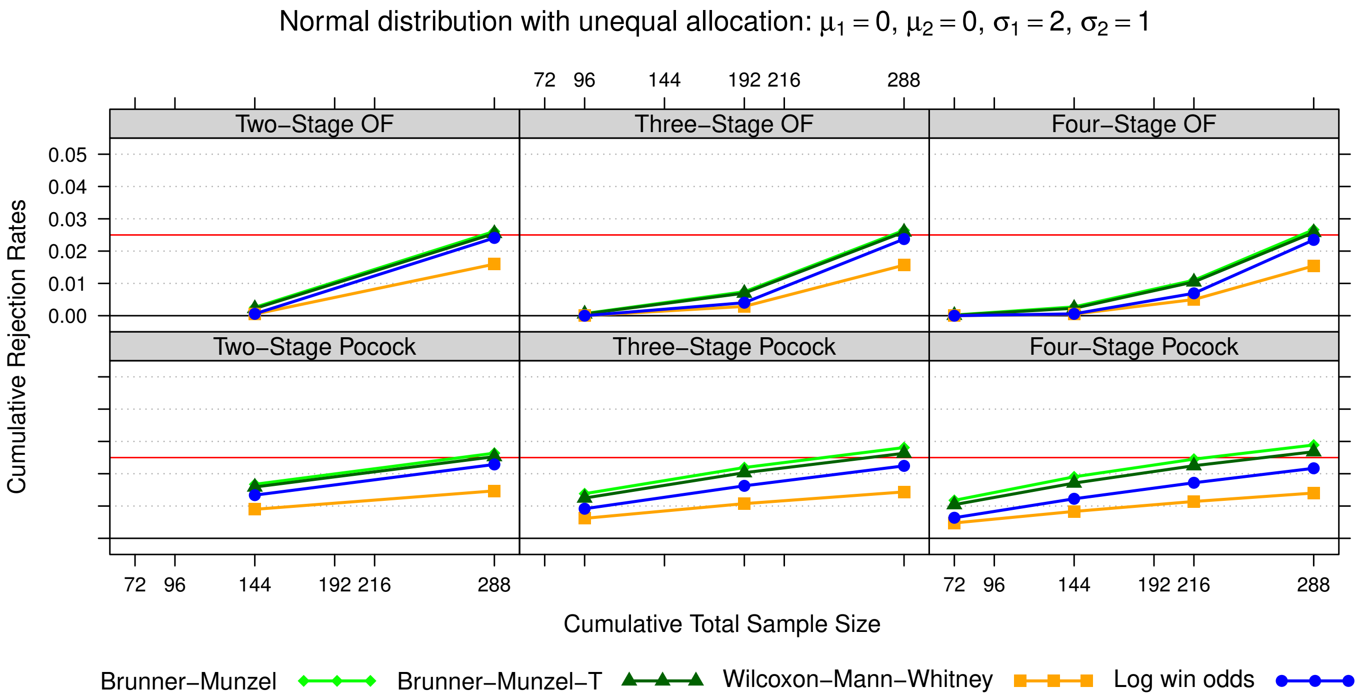

Figure 15: Normal distribution - Setting 3 with total maximum sample size 288

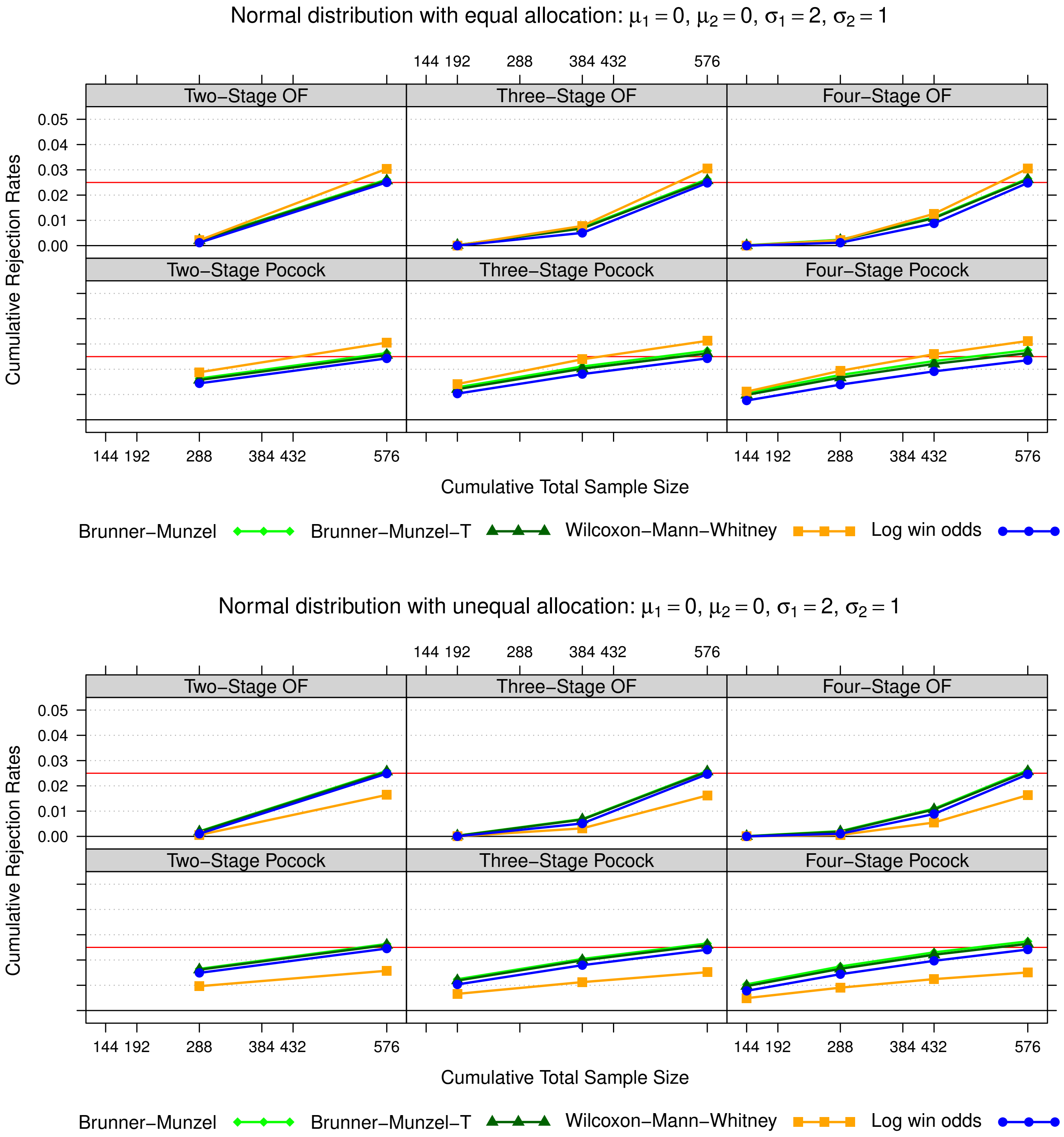

Figure 16: Normal distribution - Setting 3 with total maximum sample size 576

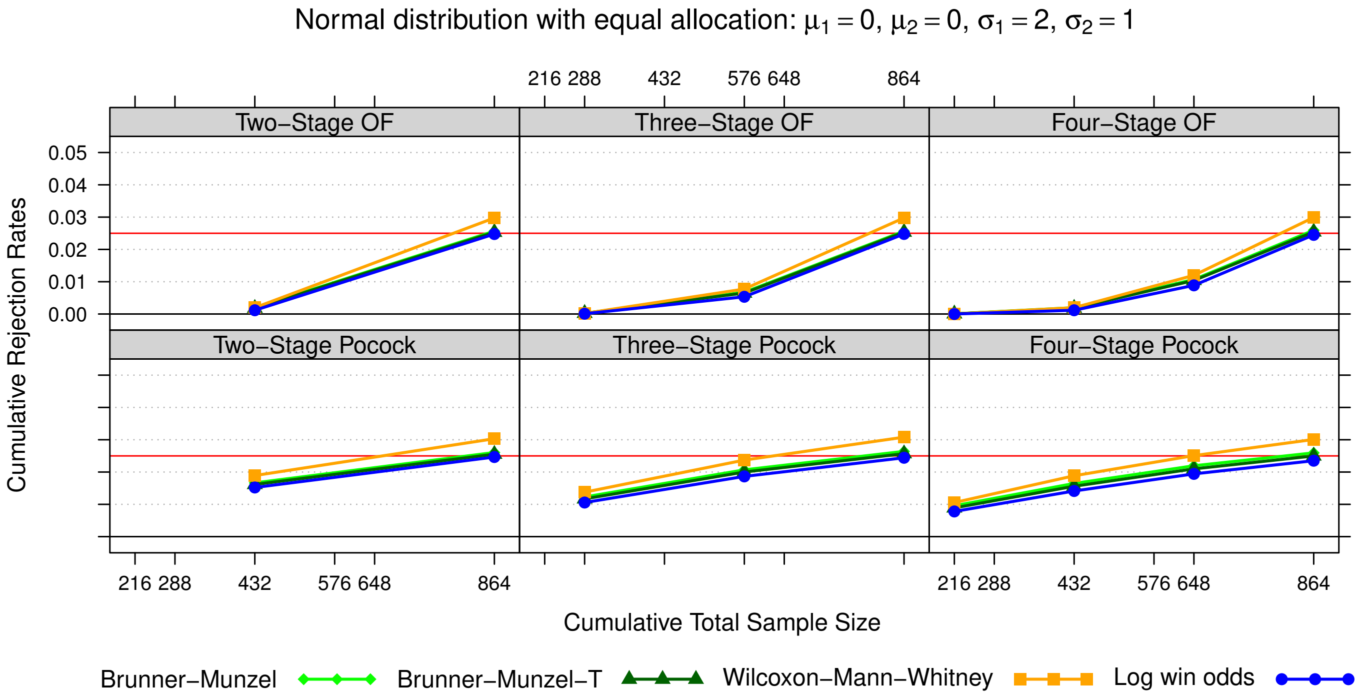

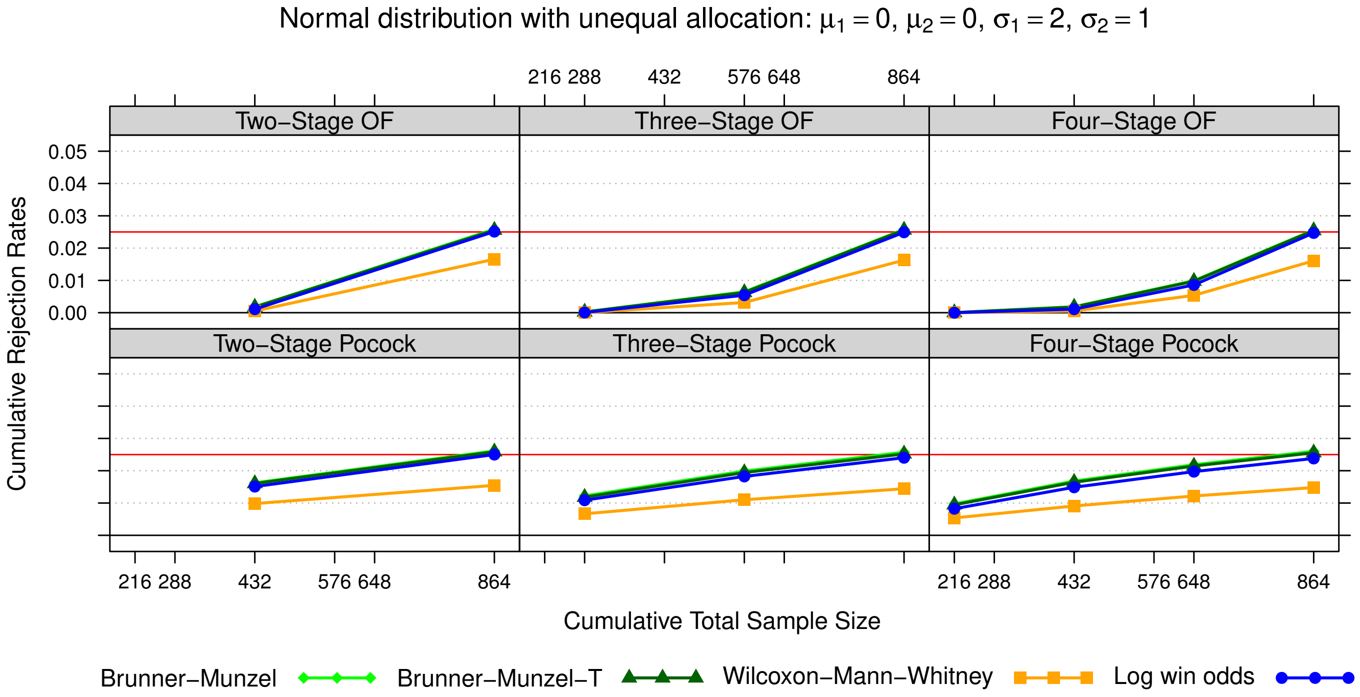

Figure 17: Normal distribution - Setting 3 with total maximum sample size 864

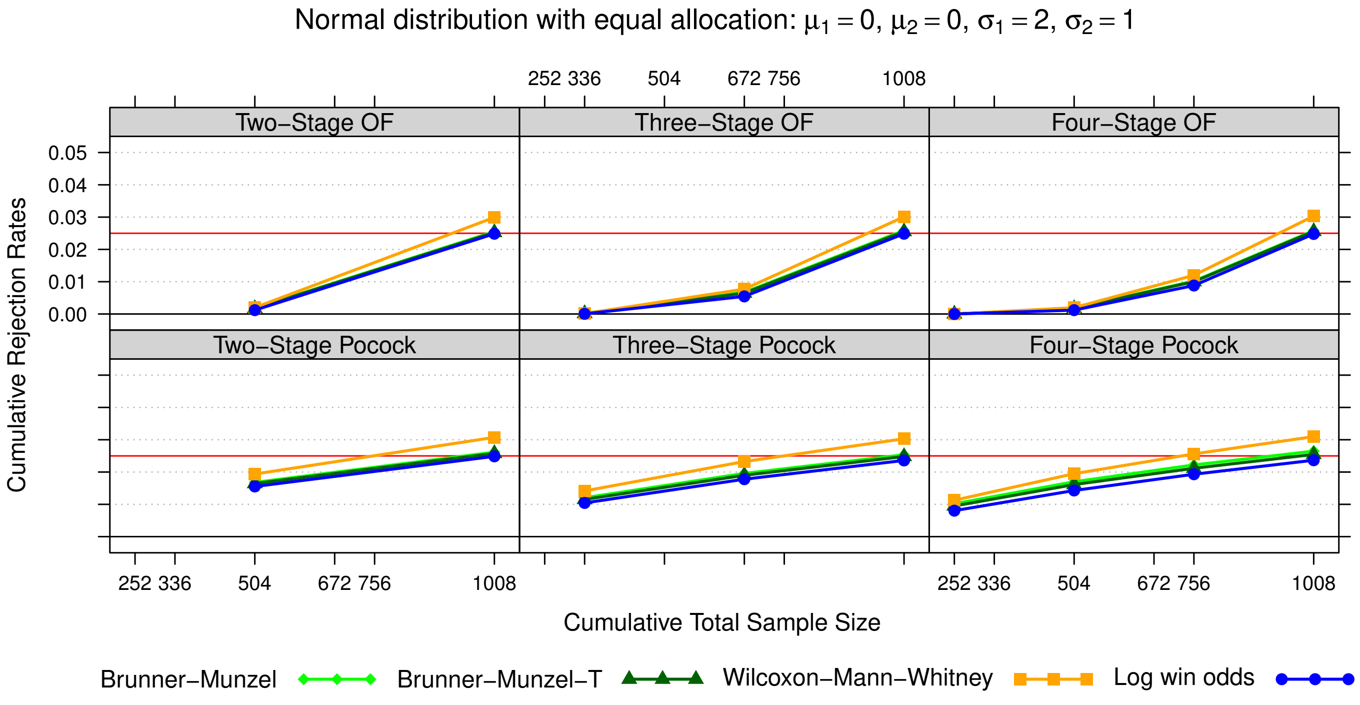

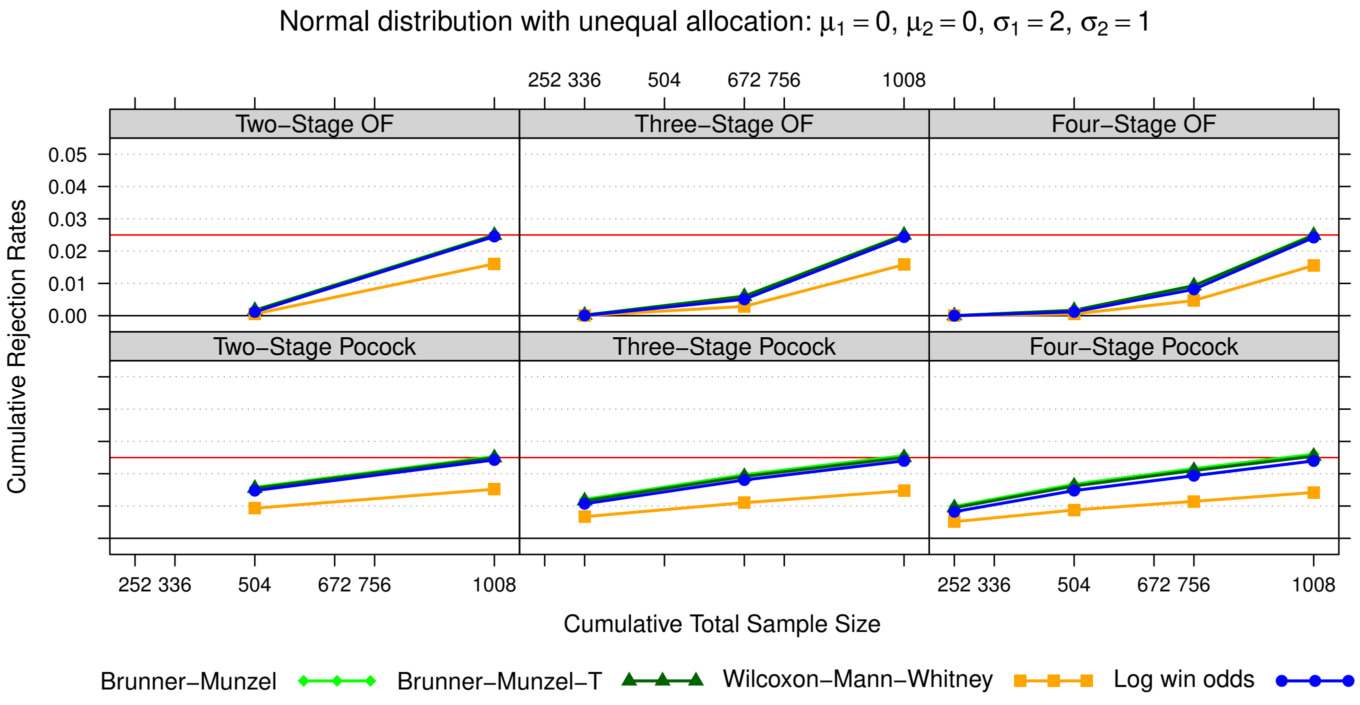

Figure 18: Normal distribution - Setting 3 with total maximum sample size 1008

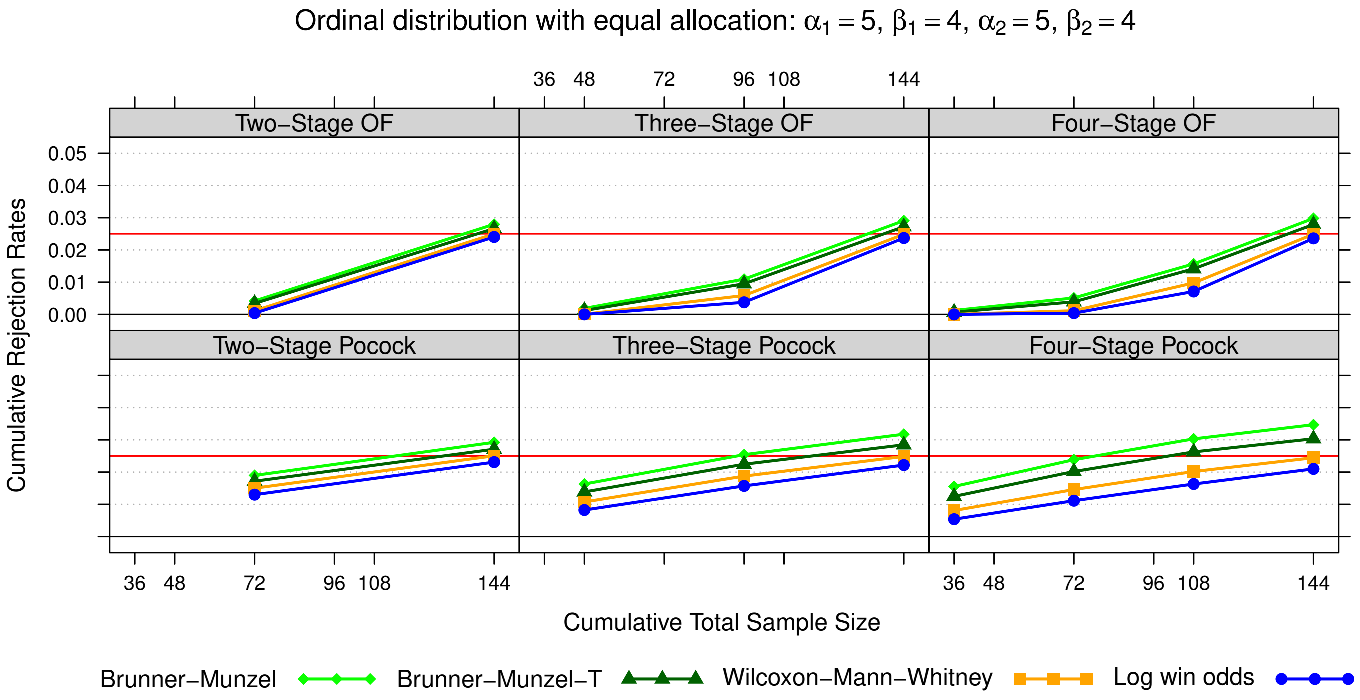

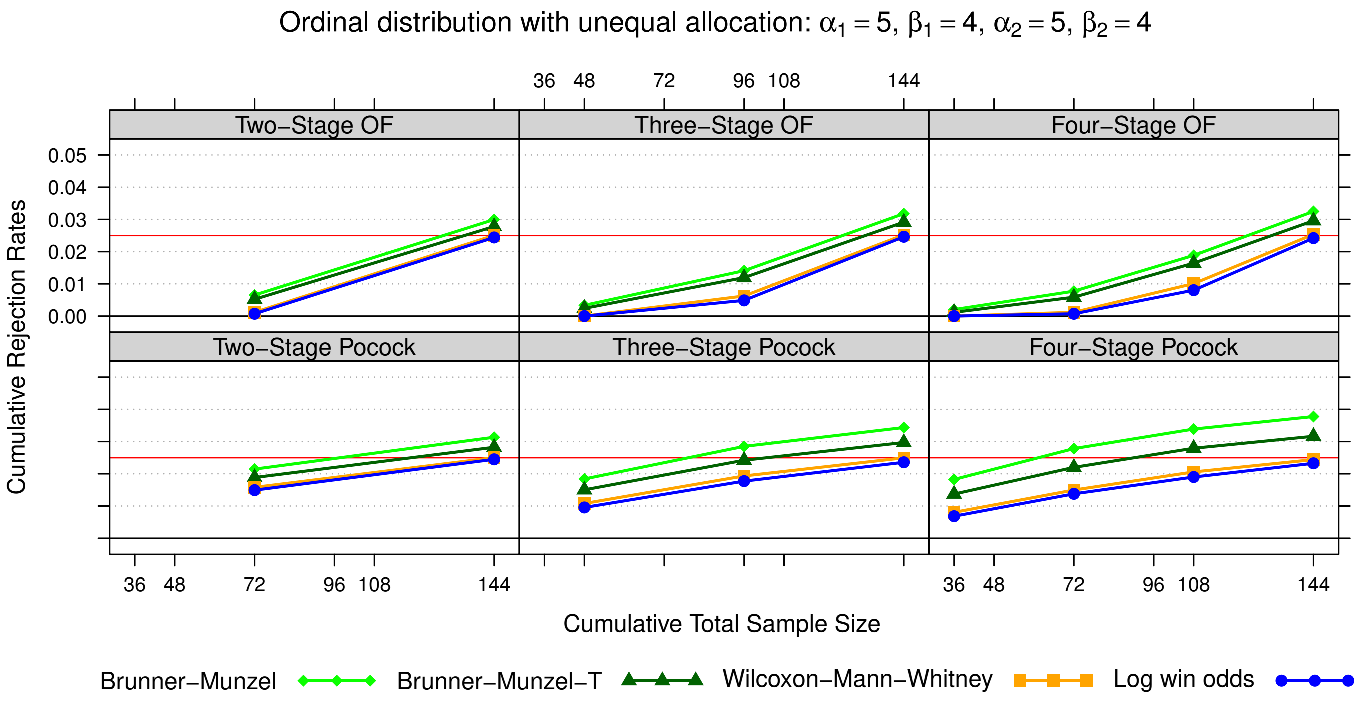

Figure 19: Ordinal distribution - Setting 1 with total maximum sample size 144

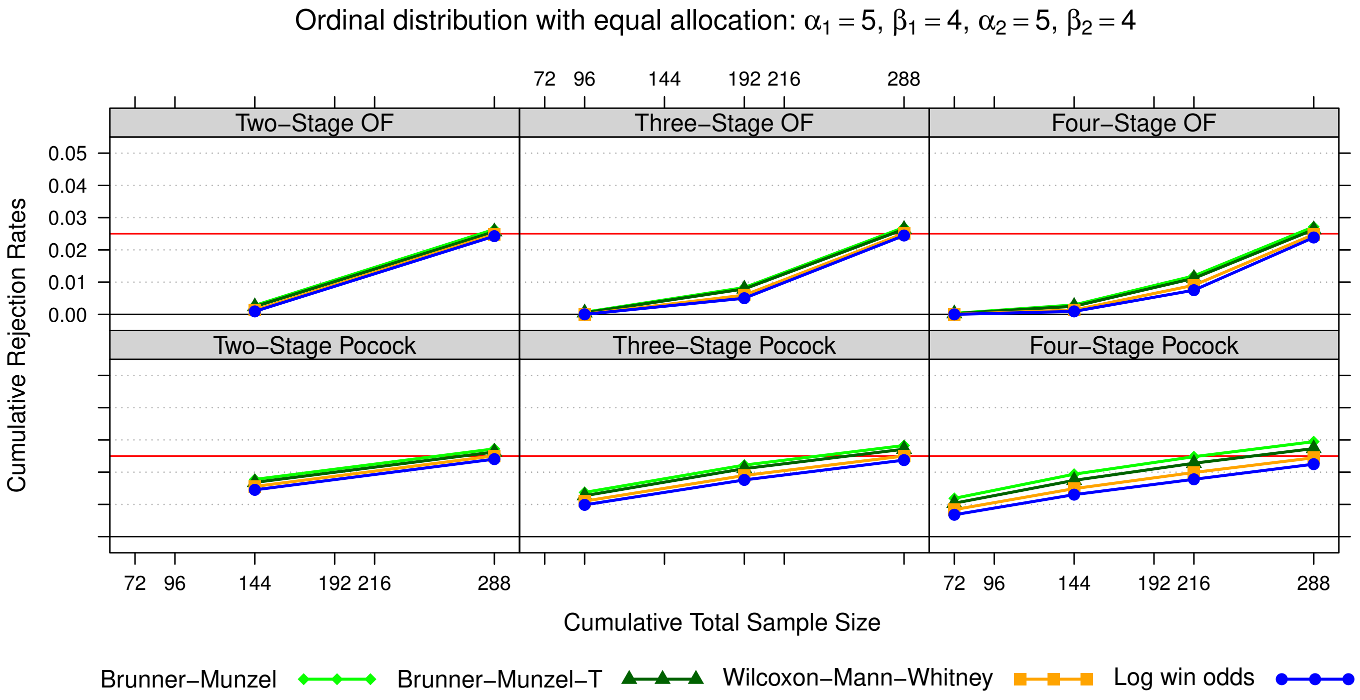

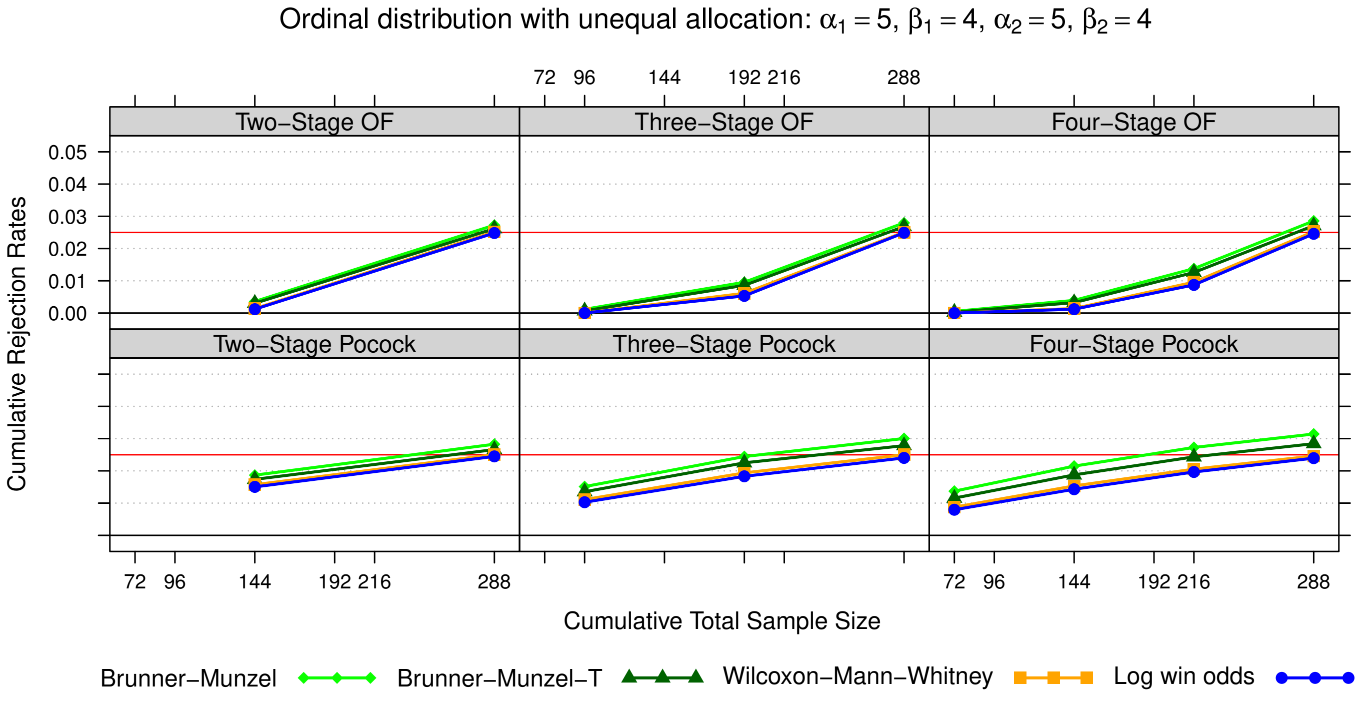

Figure 20: Ordinal distribution - Setting 1 with total maximum sample size 288

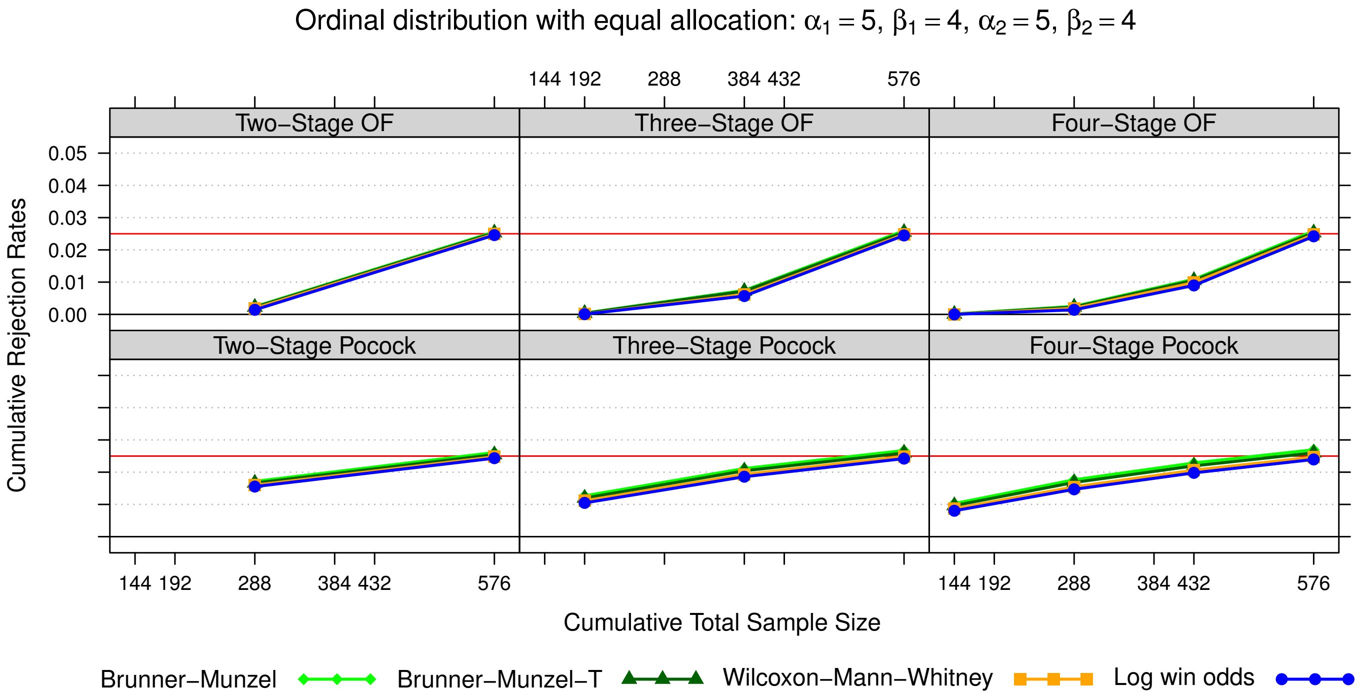

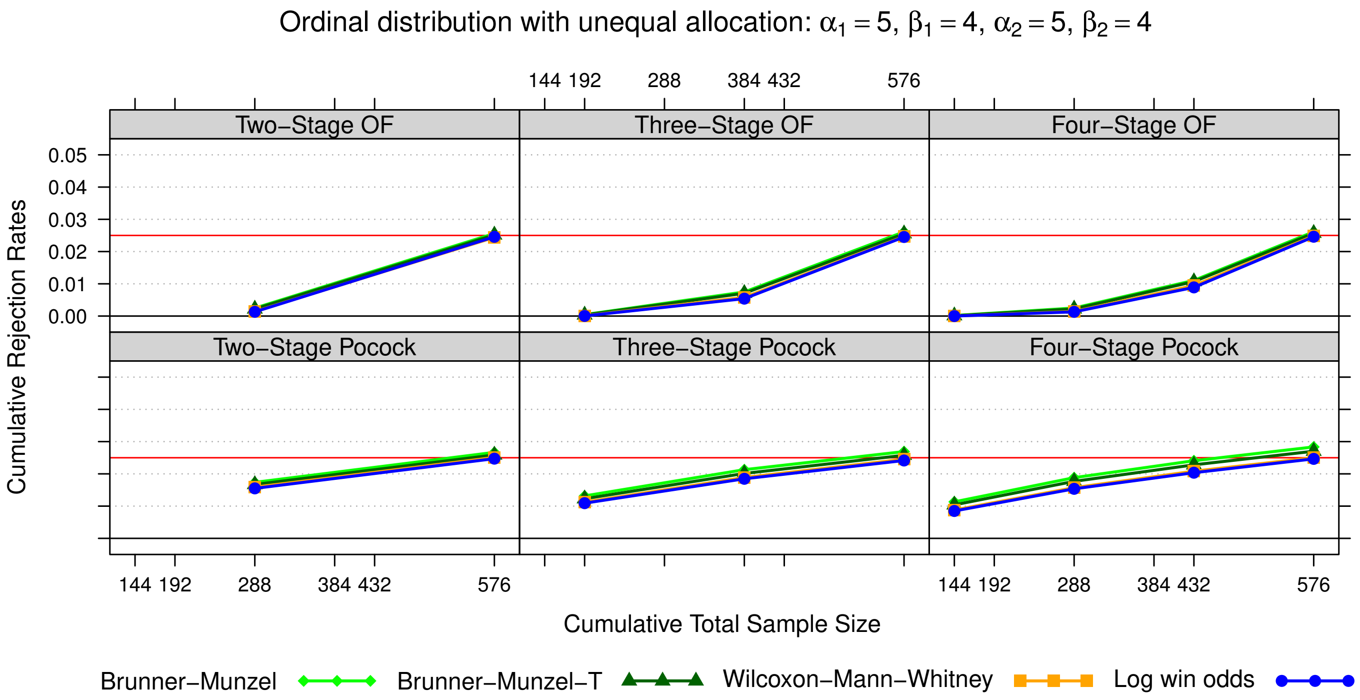

Figure 21: Ordinal distribution - Setting 1 with total maximum sample size 576

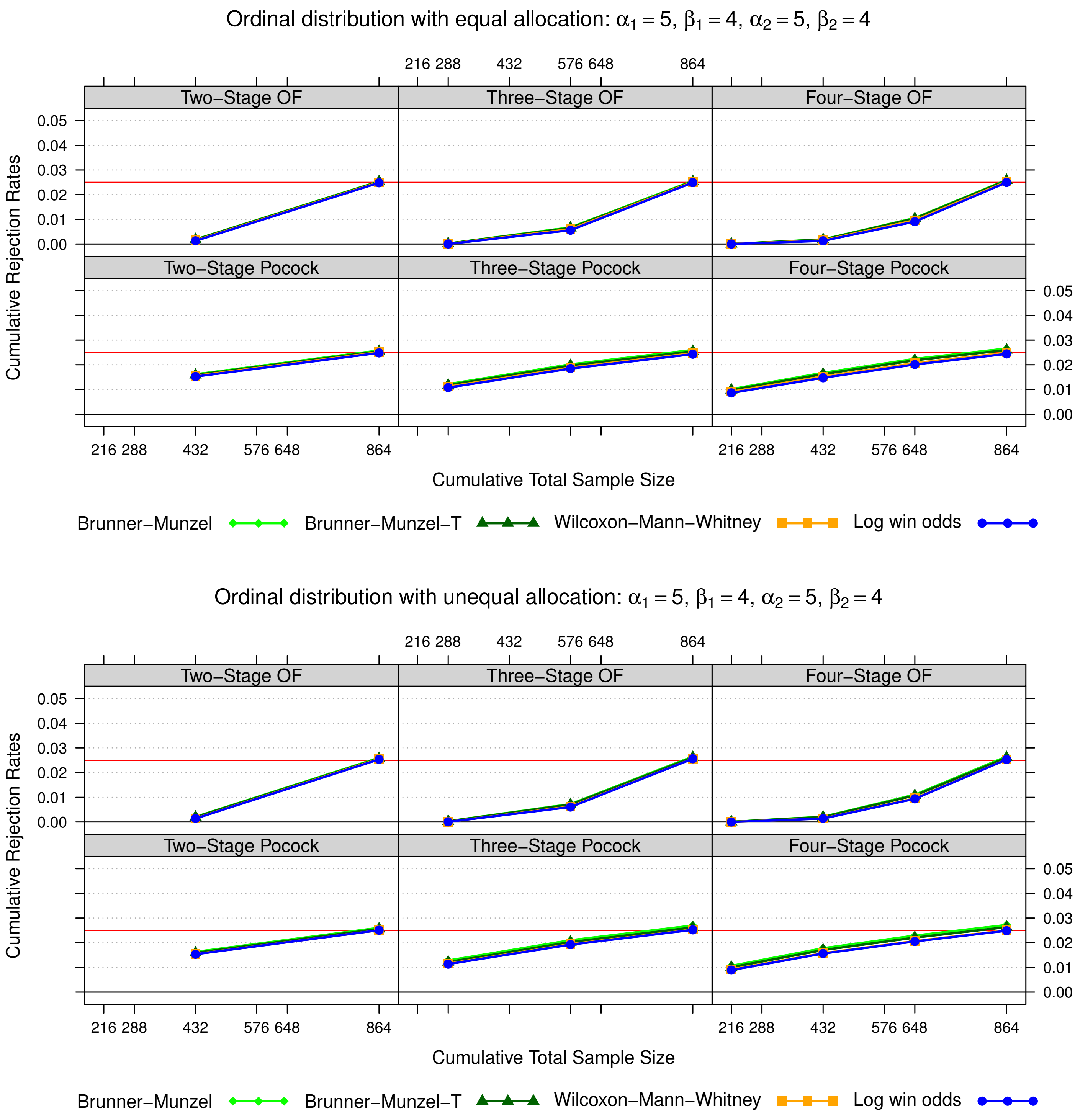

Figure 22: Ordinal distribution - Setting 1 with total maximum sample size 864

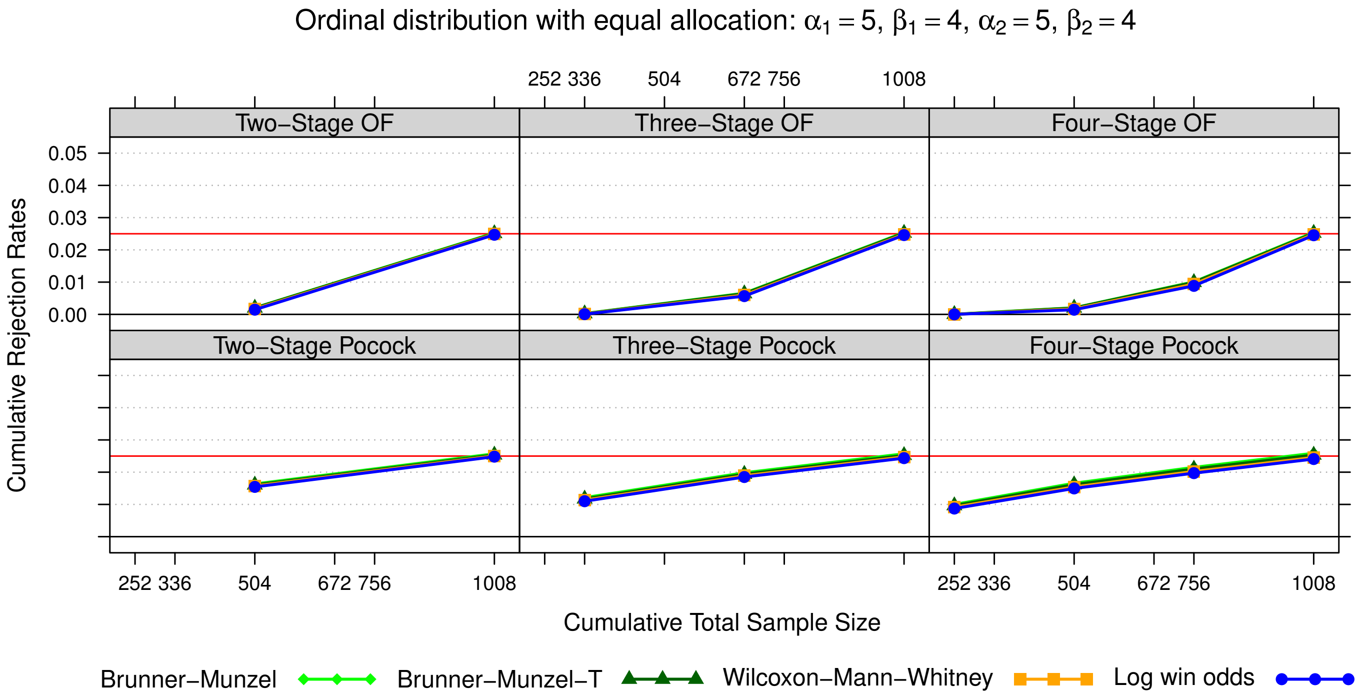

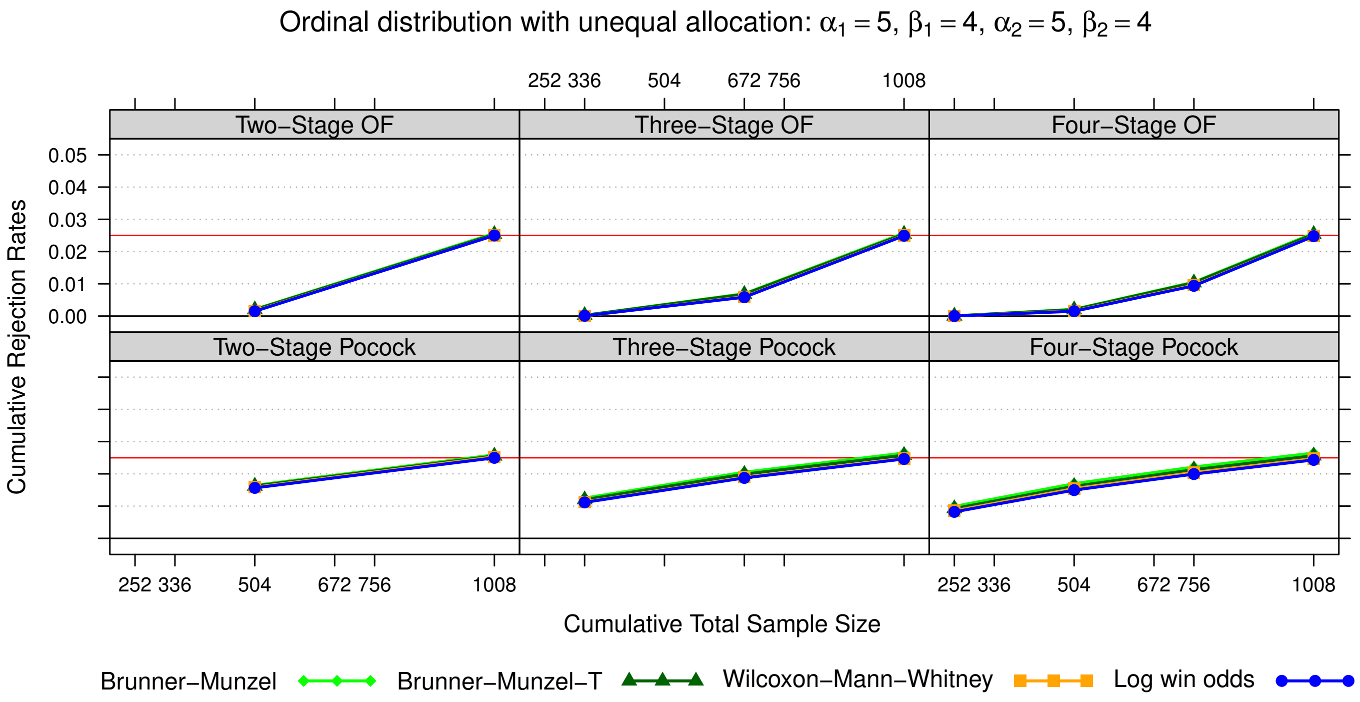

Figure 23: Ordinal distribution - Setting 1 with total maximum sample size 1008

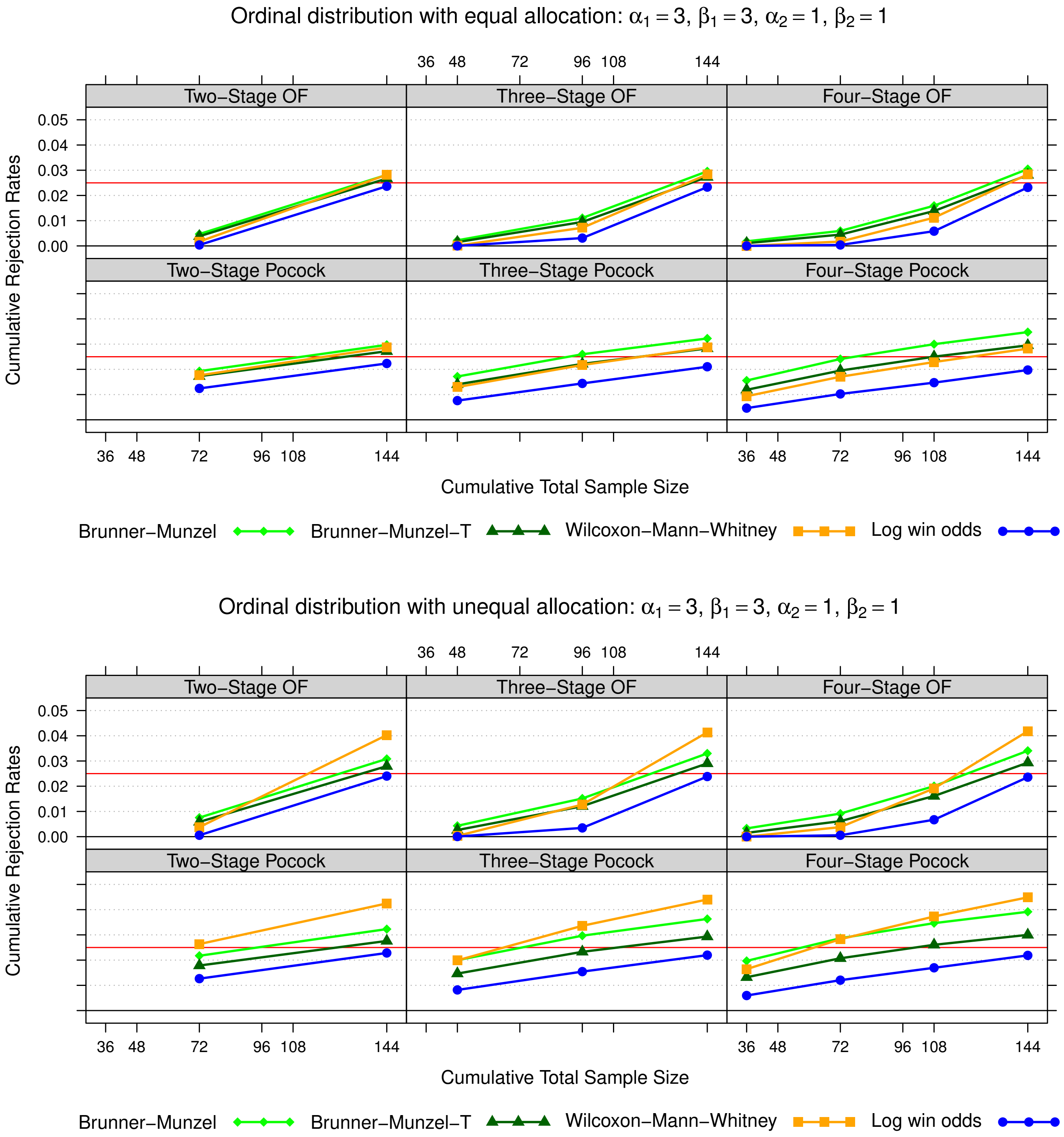

Figure 24: Ordinal distribution - Setting 2 with total maximum sample size 144

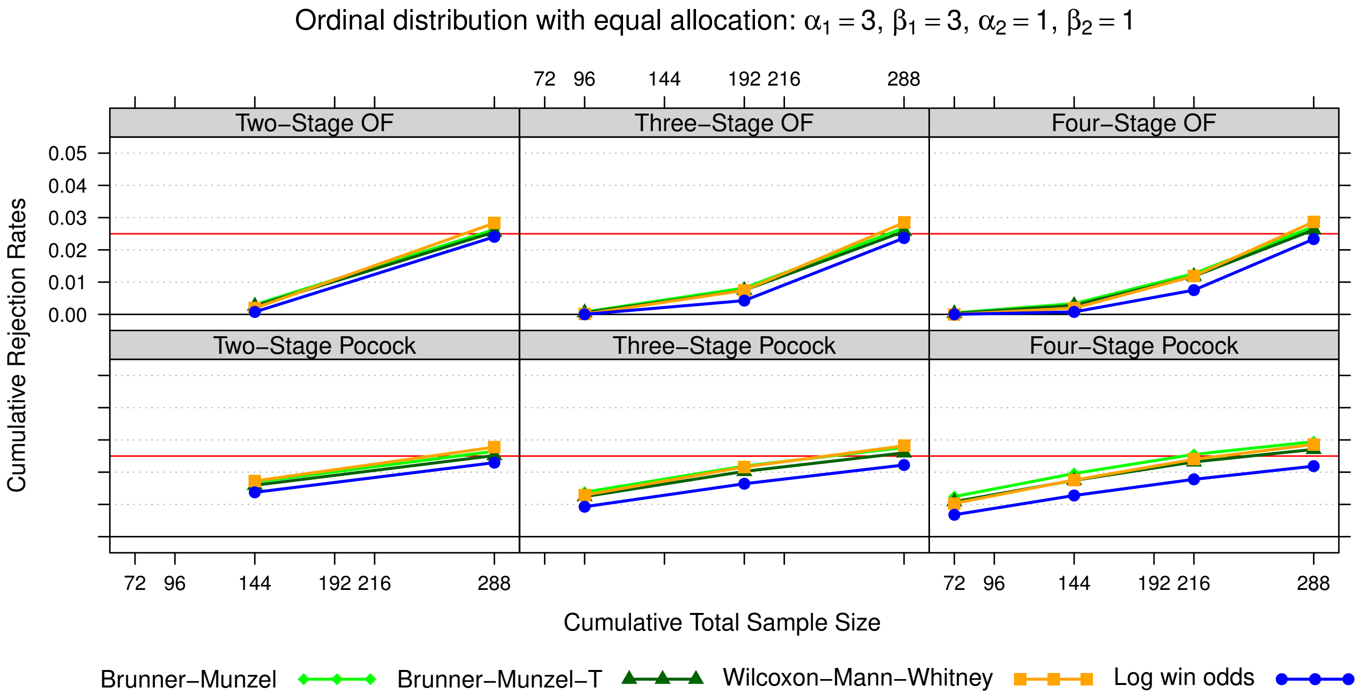

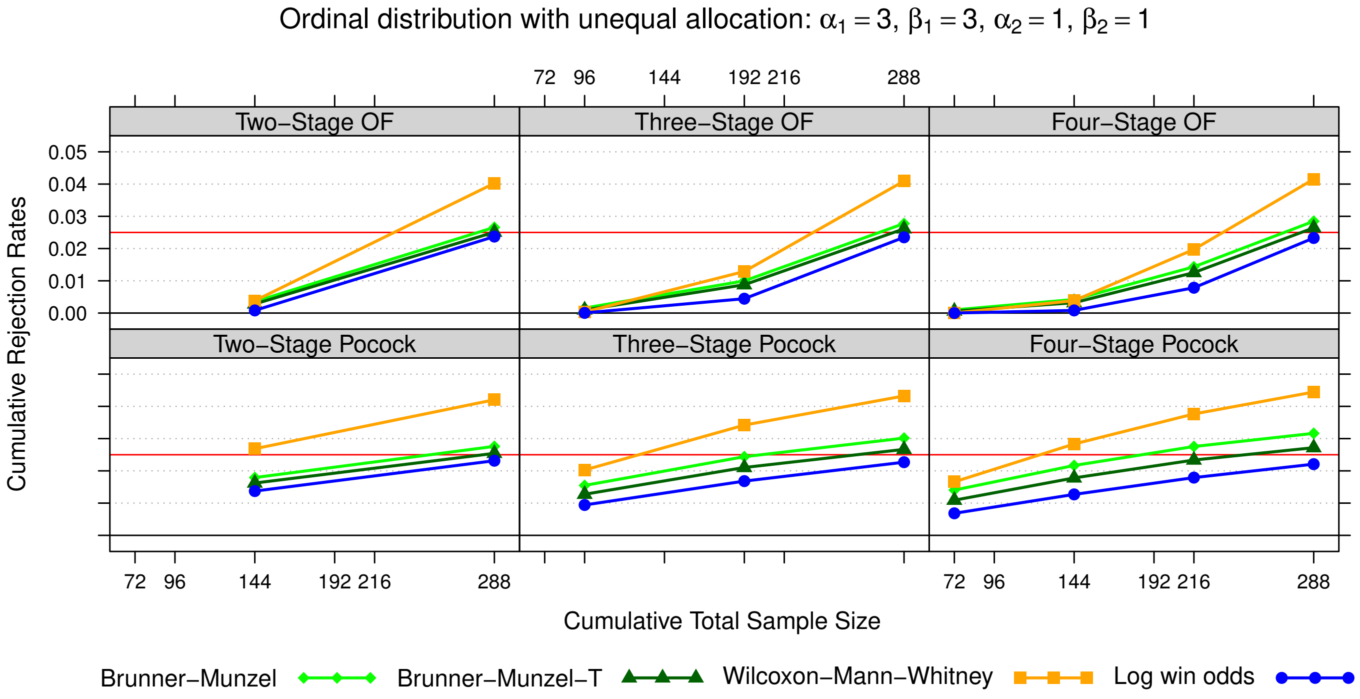

Figure 25: Ordinal distribution - Setting 2 with total maximum sample size 288

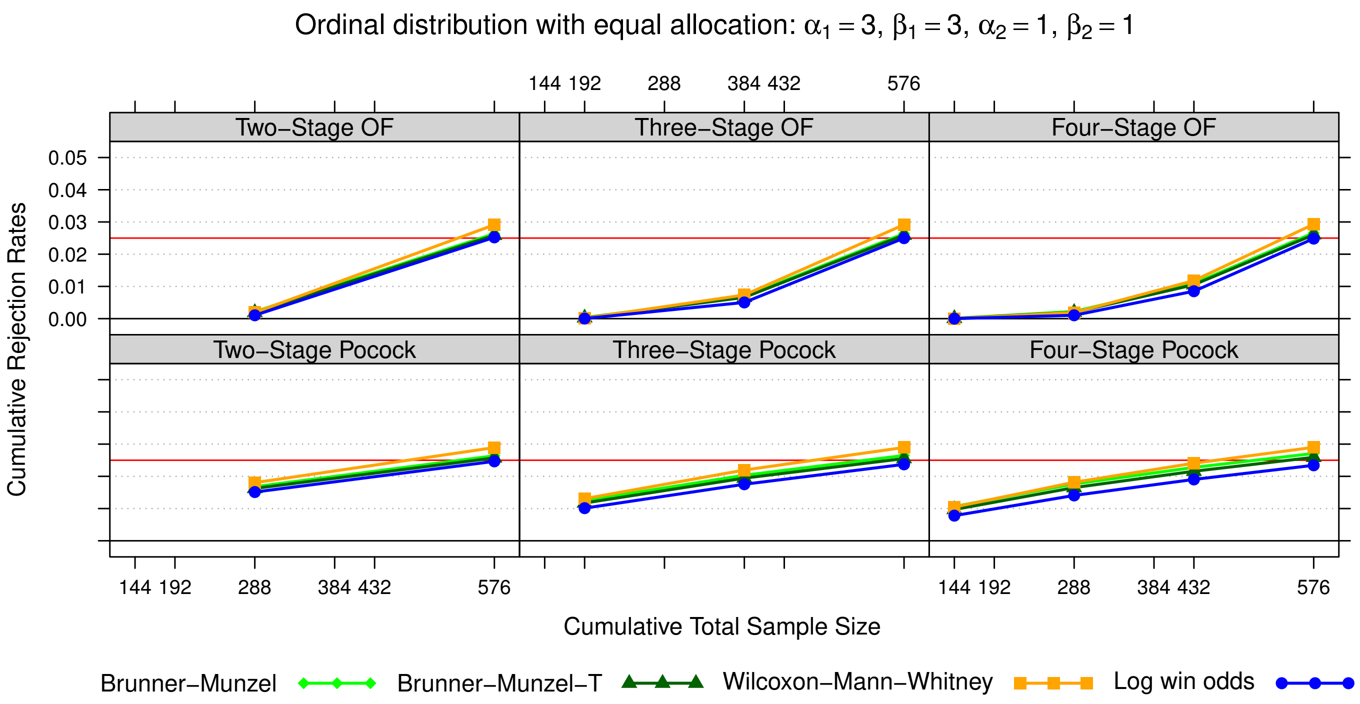

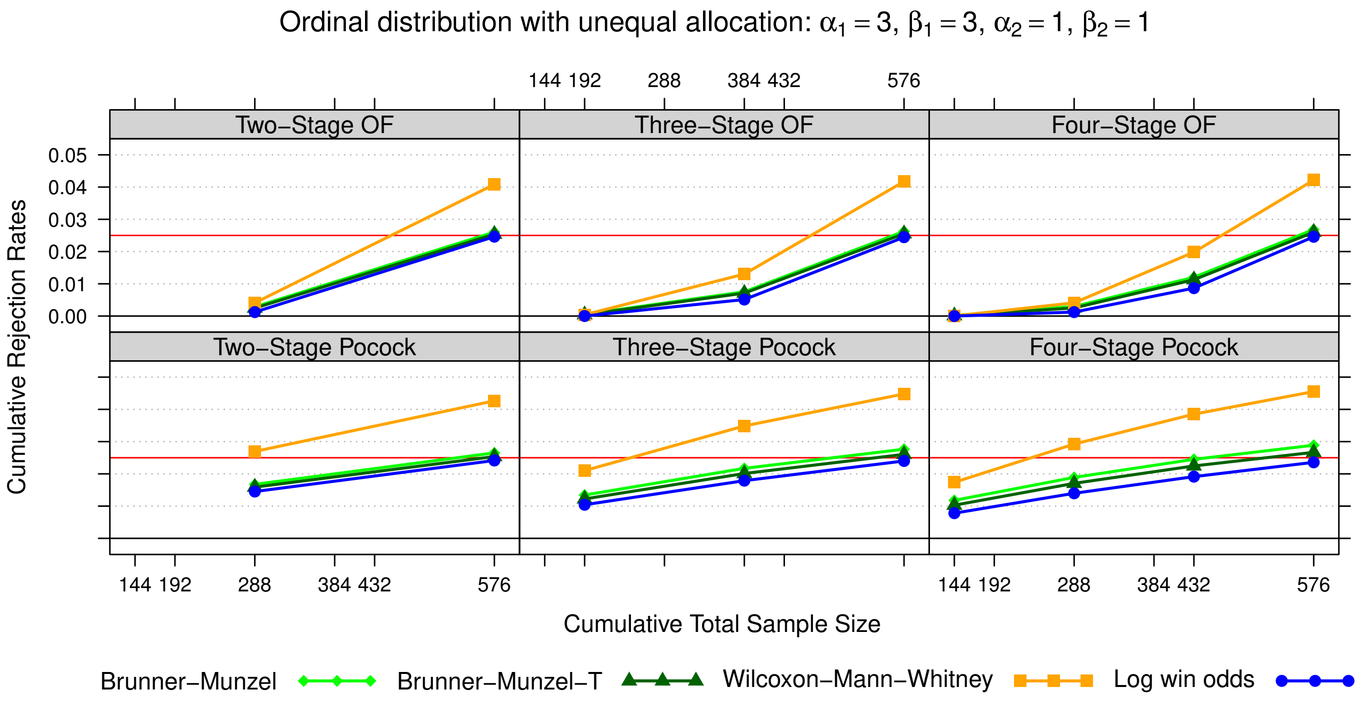

Figure 26: Ordinal distribution - Setting 2 with total maximum sample size 576

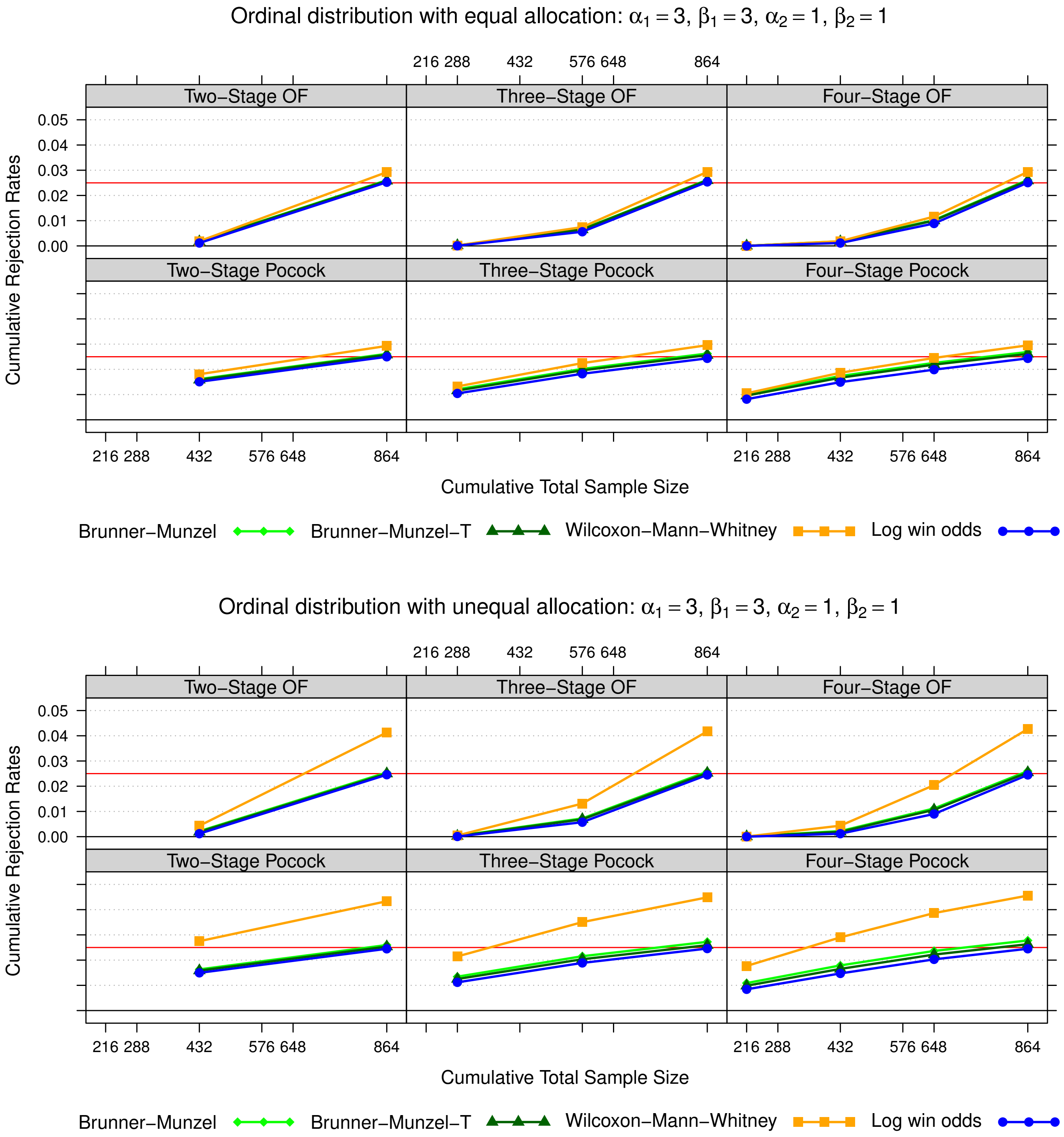

Figure 27: Ordinal distribution - Setting 2 with total maximum sample size 864

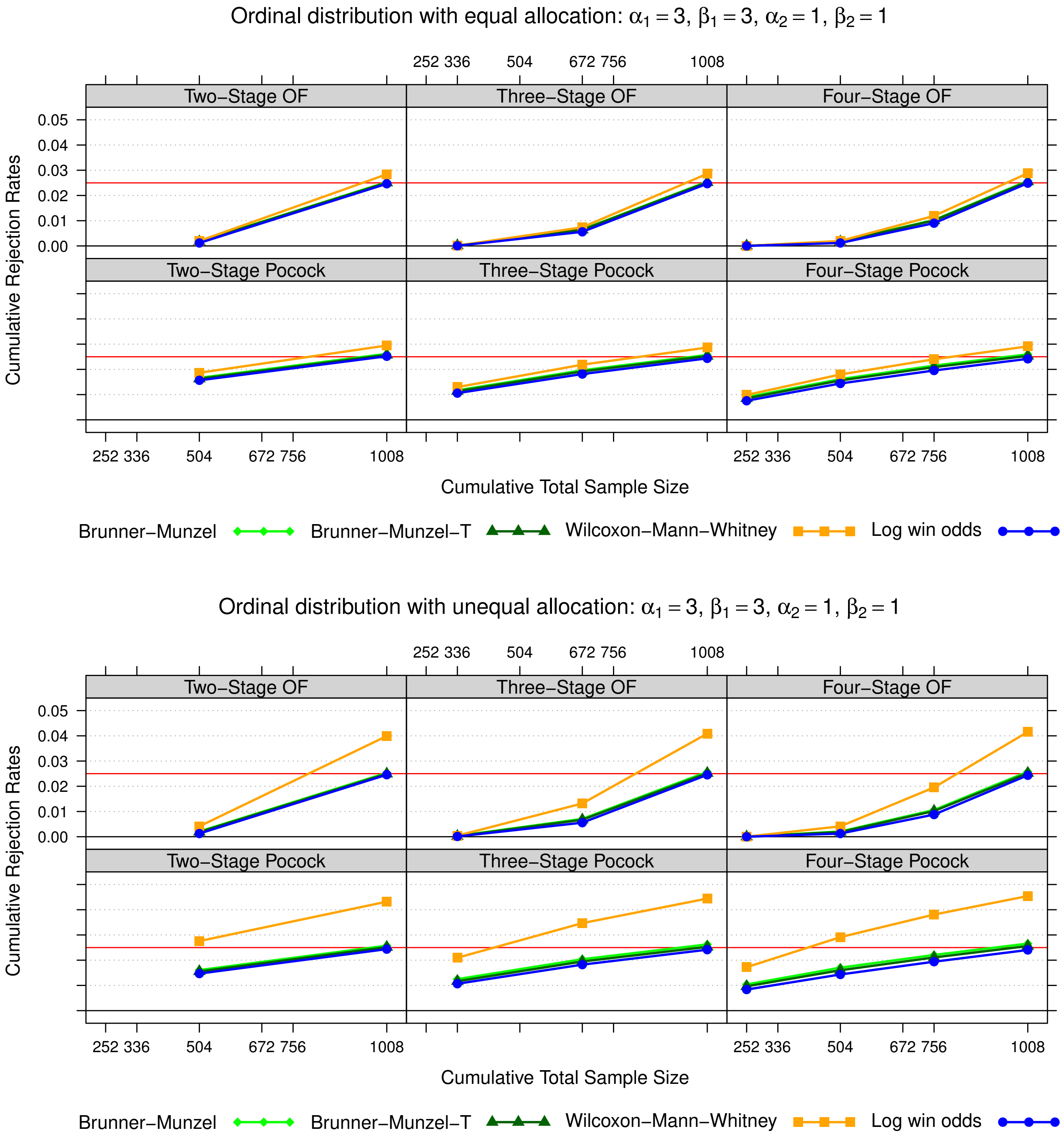

Figure 28: Ordinal distribution - Setting 2 with total maximum sample size 1008

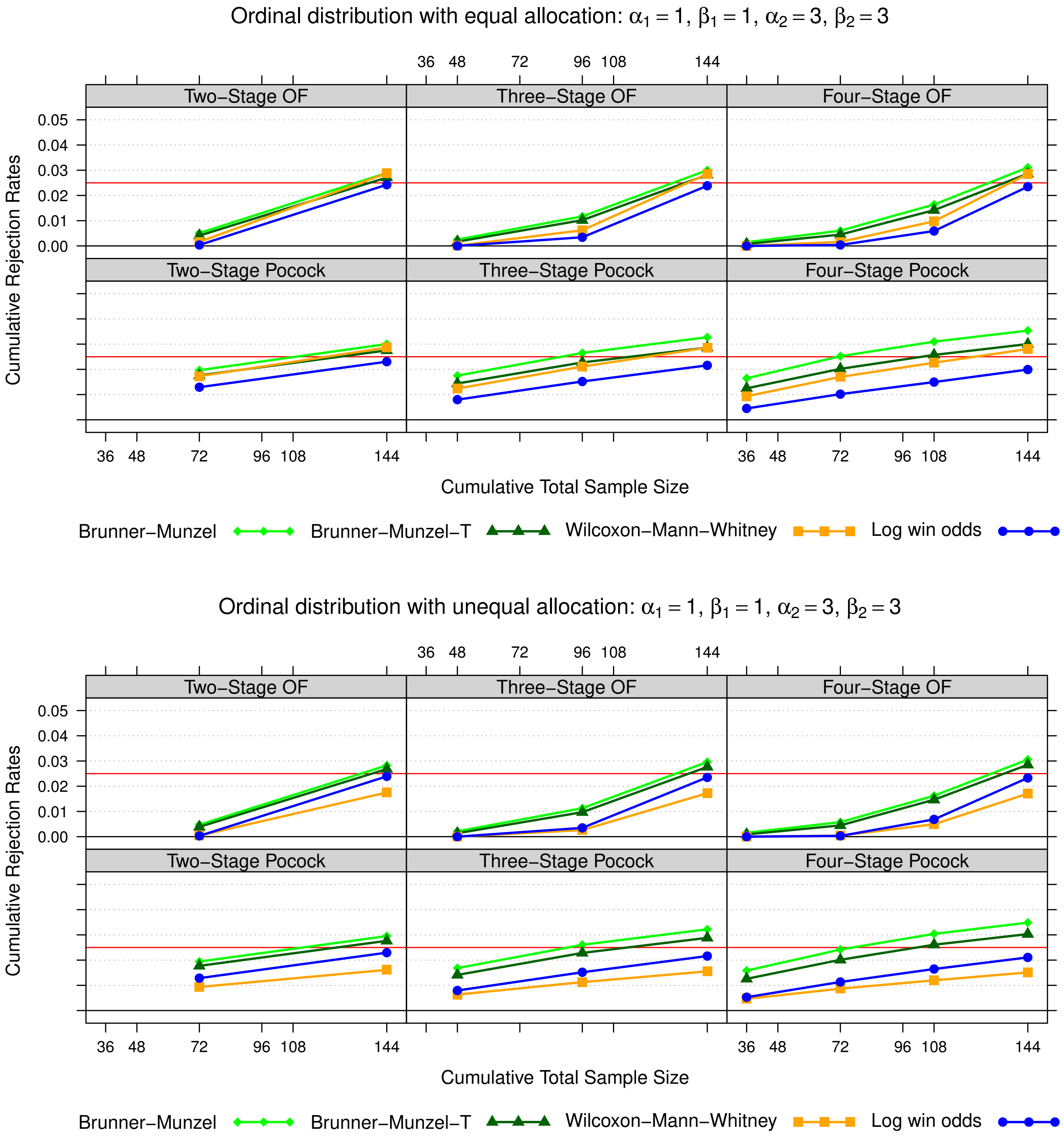

Figure 29: Ordinal distribution - Setting 3 with total maximum sample size 144

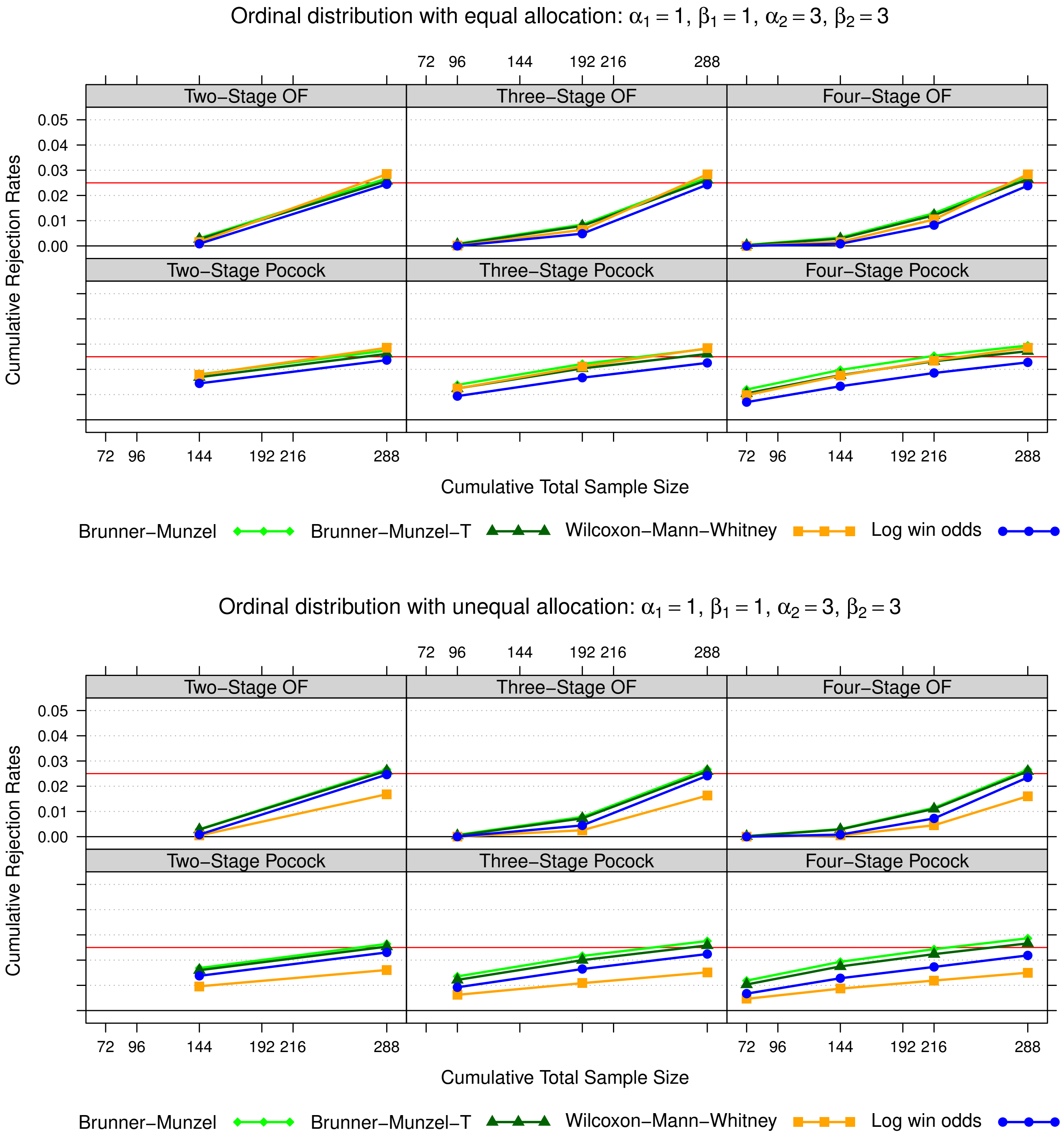

Figure 30: Ordinal distribution - Setting 3 with total maximum sample size 288

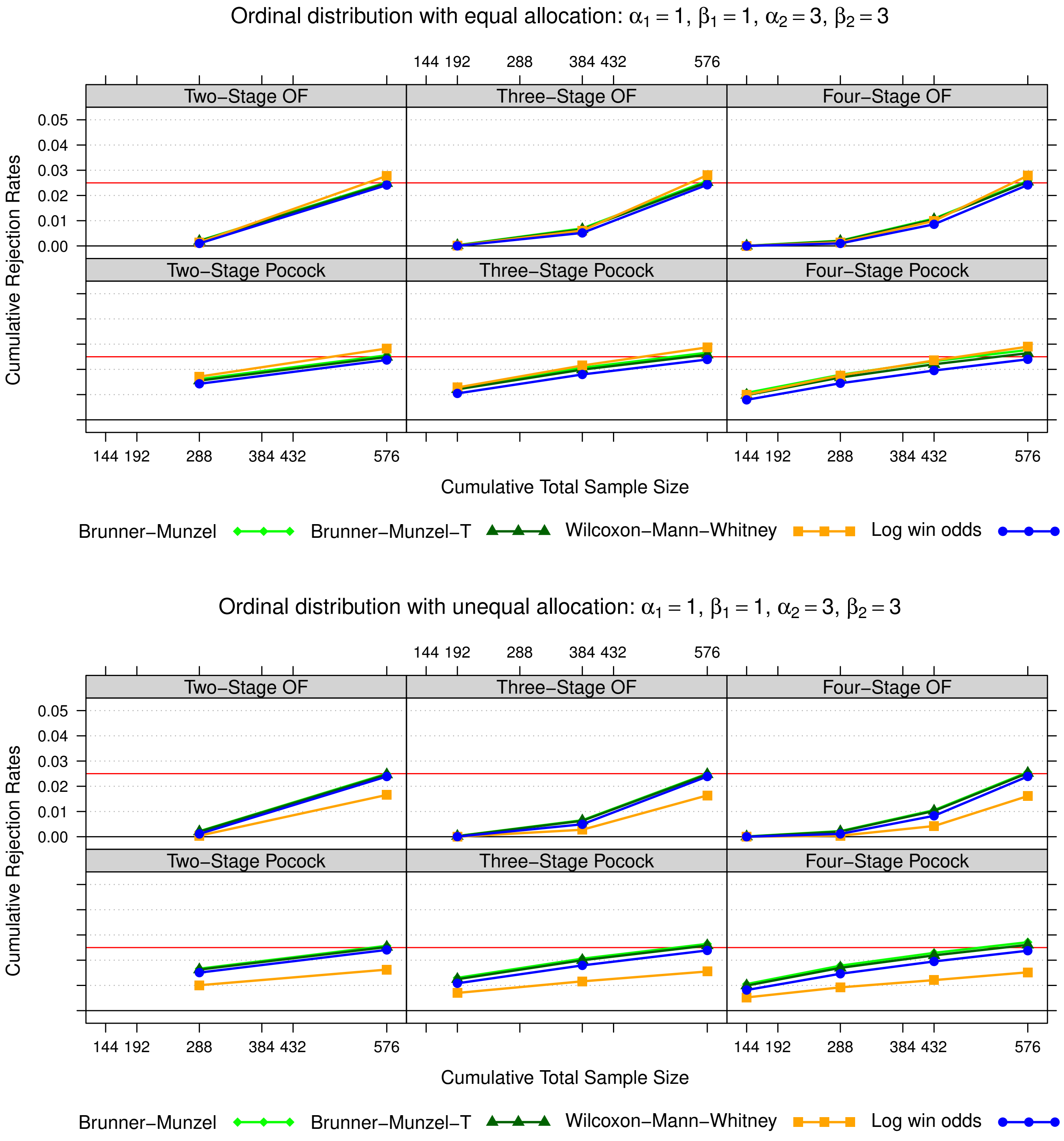

Figure 31: Ordinal distribution - Setting 3 with total maximum sample size 576

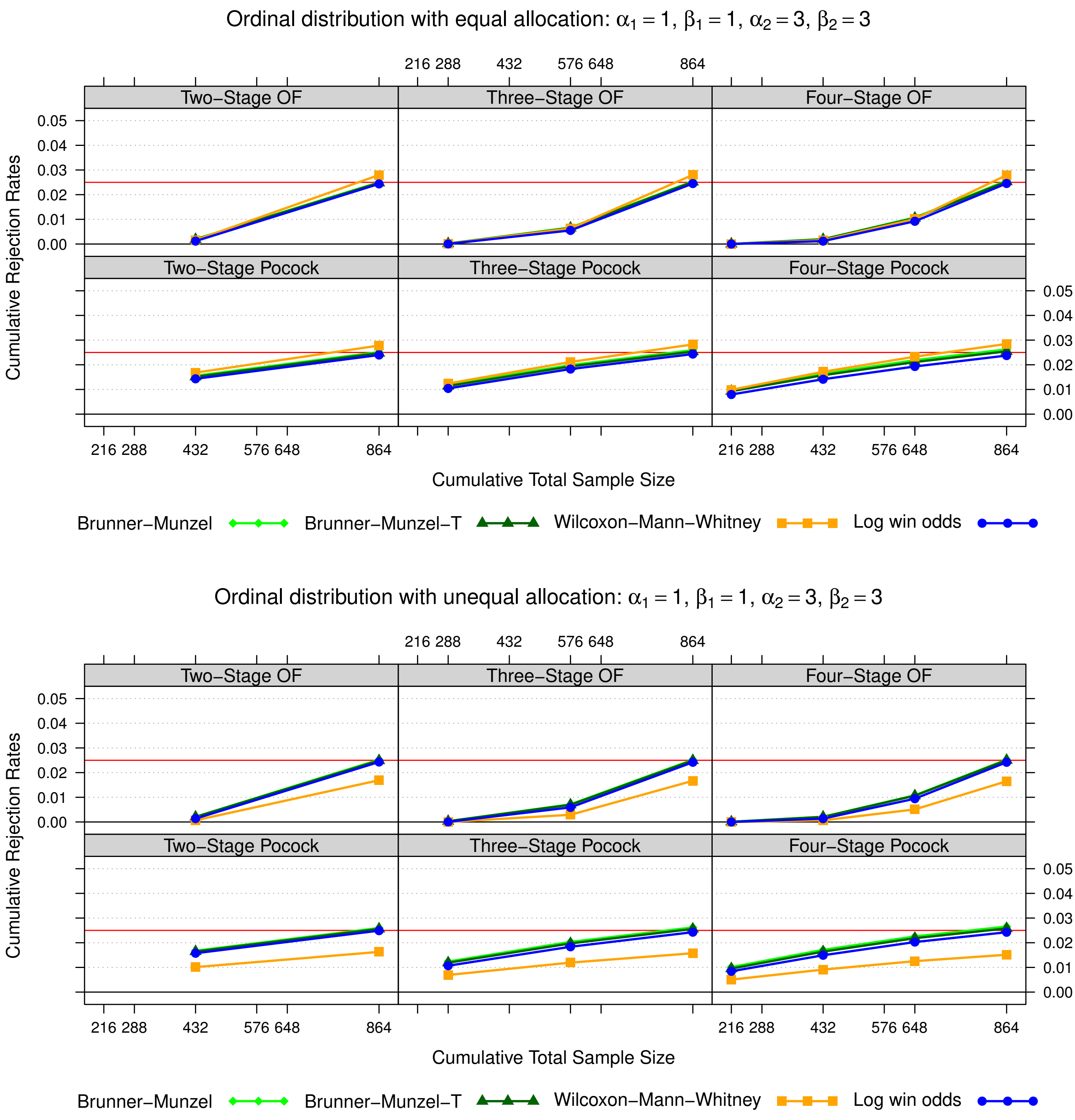

Figure 32: Ordinal distribution - Setting 3 with total maximum sample size 864

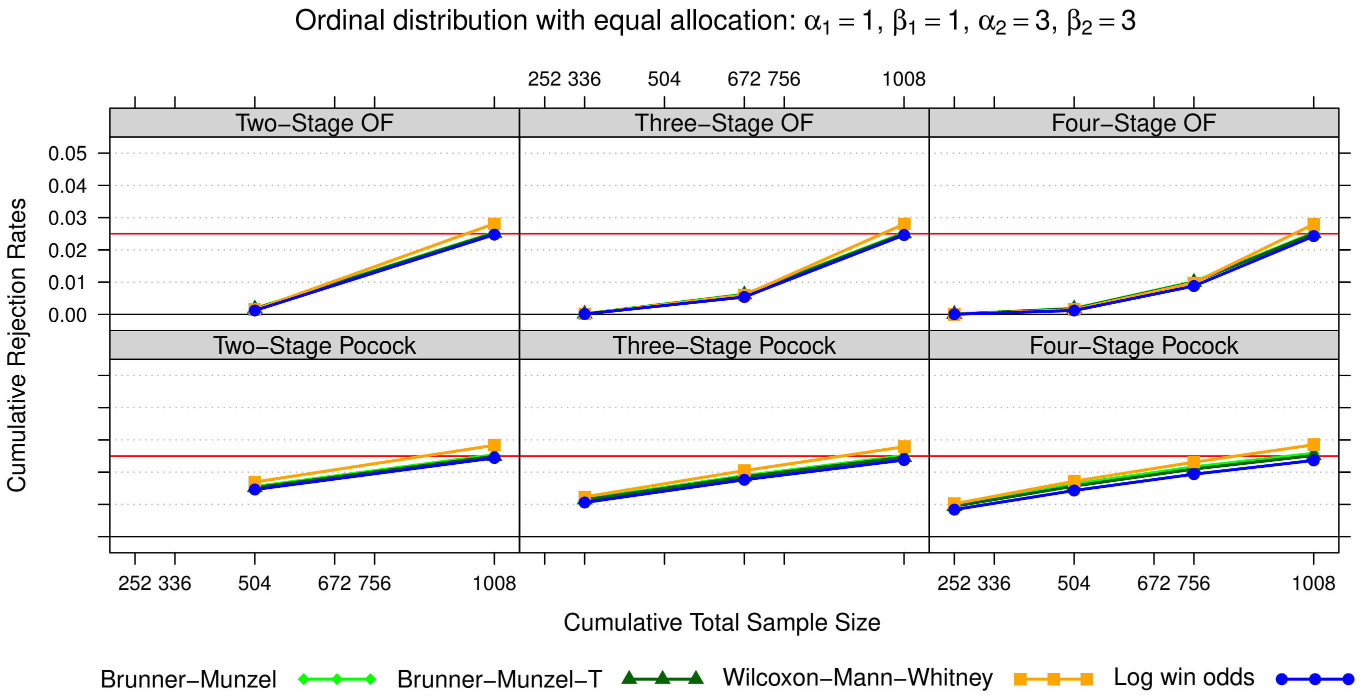

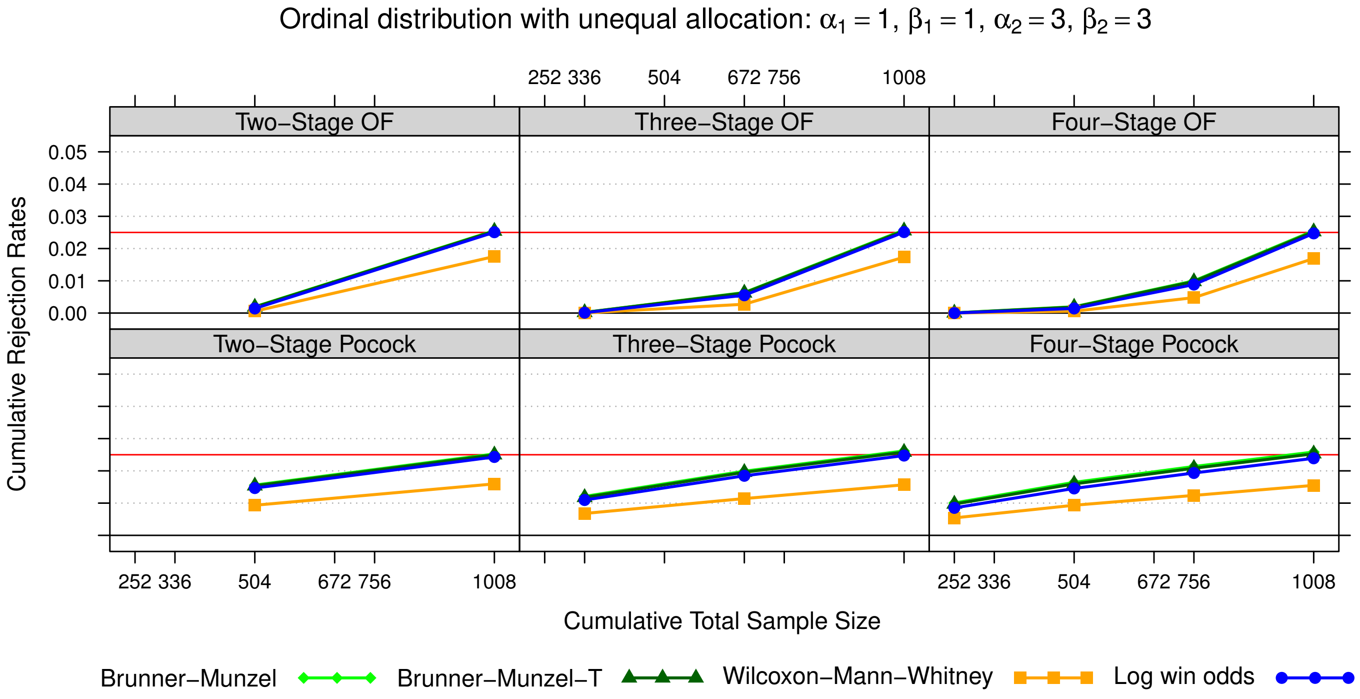

Figure 33: Ordinal distribution - Setting 3 with total maximum sample size 1008

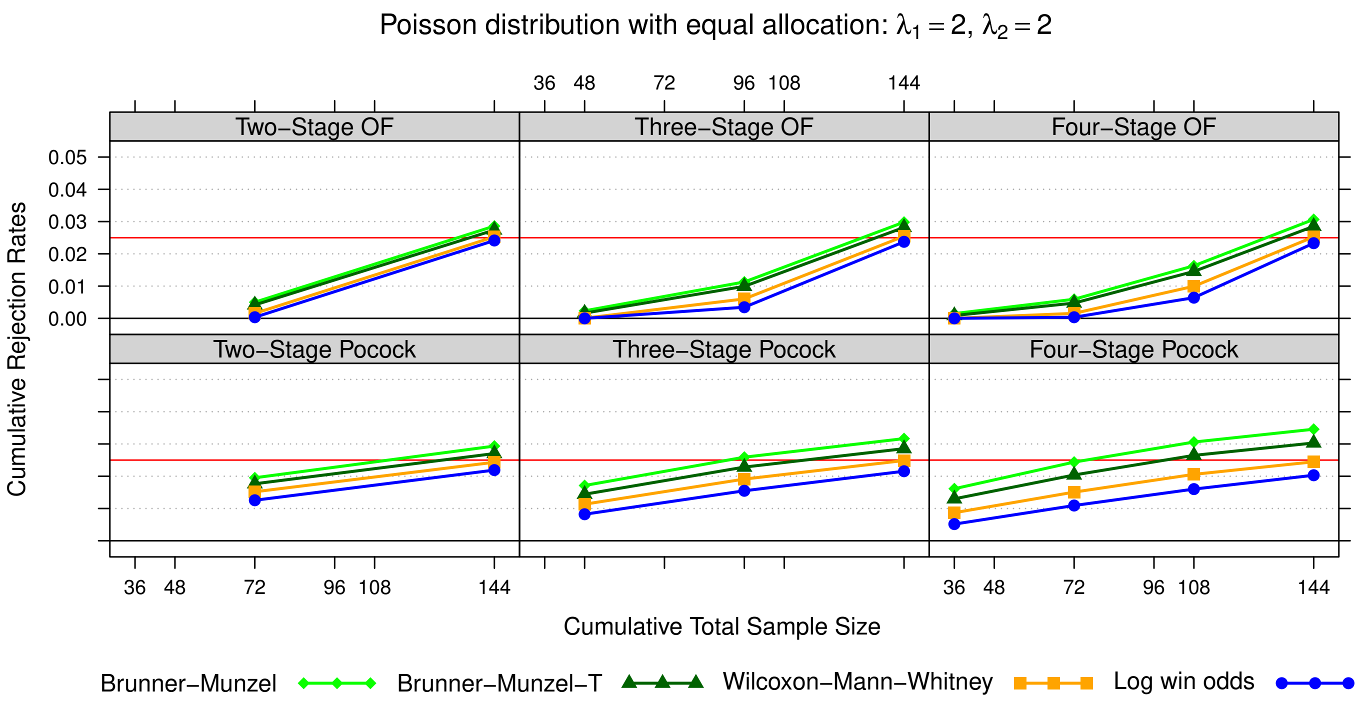

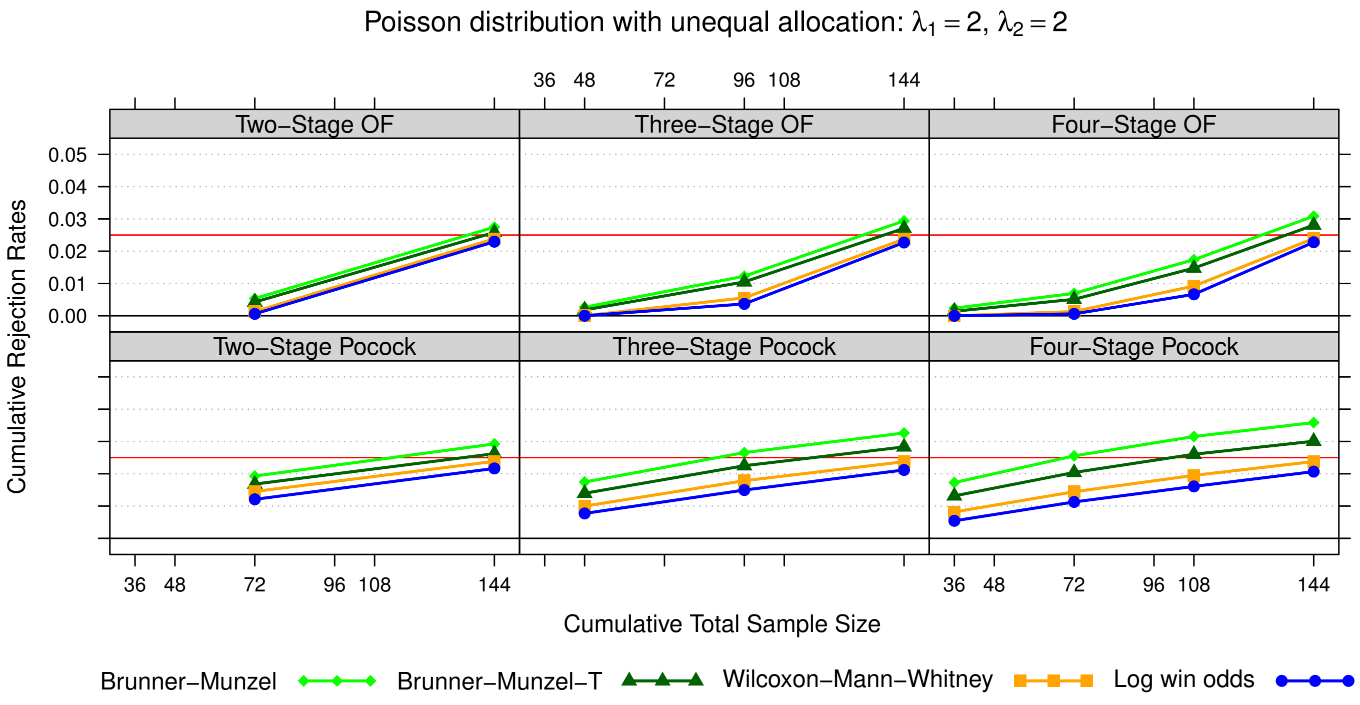

Figure 34: Poisson distribution with total maximum sample size 144

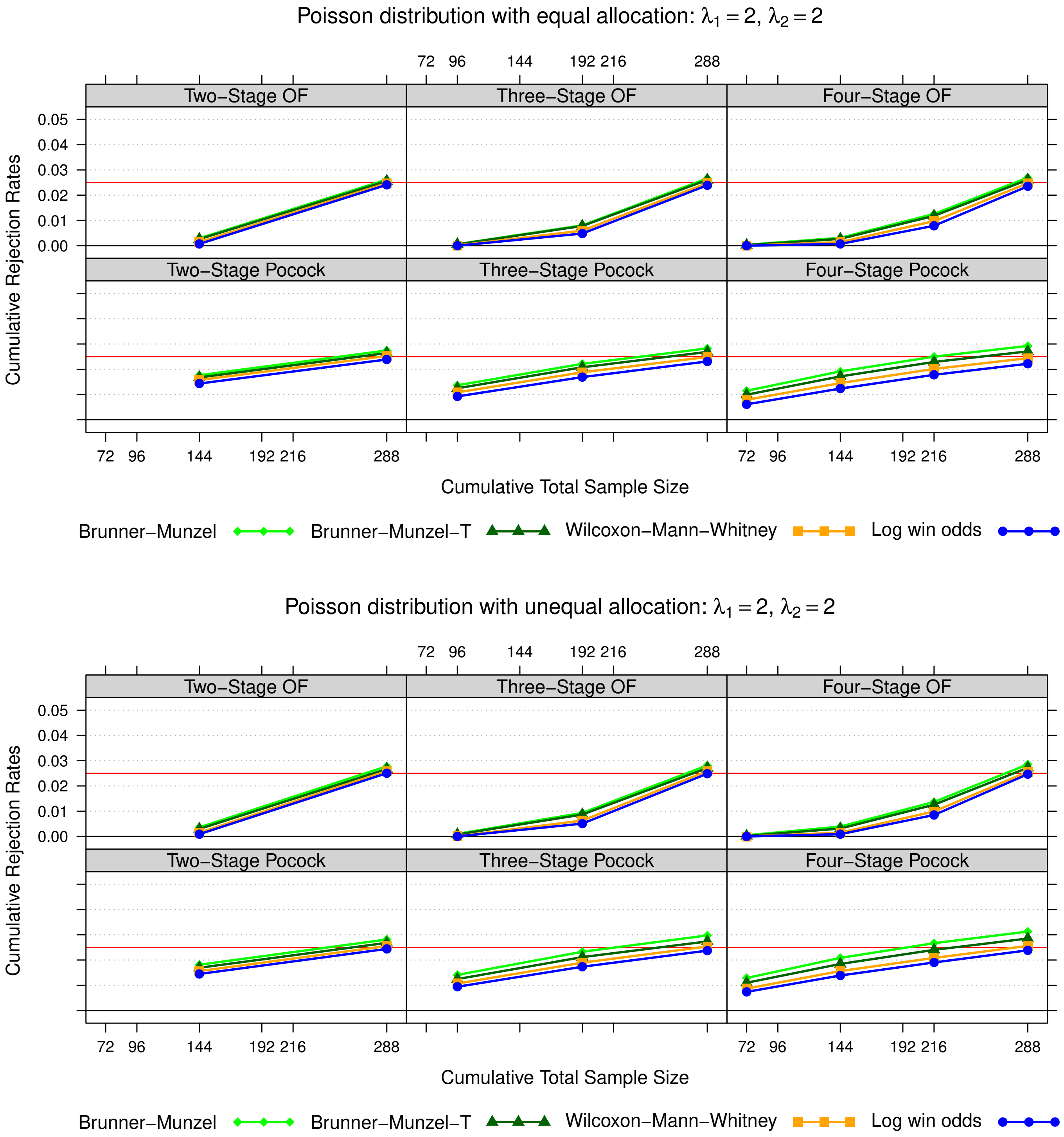

Figure 35: Poisson distribution with total maximum sample size 288

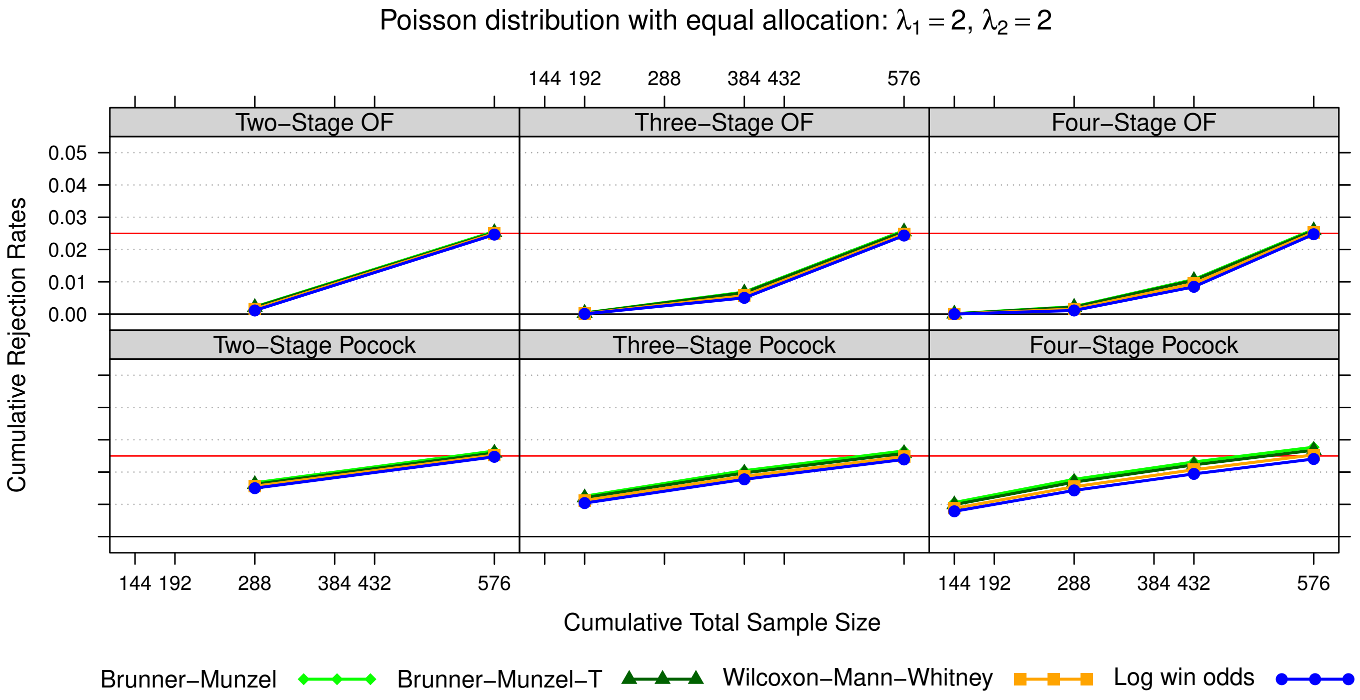

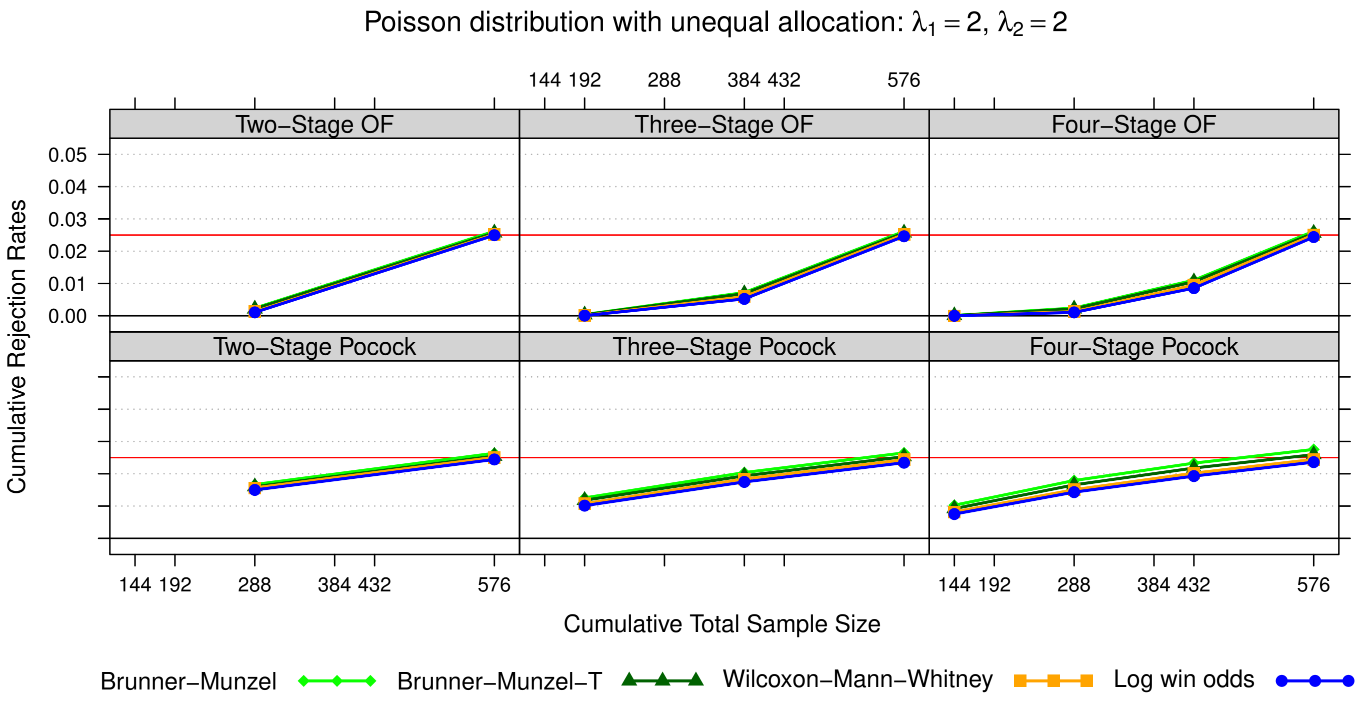

Figure 36: Poisson distribution with total maximum sample size 576

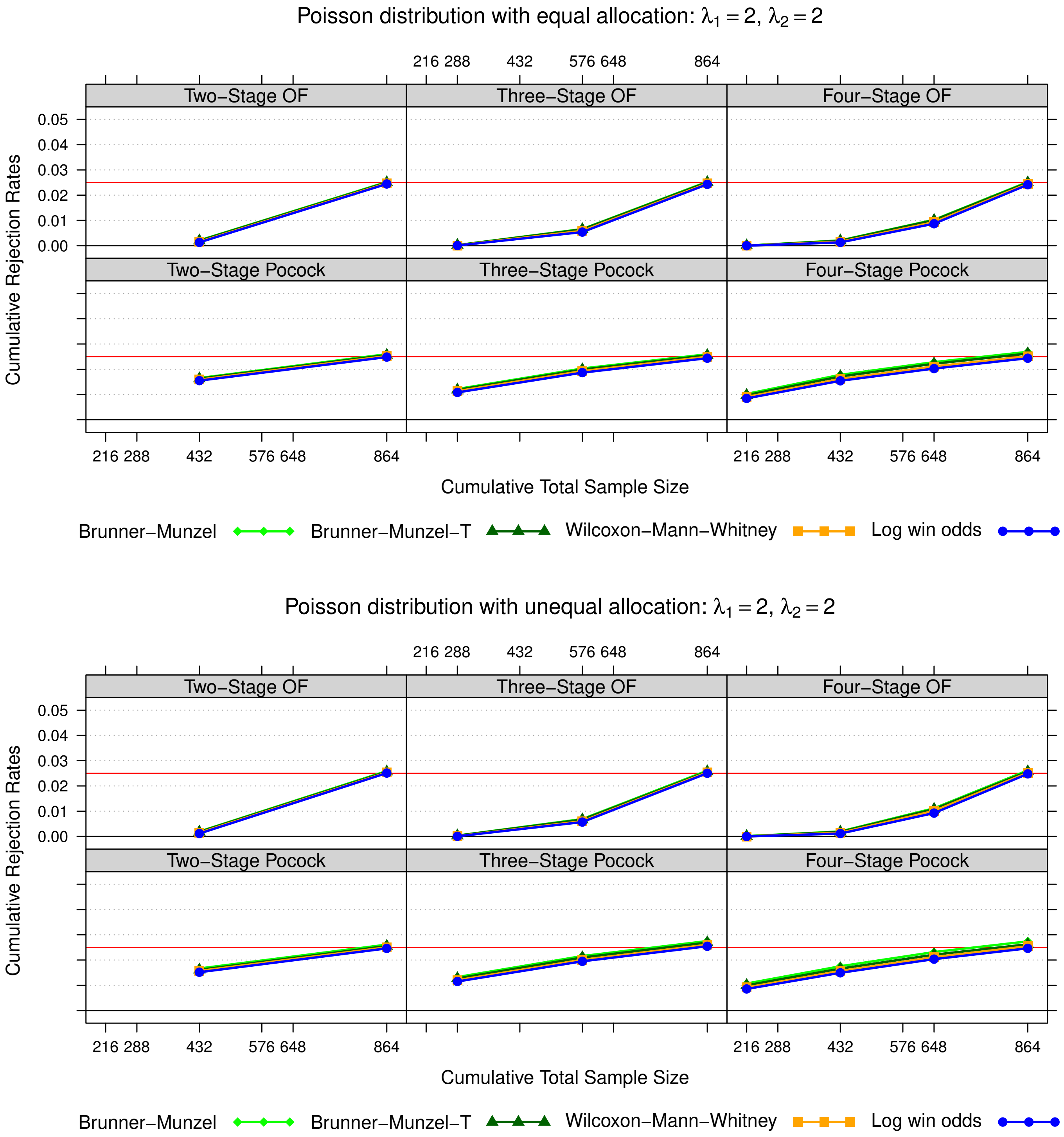

Figure 37: Poisson distribution with total maximum sample size 864

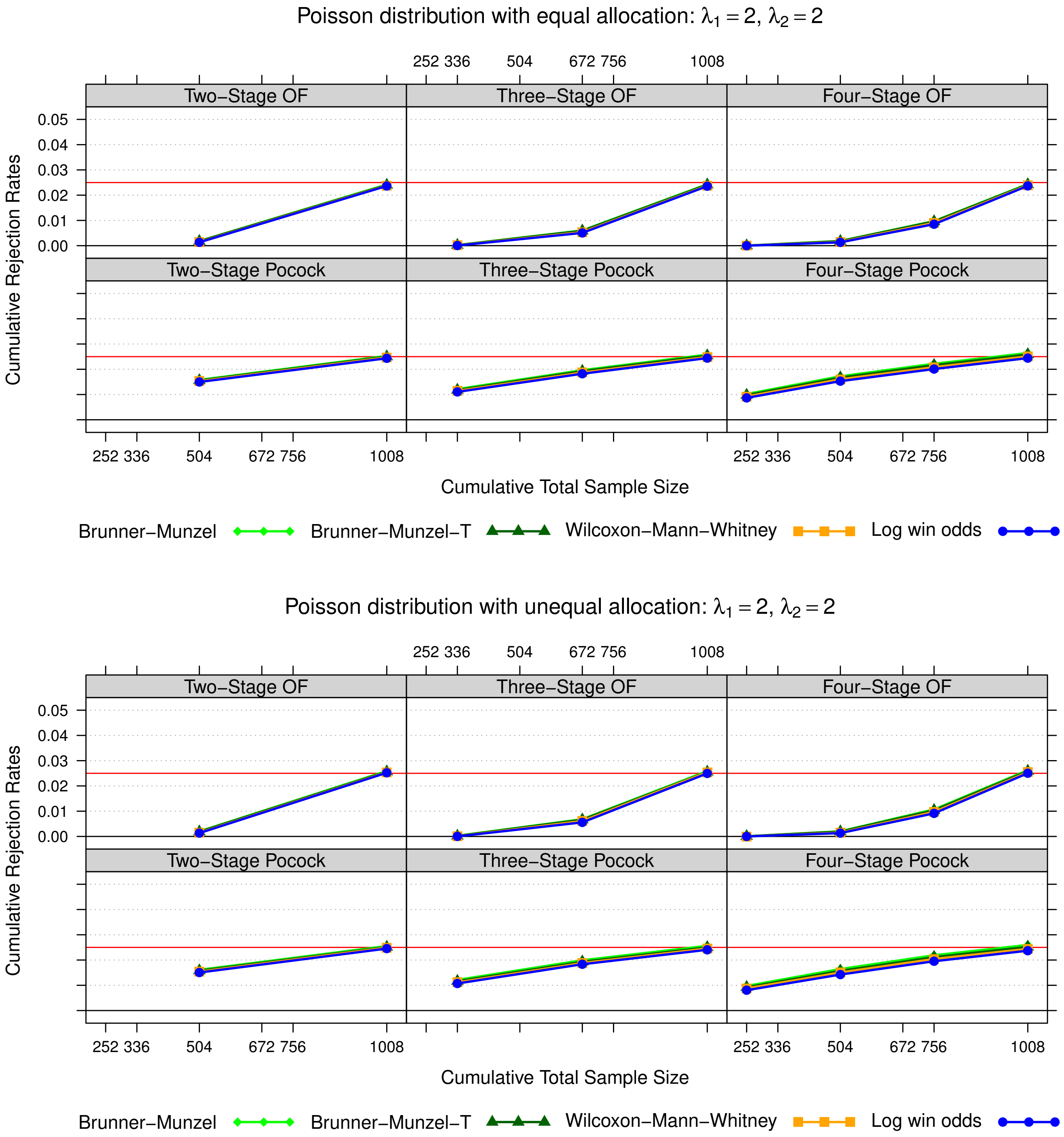

Figure 38: Poisson distribution with total maximum sample size 1008
